# Conservation and divergence of vulnerability and responses to stressors between human and mouse astrocytes

**DOI:** 10.1038/s41467-021-24232-3

**Published:** 2021-06-25

**Authors:** Jiwen Li, Lin Pan, William G. Pembroke, Jessica E. Rexach, Marlesa I. Godoy, Michael C. Condro, Alvaro G. Alvarado, Mineli Harteni, Yen-Wei Chen, Linsey Stiles, Angela Y. Chen, Ina B. Wanner, Xia Yang, Steven A. Goldman, Daniel H. Geschwind, Harley I. Kornblum, Ye Zhang

**Affiliations:** 1 Department of Psychiatry and Biobehavioral Sciences, Semel Institute for Neuroscience and Human Behavior, David Geffen School of Medicine at the University of California, Los Angeles, CA USA; 2 Department of Neurology, David Geffen School of Medicine at the University of California, Los Angeles, CA USA; 3 Department of Integrative Biology and Physiology, University of California, Los Angeles, CA USA; 4 Department of Endocrinology, David Geffen School of Medicine at the University of California, Los Angeles, CA USA; 5 Department of Obstetrics and Gynecology, University of California, Los Angeles, CA USA; 6 Intellectual and Developmental Disabilities Research Center at UCLA, Los Angeles, CA USA; 7 Institute for Quantitative and Computational Biosciences at UCLA, Los Angeles, CA USA; 8 Brain Research Institute at UCLA, Los Angeles, CA USA; 9 Molecular Biology Institute at UCLA, Los Angeles, CA USA; 10 Center for Translational Neuromedicine and Department of Neurology, University of Rochester Medical Center, Rochester, NY USA; 11 Center for Translational Neuromedicine, University of Copenhagen Faculty of Health and Medical Sciences, Copenhagen, Denmark; 12 Department of Human Genetics, David Geffen School of Medicine at the University of California, Los Angeles, CA USA; 13 Department of Pediatrics, David Geffen School of Medicine at the University of California, Los Angeles, CA USA; 14 Eli and Edythe Broad Center of Regenerative Medicine and Stem Cell Research at UCLA, Los Angeles, CA USA

**Keywords:** Transcriptomics, Astrocyte, Molecular neuroscience

## Abstract

Astrocytes play important roles in neurological disorders such as stroke, injury, and neurodegeneration. Most knowledge on astrocyte biology is based on studies of mouse models and the similarities and differences between human and mouse astrocytes are insufficiently characterized, presenting a barrier in translational research. Based on analyses of acutely purified astrocytes, serum-free cultures of primary astrocytes, and xenografted chimeric mice, we find extensive conservation in astrocytic gene expression between human and mouse samples. However, the genes involved in defense response and metabolism show species-specific differences. Human astrocytes exhibit greater susceptibility to oxidative stress than mouse astrocytes, due to differences in mitochondrial physiology and detoxification pathways. In addition, we find that mouse but not human astrocytes activate a molecular program for neural repair under hypoxia, whereas human but not mouse astrocytes activate the antigen presentation pathway under inflammatory conditions. Here, we show species-dependent properties of astrocytes, which can be informative for improving translation from mouse models to humans.

## Abstract (web summary)

Astrocytes are important players in brain development, homeostasis, and disease. Here, the authors compare the transcriptional profiles of human and mouse astrocytes. They report species-specific susceptibility to oxidative stress and response to hypoxic and inflammatory conditions.

## Introduction

Mice are one of the most widely used experimental animals in biomedical research due to the ease with which they can be genetically manipulated and the many paradigms that translate well between species. However, humans and mice differ greatly in body size, life span, ecological niche, behavior, and pathogenic challenges. Many mouse models of neurodegenerative disorders exhibit milder neuron degeneration phenotypes compared with human patients^1–4^. Mouse models of ischemic stroke can often achieve full functional recovery^5^, whereas human patients frequently have irreversible functional deficits. These limitations represent a key barrier in translational research, as over 90% of neurological drug candidates with promising animal data failing in human clinical trials^6^. Therefore, a full understanding of the cellular and molecular differences between the human and mouse brain is urgently needed.

Astrocytes are critical for many aspects of development and function in the central nervous system (CNS)^7–23^. Most of our knowledge on the biology of astrocytes is based on studies using mouse astrocytes. Aside from human astrocytes being larger and morphologically more complex than mouse astrocytes^24,25^, little is known about the similarities and differences between human and mouse astrocytes, particularly their responses to disease-relevant perturbations. Because of this knowledge gap, it is challenging to harness the knowledge gained from mouse astrocytes to elucidate the biology of human astrocytes and their roles in neurological disorders.

In this study, we systematically examined human astrocytes under three conditions: acutely purified, cultured without serum, and xenografted into mouse brains. We found extensive conservation between human and mouse astrocyte transcriptomes, but also identified important differences between mouse and human astrocytes that were maintained across all three conditions. We identified striking differences in the cell survival, mitochondrial physiology, and molecular responses of human and mouse astrocytes under oxidative stress, hypoxia, inflammatory cytokine treatment, and simulated viral infections. These findings reveal important mechanistic differences between human and mouse astrocytes and provide insight into how mouse models of neurodegeneration and stroke can be improved to achieve better translation to humans.

## Results

### Immunopanned astrocytes exhibit resting transcriptome profiles

**Fig. 1:**
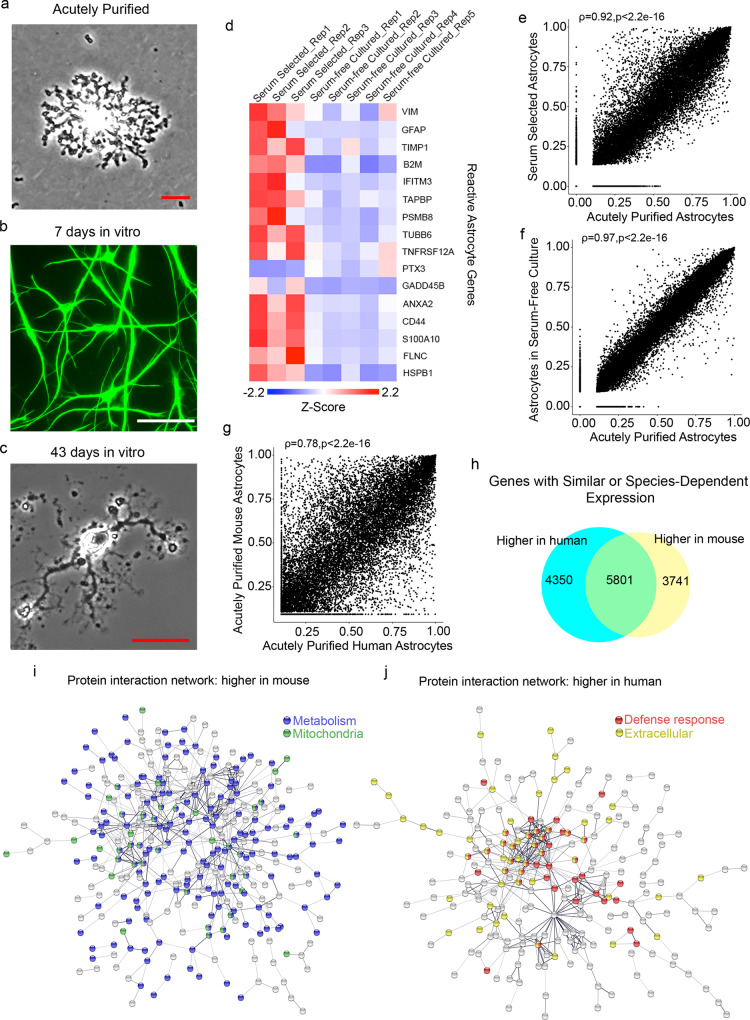
Comparison of astrocyte transcriptomes in vivo and in vitro and between human and mouse. **a** An astrocyte bound to an anti-HepaCAM antibody-coated petri dish during immunopanning purification. RNA was extracted immediately after the cells stuck to the dish. These samples are referred to as acutely purified. Scale bar: 10 μm. **b** Astrocytes in serum-free culture stained with anti-GFAP antibodies. Scale bar: 50 μm. **c** A bright-field image of an astrocyte in serum-free culture. Scale bar: 20 μm. **d** Expression of reactive astrocyte marker genes in serum-selected and serum-free cultures of human astrocytes. Z-score is calculated as (RPKM—average RPKM across all samples)/standard deviation. Genes with FDR < 0.1 between serum-selected and serum-free cultures and RPKM > 1.5 are shown. **e**, **f** Scatter plots and Spearman’s correlation coefficients (ρ) of gene expression between cultured and acutely purified human astrocytes using the serum-selected culture method and our serum-free culture method. For each condition, gene expression across 3–5 patient samples was averaged. Only protein-coding genes were included. Two-tailed t-test. *p* < 2.2×10^−16^. **g** Scatter plot and Spearman’s correlation of gene expression between acutely purified human and mouse astrocytes. Two-tailed t-test. *p* < 2.2×10^−16^. Human brain tissue was derived from donors of different ages (8–63 years). Mouse brain tissue was derived from postnatal and adult mice. Additional information is provided in Supplementary Data 8. **h** Number of genes with similar or species-dependent expression. Genes with percentile rankings in the top two-thirds were included in this analysis to eliminate those not expressed or expressed at very low levels. Percentile rankings of the expression of each gene were compared across human and mouse astrocyte samples and differences were tested by Welch’s T-test followed by post-hoc multiple comparison adjustment using the Benjamini and Hochberg FDR method^124–126^. FDR < 0.05.** i**, **j** Protein interaction networks of genes expressed at higher levels by mouse astrocytes than human astrocytes (**i**) and at higher levels by human astrocytes than mouse astrocytes (**j**) (percentile ranking difference > 0.4). FDR < 0.05. Blue: genes associated with the GO term metabolism. Green: genes associated with the cellular component mitochondria. Red: genes associated with the GO term defense response. Yellow: genes associated with the cellular component extracellular.

We recently developed an immunopanning method for the acute purification of human astrocytes and a serum-free chemically defined medium that keeps human astrocytes healthy for at least six weeks in vitro (Fig. 1a–c)^26^. Here, we tested whether immunopanned human astrocytes resemble resting or reactive astrocytes by RNA sequencing (RNA-seq). We assessed the expression of genes previously found to be induced by stroke and inflammation in mouse astrocytes^27^. The expression of these genes was significantly lower in cultured immunopanned astrocytes than in serum-selected astrocytes (average fold change = 0.18; false discovery rate (FDR) = 0.032; Fig. 1d). In addition, we identified reactive astrocyte genes induced in inflammatory conditions in humans (see below) and again found lower expression of these genes in cultured immunopanned astrocytes than in serum-selected astrocytes (Supplementary Fig. 1). Both immunopanned and serum-selected human astrocyte cultures exhibited high expression of astrocyte-specific genes and low or undetectable levels of genes specific to neurons, microglia, oligodendrocyte precursor cells, or endothelial cells (Supplementary Fig. 2).

To examine the extent to which immunopanned human astrocytes could model in vivo astrocytes, we performed immunopanning purification of human astrocytes and harvested RNA (1) immediately after purification to capture the in vivo gene signature (referred to as acutely purified thereafter) and (2) after 4–6 days of culturing in our serum-free chemically defined medium (referred to as serum-free cultured thereafter). We then performed RNA-seq and compared the transcriptomes of acutely purified and cultured human astrocytes. We found that the gene expression from the serum-free astrocyte cultures more closely resembled acutely purified astrocytes than astrocytes obtained using the traditional serum-selected method (Spearman’s correlation = 0.97 vs. 0.92; these correlation coefficients are significantly different; *p* < 0.0001; Fig. 1e, f, Supplementary Fig. 3 and Supplementary Data 1 and 2). We performed principal component analysis (PCA) and found that acutely purified astrocytes and serum-free cultures of astrocytes are more similar to each other than to serum-selected astrocytes (Supplementary Fig. 4). Overall, immunopanned human astrocytes recapitulate the expression of the majority of genes expressed by astrocytes in vivo and therefore represent a useful platform for studying human astrocyte biology.

### Species-dependent astrocytic gene expression

We compared the acutely purified human astrocytes described above with corresponding mouse transcriptome data that we previously collected^26,28^. The overall gene expression profiles showed conservation between human and mouse astrocytes (Spearman’s correlation ρ = 0.78; Fig. 1g). However, thousands of genes exhibited significant differences in expression between species (8091 genes, FDR < 0.05; genes with percentile rankings in the top two-thirds of both species were included; Fig. 1h; Supplementary Fig. 5). To pinpoint the genes and pathways that differed between human and mouse astrocytes, we analyzed protein-interaction networks and gene ontology (GO) terms. We found that the genes expressed at higher levels by mouse compared to human astrocytes were enriched in multiple GO terms associated with metabolism (Fig. 1i and Supplementary Data 3). In contrast, genes expressed at higher levels by human compared to mouse astrocytes were enriched in a single GO term, defense response (Fig. 1j and Supplementary Data 3). We analyzed the subcellular localization of proteins encoded by genes differentially expressed between human and mouse astrocytes. Interestingly, mouse astrocytes showed higher expression of genes associated with the compartment mitochondria, whereas human astrocytes showed higher expression of genes assigned to extracellular space (Fig. 1i, j), including secreted cytokines. The top hub genes with the most protein-protein interactions with other genes in the network include *IL6*, a cytokine involved in inflammation, and *TLR4*, a Toll-like family receptor that mediates responses to bacterial lipopolysaccharide, in the genes with higher expression in humans, and *Ndufa7* and *Ndufb7*, mitochondrial respiratory chain components, in the genes with higher expression in mouse (Supplementary Data 4). To assess whether gene expression differences between human and mouse astrocytes can be found in other independent datasets, we analyzed single-cell and single-nucleus RNA-seq datasets from human and mouse brains^29^. We found that the human-mouse expression differences determined from our immunopanned astrocyte bulk RNA-seq data correlate with the human-mouse expression differences derived from single-cell RNA-seq data (*r*^2^ = 0.35; Supplementary Fig. 6), confirming that our approach of using acutely purified astrocytes from human and mouse can recapitulate the species differences in astrocytes observed in vivo. The human-mouse gene expression differences we identified are consistent across 15 mouse strains (Supplementary Fig. 7).

### The human-specific gene signature is intrinsically programmed

**Fig. 2:**
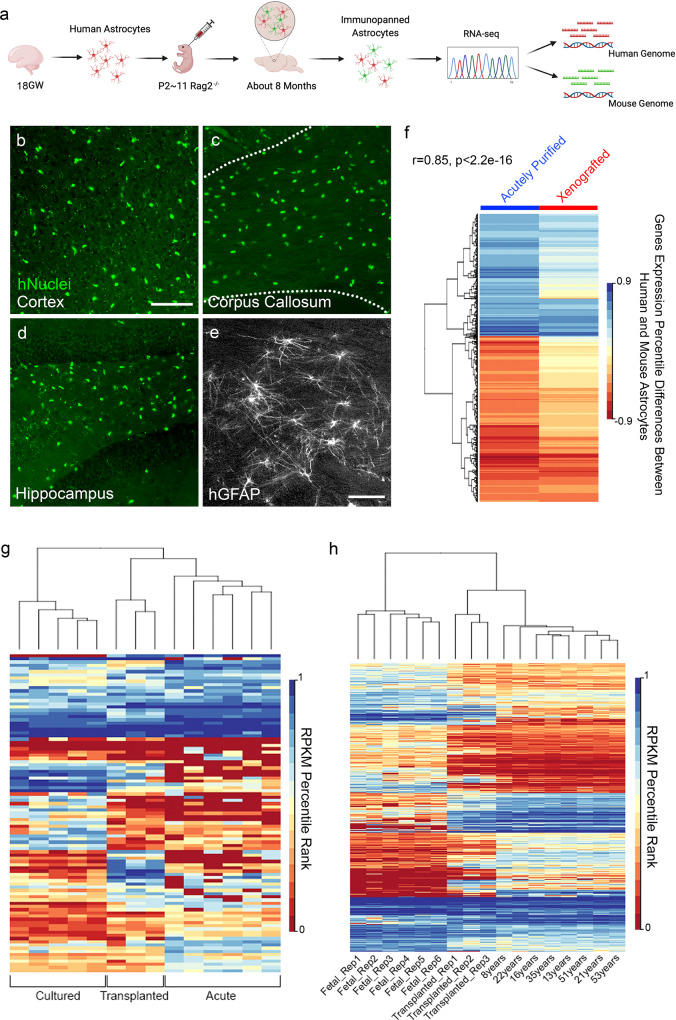
The human-specific astrocyte gene signature is intrinsically programmed. **a** Experimental design. Gestational week 18 primary human astrocytes were purified and injected into the brains of neonatal immunodeficient Rag2-knockout mice. After about 8 months, we purified astrocytes from xenografted mouse brains by immunopanning. The astrocytes from both human grafts and mouse hosts were sequenced together and reads were mapped to human and mouse genomes, respectively. GW, gestational week. P, postnatal day. **b**–**d** Xenografted human cells in host mouse brains stained with an antibody against human nuclei (green). Scale bar: 100 μm. Dashed lines delineate the corpus callosum. **e** Xenografted human astrocytes in host mouse brains stained with an anti-GFAP antibody that only reacts with human GFAP but not mouse GFAP. Scale bar: 50 μm. **f** Species differences in gene expression (shown as percentile ranking in human minus percentile ranking in mouse) in xenografted and acutely purified astrocytes highly correlate. Genes with percentile rankings > 0.33, species differences with FDR < 0.05, and species differences in percentile ranking > 0.4 are shown. r, Pearson’s correlation coefficient. **g** Non-supervised hierarchical clustering of gene expression in serum-free cultures, acutely purified astrocytes, and transplanted human astrocytes. Genes with significant differences between cultured and acutely purified astrocytes (FDR < 0.05, fold change > 4, average RPKM > 1) are shown. **h** Non-supervised hierarchical clustering of gene expression of acutely purified astrocytes from patients of different ages and transplanted human astrocytes. Genes with significant differences between age groups (fetal, child, adult; FDR < 0.05, fold change > 2, maximum RPKM > 1) are shown.

The higher expression of defense response genes by human astrocytes could be a result of either intrinsic properties or differences in external factors (e.g., neuronal or glial cell types, systemic or environmental variations) between human and mouse samples. To assess differences between mouse and human astrocytes when exposed to equivalent external environments, we transplanted human astrocytes into mouse brains and compared them with the neighboring host mouse astrocytes^30–32^. We purified primary human fetal astrocytes, and then injected them into the brains of neonatal mice (Fig. 2a). We aged the xenografted chimera mice for about 8 months, and then confirmed widespread distribution of human astrocytes in the host mouse brains (Fig. 2b–e). We then purified all astrocytes (human and mouse) from the chimeric mice by immunopanning and performed RNA-seq. We exploited DNA sequence differences between human and mouse genes in order to separate sequencing reads of human vs. mouse origin at the mapping step. This approach allowed us to obtain the transcriptome profile of human astrocytes grafted in a host mouse brain (Supplementary Data 5).

To test whether the human-specific astrocyte gene signature is intrinsically programmed or induced by other cell types in the human brain environment, we compared gene expression differences between human and mouse astrocytes using both the acutely purified and the xenograft/host dataset. We reasoned that human-mouse astrocyte differences would be attenuated in the xenograft model if the astrocyte differences were driven by human-specific environmental factors. We calculated gene expression differences between human and mouse astrocytes based on the acutely purified dataset (hmDiff_acute) and the chimera dataset (hmDiff_chimera) (Fig. 2f; Supplementary Fig. 8). The heatmaps (Fig. 2f; Supplementary Fig. 8) show differentially expressed genes based on these two datasets. If the species-specific gene expression patterns were determined by the host environment, then differentially expressed genes across species based on the acutely purified dataset would not be differentially expressed in the xenografted dataset (i.e., the xenografted column of the heatmap would appear white). Instead, we observed similar patterns of species-specific gene expression across the acutely purified and xenografted datasets. We found a positive correlation between hmDiff_acute and hmDiff_chimera (Pearson’s correlation = 0.60 for all genes; Pearson’s correlation = 0.85 for genes with percentile differences > 0.4; Fig. 2f and Supplementary Fig. 8). We also calculated gene expression correlations between transplanted human astrocytes and acutely purified human/mouse astrocytes and found that transplanted human astrocytes resemble human astrocytes more than mouse astrocytes (i.e., the correlation coefficient of transplanted human vs. acutely purified human was significantly higher than that of transplanted human vs. acutely purified mouse; *p* < 0.0001; Supplementary Fig. 9a, b). These analyses suggest that the human-specific astrocyte gene signature is largely intrinsically programmed, with only minor environmental contributions by neurons and other cell types in the human brain.

One challenge that has limited human astrocyte research is the difficulty in obtaining mature cells for experimental manipulations, due to (1) the limited availability of fresh healthy adult human brain tissue and (2) the restriction that stem cell-derived human astrocytes mostly resemble developing stages^33–35^. Here, we found that certain genes that are expressed in acutely purified astrocytes in vivo, but lost in culture, are regained in xenografted astrocytes (Fig. 2g). In fact, the xenografted human astrocytes were able to reach mature stages that are difficult to access in in vitro models (Fig. 2h; Supplementary Fig. 9c–e), allowing us to observe the persistence of mouse and human transcriptomic differences across a broad developmental range. Therefore, in addition to identifying consistent species differences in astrocyte transcriptomic profiles across acutely purified, cultured, and xenografted conditions, we established the xenograft model as a much-needed platform for studying mature human astrocytes in vivo.

To assess how the introduction of human astrocytes may change host mouse astrocytes, we compared the transcriptome of host mouse astrocytes (this study) with naïve mouse astrocytes from a similar age^26^ and found differentially expressed genes (Supplementary Data 6 and 7).

### Species-dependent susceptibility to oxidative stress

To examine responses of human and mouse astrocytes to environmental perturbations, we treated human and mouse astrocytes with several disease-relevant stimuli—including oxidative stress, hypoxia, simulated viral infection, and an inflammatory cytokine—and evaluated the responses.

Oxidative stress is produced by reactive oxygen species (ROS) such as peroxides, superoxide, hydroxyl radical, singlet oxygen, and alpha-oxygen. ROS are byproducts of normal metabolism in most cell types in the body. Importantly, during pathogen invasion, tissue damage, and inflammation, immune cells such as neutrophils and macrophages produce high levels of ROS that help fend off pathogen infections but may also damage healthy cells in infected tissue. In the brain, oxidative stress is a key pathological process underlying neurodegenerative disorders (such as Alzheimer’s disease, Parkinson’s disease, Huntington’s disease, and amyotrophic lateral sclerosis), stroke, and traumatic injury.

To examine responses of human and mouse astrocytes to oxidative stress, we purified human and mouse astrocytes from developmentally equivalent stages [gestational week 17–20 for human brains and postnatal day 1–3 (P1–3) for mouse brains; see Methods for details on developmental stage matching]. Astrocytes have been shown to be regionally heterogeneous^36^. Therefore, whenever possible, we matched anatomical locations in human and mouse brains. We used whole cerebral cortex for astrocyte purification for all mouse samples and a subset of human samples with a clearly identifiable cerebral cortex. In cases where identification of cerebral cortex was difficult due to tissue fragmentation, we selected, to the best of our ability, fragments most likely to be cortex (large thin sheets).

**Fig. 3:**
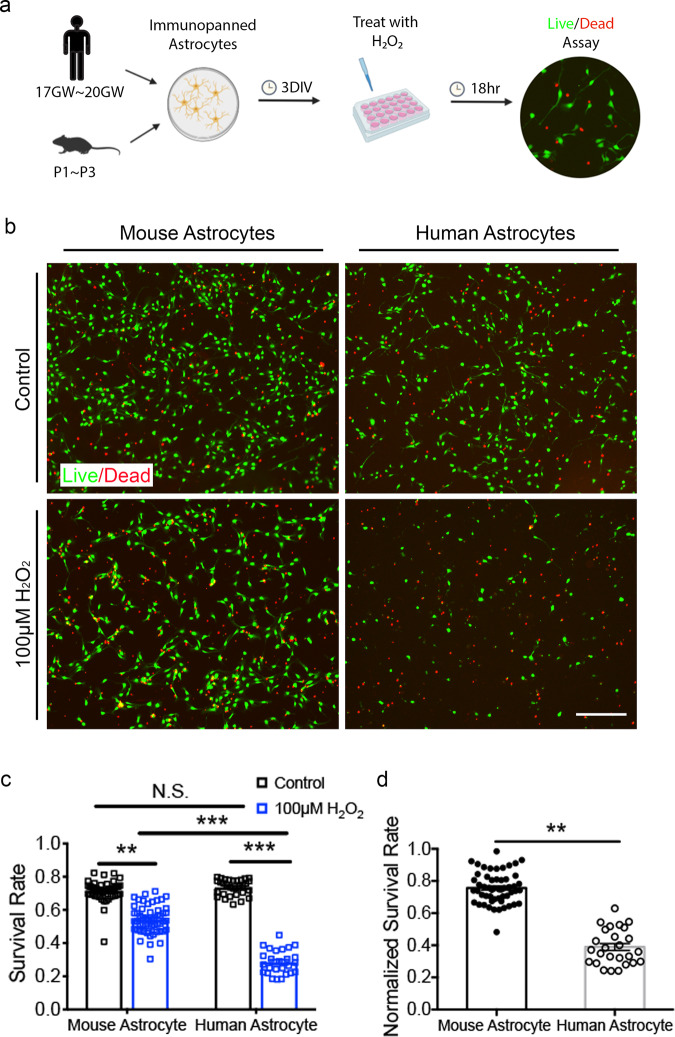
Human astrocytes are more susceptible to oxidative stress than mouse astrocytes. **a** Experimental design. Hr, hour. **b** Human and mouse astrocytes treated with H_2_O_2_ or medium control stained with the live cell dye calcein-AM (green) and the dead cell dye ethidium homodimer (red). Scale bar: 200 μm. **c** Survival rate. Mouse astrocytes: *N* = 50 images from 12 cultures treated with medium control and 54 images from 12 cultures treated with H_2_O_2_ generated from 6 litters of mice. Human astrocytes: *N* = 31 images from 7 cultures treated with medium control and 26 images from 6 cultures treated with H_2_O_2_ generated from 3 patients. Data are presented as mean ± SEM in all figures, unless otherwise indicated. Mouse astrocytes: control vs. H_2_O_2_, *p* = 0.0039. H_2_O_2_-treated mouse astrocytes vs. H_2_O_2_-treated human astrocytes, *p* = 0.0002. Human astrocytes: control vs. H_2_O_2_, *p* < 0.0001. *p < 0.05, **p < 0.01, ***p < 0.001 in all figures. Two-way analysis of variance (ANOVA) with Tukey’s test for multiple comparisons. N.S., not significant. The *p*-values were calculated using average results from each litter of mice and each patient as independent observations, unless otherwise indicated. **d** Survival rate of astrocytes treated with H_2_O_2_ normalized to the survival rate of medium control-treated cells. Replicate numbers N are defined in (**c**). *p* = 0.0096. Two-tailed unpaired Welch’s *t*-test.

We performed immunopanning purification to obtain human and mouse astrocytes, plated them at similar densities, and cultured them using identical growth media. To examine responses of human and mouse astrocytes to oxidative stress, we treated cells cultured 3 days in vitro (div) with 100 μM H_2_O_2_. We then examined cell survival by staining with the live-cell dye calcein-AM and the dead-cell dye ethidium homodimer, 18 h after treatment onset (Fig. 3a). We found that human astrocytes were much more susceptible than mouse astrocytes to oxidative stress (survival rates: 0.29 ± 0.01 for human astrocytes and 0.54 ± 0.01 for mouse astrocytes; Fig. 3b–d; *p* < 0.001; data represent average ± SEM unless otherwise noted). Since plating density, H_2_O_2_ concentration, and treatment duration may affect cell survival, we tested different combinations of these conditions and again found that human astrocytes were much more susceptible than mouse astrocytes to oxidative stress (Supplementary Fig. 10).

### Species differences in mitochondrial respiration

Mitochondria are both sources and targets of ROS: the mitochondrial respiration chain produces ROS, and ROS (either endogenous or exogenous) can damage mitochondrial function. Furthermore, mitochondria play important roles in cell death. Therefore, to identify the cellular mechanisms underlying the striking difference in the susceptibilities of human and mouse astrocytes to oxidative stress, we examined mitochondrial metabolism in these cells. We purified human and mouse astrocytes, cultured them under the same conditions and assessed mitochondrial metabolism.

**Fig. 4:**
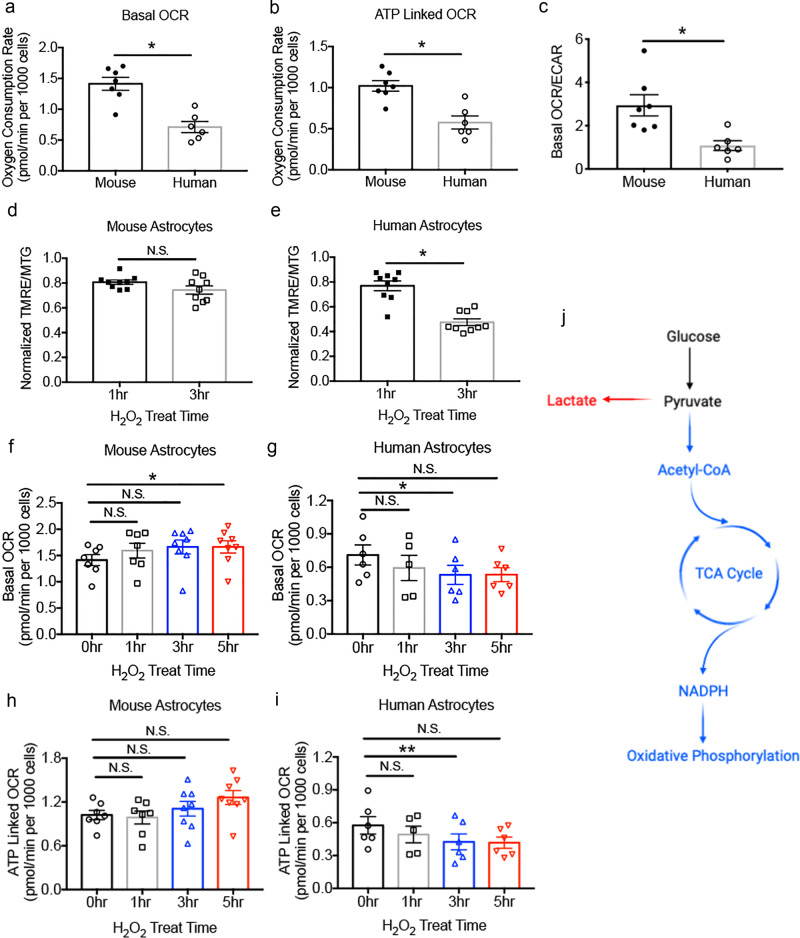
Mitochondrial metabolism differences between human and mouse astrocytes. **a **Basal oxygen consumption rate (OCR) of mouse and human astrocytes. Each data point (circle or square) represents one well of astrocyte culture prepared from one human patient or one litter of 8–10 mice throughout this figure. Mouse astrocytes: N = 7 cultures generated from 4 litters of mice in **a**–**c**. Human astrocytes: *N* = 6 wells of cultured cells generated from 3 patients in **a**–**c**. *p* = 0.0365. Two-tailed unpaired Welch’s t-test. The *p*-values were calculated using average results from each litter of mice and each patient as independent observations, unless otherwise indicated. **b** OCR linked to ATP production in the presence of oligomycin. *p* = 0.0497. Two-tailed unpaired Welch’s t-test. **c** The ratio of OCR to extracellular acidification rate (ECAR). *p* = 0.0168. Two-tailed unpaired Welch’s t-test. **d**, **e** Tetramethylrhodamine, ethyl ester (TMRE) fluorescence (reporting mitochondrial membrane potential) normalized by MitoTracker Green (MTG, a general mitochondrial dye) fluorescence. Data represent H_2_O_2_-treated conditions normalized to medium control-treated conditions. *N* = 9 wells of cultured cells generated from 3 litters of mice and 4 patients. Mouse astrocytes: 3 hr vs. 1 hr, *p* = 0.3959. Human astrocytes: 3 hr vs. 1 hr, *p* = 0.0373. Two-tailed unpaired Welch’s t-test. N.S., not significant. **f**, **g** Basal OCR of astrocytes treated with 100 μM H_2_O_2_. 0 hr and 1 hr mouse astrocytes: *N* = 7 wells of cultured cells generated from 4 litters of mice. 3 hr and 5 hr mouse astrocytes: *N* = 8 wells of cultured cells generated from 4 litters of mice. 0 hr, 3 hr, and 5 hr human astrocytes: *N* = 6 wells of cultured cells generated from 3 patients. 1 hr human astrocytes: *N* = 5 wells of cultured cells generated from 3 patients. Mouse astrocytes: 0 hr vs. 1 hr, *p* = 0.1883; 0 hr vs. 3 hr, *p* = 0.4246; 0 hr vs. 5 hr, *p* = 0.0285. Human astrocytes: 0 hr vs. 1 hr, *p* = 0.0954; 0 hr vs. 3 hr, *p* = 0.0235; 0 hr vs. 5 hr, *p* = 0.1837. One-way repeated measure ANOVA with Dunnett’s multiple comparison test. **h**, **i** ATP-linked OCR of astrocytes treated with 100 μM H_2_O_2_. The replicate numbers are defined in (**f**–**g**). Mouse astrocytes: 0 hr vs. 1 hr, *p* = 0.8885; 0 hr vs. 3 hr, *p* = 0.7597; 0 hr vs. 5 hr, *p* = 0.2198. Human astrocytes: 0 hr vs. 1 hr, *p* = 0.1639; 0 hr vs. 3 hr, *p* = 0.0016; 0 hr vs. 5 hr, *p* = 0.2583. One-way repeated measure ANOVA with Dunnett’s multiple comparison test. **j** Diagram of glucose metabolism. TCA, tricarboxylic acid. NADPH, nicotinamide adenine dinucleotide phosphate.

Although human astrocytes are larger than mouse astrocytes in vivo^25^ and in vitro^26^, the basal respiration rate per mouse astrocyte (oxygen consumption rate, OCR) is almost twice as high compared with human astrocytes (mouse: 1.41±0.11 pmol/min per 1000 cells; human: 0.71±0.09 pmol/min per 1000 cells; *p* < 0.05; Fig. 4a). Furthermore, respiration for ATP production is also higher in mouse than human astrocytes (mouse: 1.02±0.06 pmol/min per 1000 cells; human 0.58±0.08 pmol/min per 1000 cells; *p* < 0.05. Fig. 4b). Human and mouse astrocytes were plated at similar densities and cultured with identical media for all metabolic experiments (Supplementary Fig. 11).

These differences in the mitochondrial respiration rates in human and mouse astrocytes raised the question of whether energy substrates are utilized differently in these cells. Glucose is the predominant energy substrate in healthy brains. Through glycolysis, glucose becomes pyruvate, leading to two alternative metabolic pathways (Fig. 4j): (1) Pyruvate can be converted to acetyl-CoA, enter the tricarboxylic acid cycle, and eventually be converted to substrates of oxidative phosphorylation and produce ATP. This process occurs intracellularly within astrocytes. (2) Pyruvate can be converted to lactate and exported to the extracellular space. Neurons can take in lactate and use it as an energy substrate, although the astrocyte-neuron-lactate-shuttle hypothesis remains controversial. To examine the usage of glucose by the two alternative pathways in human and mouse astrocytes, we used Seahorse Respirometry’s pH-sensitive electrodes to measure the extracellular acidification rate (ECAR), an approximate measure of lactate production and the glycolysis rate, and compared the ECAR to OCR, an approximate measure of the oxidative phosphorylation rate. We found that the OCR/ECAR ratio was higher in mouse astrocytes than in human astrocytes (Fig. 4c). Thus, mouse astrocytes may utilize a larger proportion of glucose for oxidative phosphorylation, which provides energy for astrocytes themselves, whereas human astrocytes utilize a larger proportion of glucose for lactate production, which may serve as an energy substrate for neurons.

Having identified metabolic differences between human and mouse astrocytes in unperturbed conditions, we next examined changes in mitochondrial metabolism and physiology under oxidative stress in human and mouse astrocytes. We treated the astrocytes with 100 μM H_2_O_2_ and measured OCR 1, 3, and 5 h after treatment onset. While mouse astrocytes exhibited a small increase, human astrocytes exhibited a substantial reduction of OCR under oxidative stress (Fig. 4f, g; mouse: 0 hr 1.41 ± 0.11, 5 hr 1.66 ± 0.12, *p* < 0.05; human: 0 hr 0.71 ± 0.09, 3 hr 0.53 ± 0.08, *p* < 0.05). Similarly, we found that ATP-linked OCR is stable in mouse astrocytes but reduced in human astrocytes under oxidative stress (Fig. 4h, i). Therefore, mitochondria from mouse astrocytes are highly resilient to oxidative damage; these organelles may work harder as an adaptive response to oxidative damage and, as a result, produce more ATP for cellular protective pathways (see section on the detoxification pathway below). In contrast, mitochondria from human astrocytes are quickly damaged and cannot keep up with the cellular energy demand when exposed to oxidative stress.

To further examine the physiological status of mitochondria under oxidative stress, we performed fluorescence imaging using tetramethylrhodamine ethyl ester (TMRE), a dye sensitive to the membrane potential across the mitochondrial inner membrane^37^. We found that the mitochondrial membrane potential remained largely stable in mouse astrocytes but depolarized quickly in human astrocytes (Fig. 4d, e; mouse 0.81 ± 0.02 at 1 hr, 0.74 ± 0.03 at 3 hr, not significant; human 0.77 ± 0.04 at 1 hr, 0.48 ± 0.03 at 3 hr, *p* < 0.05). Therefore, mitochondria in human astrocytes are more susceptible to oxidative damage than those in mouse astrocytes.

### Species-dependent expression of detoxification pathway genes

**Fig. 5:**
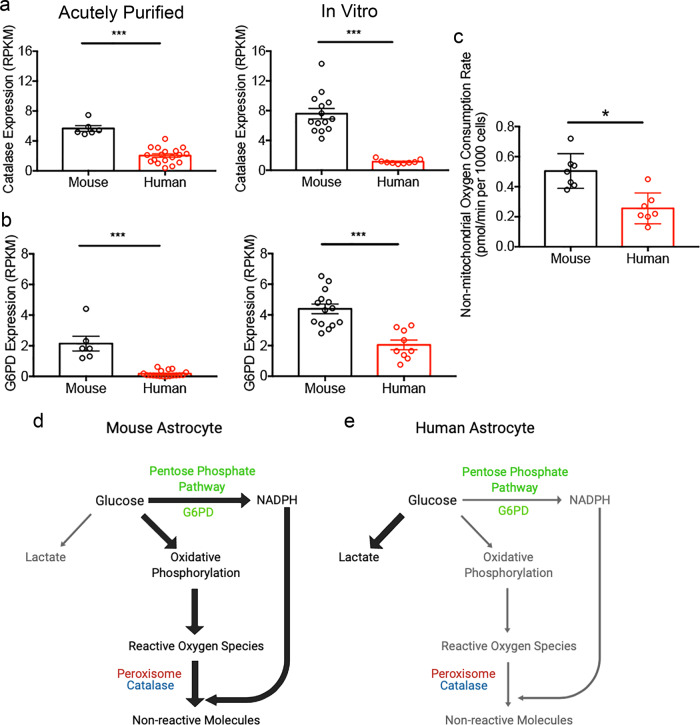
Detoxification pathway differences between human and mouse astrocytes. **a**, **b **Expression of ROS detoxification pathway genes catalase (**a**) and glucose-6-phosphate dehydrogenase (G6pd in human/G6pdx in mouse) (**b**) by acutely purified astrocytes and serum-free cultures of astrocytes determined by RNA-seq. *N* = 6 litters of mice and 18 human patients for acutely purified samples. *N* = 14 litters of mice and 9 human patients in vitro. Acutely purified: Catalase, *p* < 0.0001; G6PD, *p* < 0.0001, two-tailed Mann-Whitney test. Serum-free cultures: Catalase, *p* < 0.0001; G6PD, *p* < 0.0001. Two-tailed unpaired Welch’s t-test, unless otherwise indicated. Samples include children and adult patients as well as developing and adult mice. The ages of the patients and mice are listed in Supplementary Data 8. **c **Non-mitochondrial OCR measured in the presence of antimycin-A. *N* = 7 wells of cultured cells from each species generated from 4 litters of mice and 3 patients. *p* = 0.0126. Two-tailed unpaired Welch’s t-test. The *p*-values were calculated using average results from each litter of mice and each patient as independent observations. **d**, **e** Model of glucose metabolism and detoxification pathways in human and mouse astrocytes. The widths of the arrows represent the rate of the metabolic processes. Mouse astrocytes have higher rates of oxidative phosphorylation, which presumably produce more ROS than human astrocytes. The higher abundance of detoxification pathway genes and the higher peroxisomal activity in mouse compared to human may protect the cells against oxidative damage.

The species differences in oxidative stress susceptibility may be because mouse astrocytes have evolved adaptive mechanisms, such as more efficient detoxification pathways, under high ROS conditions that render protection against oxidative stress. To test this hypothesis, we examined the function of the peroxisome, an organelle involved in ROS detoxification^38^. We blocked mitochondrial oxidation with Antimycin A, which binds and inactivates Complex III^39^, to focus on non-mitochondrial oxygen consumption, which has a large contribution from peroxisomal oxidation^40^. We found that the non-mitochondrial oxygen consumption rate was higher in mouse astrocytes than in human astrocytes (Fig. 5c), consistent with the possibility that peroxisome oxidation operates faster in mouse astrocytes than human astrocytes. To evaluate molecular differences in ROS detoxification pathways, we purified human and mouse astrocytes by immunopanning^26,28,41^ and performed RNA-seq immediately after purification and after 5–6 days of culture in serum-free conditions (this study). We found that the gene encoding a major peroxisomal ROS detoxification enzyme, catalase^42^, is expressed at 3–6-fold higher levels by mouse astrocytes than by human astrocytes [reads per kilobase per million mapped reads (RPKM): acutely purified, 2.04±0.24 for human astrocytes and 5.67±0.38 for mouse astrocytes; in vitro, 1.14±0.10 for human astrocytes and 7.60±0.71 for mouse astrocytes; Fig. 5a]. An additional molecular pathway, the pentose phosphate pathway, produces NADPH, which neutralizes ROS^43^. The rate-limiting step of the pentose phosphate pathway is catalyzed by glucose-6-phosphate dehydrogenase (G6PD), an important antioxidant enzyme^44^. Using RNA-seq, we found that *G6PD* gene expression is 2–10-fold higher in mouse astrocytes than in human astrocytes (RPKM: acutely purified, 0.17±0.05 for human astrocytes and 2.14±0.48 for mouse astrocytes; in vitro, 2.05±0.32 for human astrocytes and 4.39±0.32 for mouse astrocytes; Fig. 5b). The expression levels of *CAT* and *G6PD* were consistently higher in 15 strains of mice compared to humans (Supplementary Fig. 12). We also explored other major detoxification pathways and found generally comparable expression by human and mouse astrocytes. Taken together, higher amounts of catalase and G6PD may protect mouse astrocytes from oxidative stress (Fig. 5d, e).

As we performed all our in vitro functional experiments using developing astrocytes, we next obtained RNA-seq data from adult human and mouse astrocytes (Supplementary Data 8). Notably, the species differences persisted throughout development and adulthood (Fig. 5a, b). Oxidative stress is a core pathological process in a range of neurological conditions, including neurodegenerative disorders such as Alzheimer’s disease, Parkinson’s disease, Huntington’s disease, and amyotrophic lateral sclerosis. Mouse models of neurodegenerative disorders often have milder phenotypes compared to human patients^1–4^. Our findings suggest that differences in astrocytic responses to oxidative stress may contribute to the increased resiliency of mouse models of neurodegeneration compared to human patients (see Discussion).

To determine whether the lower expression of *catalase* and *G6pd* in humans is an astrocyte-specific attribute or a more general difference across species, we analyzed a single-cell RNA-seq dataset of human and mouse brains^45^ to examine the expression of these genes in all major cell types of the brain. We found lower expression of catalase and G6pd in humans than in mice in glutamatergic neurons, GABAergic neurons, oligodendrocyte precursor cells, and oligodendrocytes (Supplementary Fig. 12). Therefore, lower expression of catalase and G6pd is generally observed across multiple cell types in the human brain compared to the mouse brain.

### Hypoxia induces a molecular program for neural growth in mice

Adult mouse models of ischemic stroke often achieve full functional recovery^5^, whereas adult human stroke patients usually have irreversible functional deficits. Dozens of neural protective drug candidates that improved recovery in mouse models of stroke have failed to show benefits in human clinical trials^46^. Hypoxia is a key physical change in ischemic stroke. Responses of mouse astrocytes to hypoxia have been closely examined previously but responses of human astrocytes are largely unknown.

**Fig. 6:**
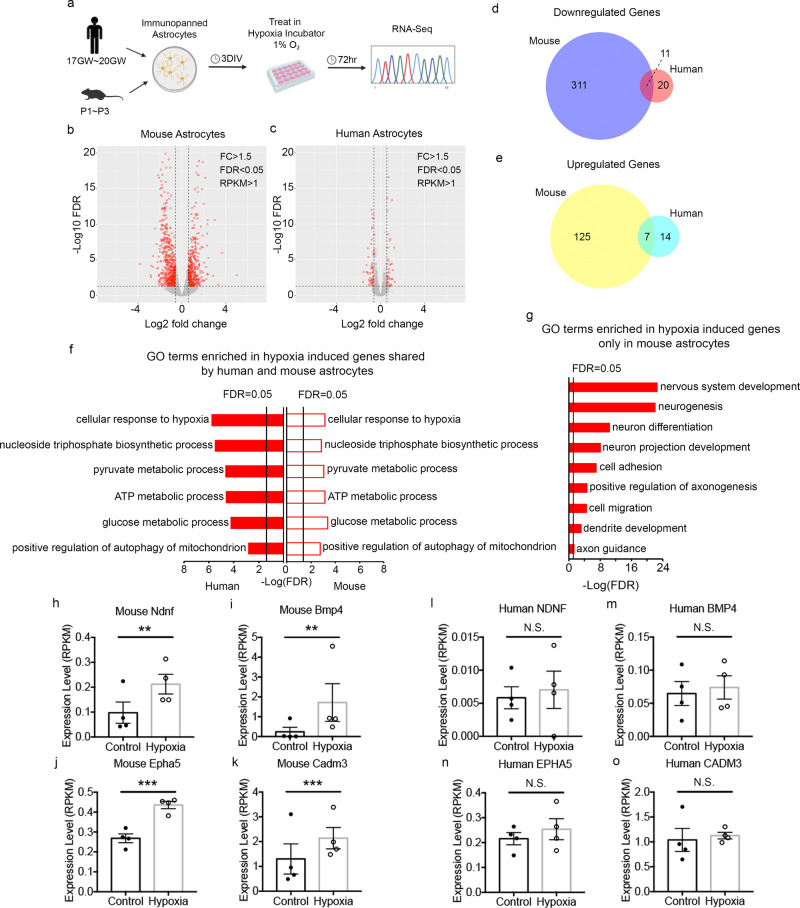
Molecular responses of human and mouse astrocytes to hypoxia. **a** Experimental design. **b**, **c** Volcano plots of genes that significantly differ between hypoxia and control conditions. Each red dot represents a significantly different gene. FDR, false discovery rate. FC, fold change. RPKM, Reads Per Kilobase of transcript, per Million mapped reads. **d**, **e** The number of significantly up or downregulated genes in hypoxia-treated human and mouse astrocytes. Genes with FDR < 0.05, fold change > 1.5, and average RPKM of control or treated groups > 1 are shown. Genes in the overlapping regions are those meeting all three criteria in both species. **f** Top shared gene ontology (GO) terms enriched in hypoxia-induced genes in human and mouse astrocytes ranked by FDR. **g** Development-associated GO terms enriched only in hypoxia-induced genes in mouse but not human astrocytes. **h**–**o** Expression of genes associated with the GO term nervous system development in control and hypoxia-treated human and mouse astrocytes. *N* = 4 litters of mice and 4 human patients. Mouse: Ndnf, *p* = 0.002; Bmp4, *p* = 0.0015; Epha5, *p* = 0.0003; Cadm3, *p* = 0.0001. Multiple comparison-adjusted *p* values were calculated by the DESeq2 package. N.S., not significant.

We exposed human and mouse astrocytes to hypoxia (Fig. 6a) and found that human and mouse astrocytes exhibited similarly high levels of cell survival and had normal healthy morphology under hypoxic and control conditions. We then performed RNA-seq of all treated and control human and mouse astrocytes. To assess transcriptional responses, we used a combination of differential expression and weighted gene co-expression network analysis (WGCNA) (Methods; Supplementary Fig. 13 and Supplementary Data 9).

When we examined the extent to which hypoxia-induced genes are shared between human and mouse astrocytes, we found that 3.4% of (11 out of 322) genes downregulated in human astrocytes were also downregulated in mouse astrocytes and 5.3% of (7 out of 132) genes upregulated in human astrocytes were also upregulated in mouse astrocytes, demonstrating partial conservation of hypoxic responses between human and mouse (Fig. 6d, e; upregulated overlap: 13.8-fold higher than expected by chance; *p* = 3.65e−07; downregulated overlap: 6.0-fold higher than expected by chance; *p* = 7.50e-07; FDR < 0.05; fold change > 1.5; average RPKM > 1; overlap: genes meeting all three criteria in both species; see also Supplementary Fig. 14). GO terms and KEGG pathway analyses revealed that genes upregulated in both human and mouse astrocytes were enriched in the GO term hypoxia response and the hypoxia inducible factor 1 (HIF1) pathway (Fig. 6f and Supplementary Data 2). Interestingly, astrocytes from both species upregulated genes involved in glycolysis and positive regulation of mitochondrial autophagy. Glycolysis provides an alternative pathway to generate energy without oxygen and autophagy of idling mitochondria may conserve resources within cells.

Despite the partial conservation of hypoxic responses between human and mouse, hypoxia induced stronger molecular changes in mouse astrocytes relative to human astrocytes in terms of the number of differentially expressed genes (454 in mouse vs. 52 in human; fold change > 1.5, FDR < 0.05, average RPKM > 1; Fig. 6b–e) and effect size (Supplementary Fig. 15). Genes upregulated by hypoxia in mouse, but not human, astrocytes were enriched in GO terms such as nervous system development, neurogenesis, neuron differentiation, and axon guidance (Fig. 6g and Supplementary Data 2) and included genes encoding the growth factor Ndnf, morphogen Bmp4, axon guidance molecule Epha5, and cell adhesion molecule Cadm3 (Fig. 6h–o and Supplementary Data 10, 11). Furthermore, we identified a module (grey60) upregulated by hypoxia in mouse astrocytes but not in human astrocytes (Supplementary Data 9 and Supplementary Fig. 13). This module is involved in development and cell adhesion, corroborating our finding that hypoxia induces a molecular program that aids neural repair specifically in mouse astrocytes. These differences may contribute to the differences in functional recovery and responses to drug candidates between human patients and mouse models of stroke.

Among the genes downregulated by hypoxia, we found that genes associated with the GO terms amino acid transmembrane transport and cellular response to nutrient levels were enriched in both human and mouse astrocytes. In contrast, the GO terms L-glutamate transmembrane transport and circadian rhythm were only enriched in downregulated genes in human astrocytes, and the terms cell cycle and electron transport chain were only enriched in downregulated genes in mouse astrocytes (Supplementary Data 2 and Supplementary Fig. 16).

### Inflammatory signals induce antigen presentation pathways in humans

Many viruses, such as human immunodeficiency virus, new world alpha viruses, and some flaviviruses (e.g., Zika virus), are capable of infecting CNS cells, inducing neuroinflammatory responses, and causing acute or long-lasting neurological deficits^47,48^. Astrocytes, along with microglia, are CNS-resident cells that modulate neuroinflammation. However, responses of human astrocytes to viral infections and their consequences to CNS homeostasis and function are poorly understood. In addition to viral infections, neuroinflammation is a core pathological component of a range of neurological conditions such as traumatic injury, stroke, neurodegeneration, and aging. tumor necrosis factor alpha (TNFα) is a major pro-inflammatory cytokine involved in neuroinflammation that induces reactivity of mouse astrocytes. Although researchers have long assumed that TNFα induces similar changes in human astrocytes, no study has compared the effect of this key pro-inflammatory cytokine on human and mouse astrocytes.

**Fig. 7:**
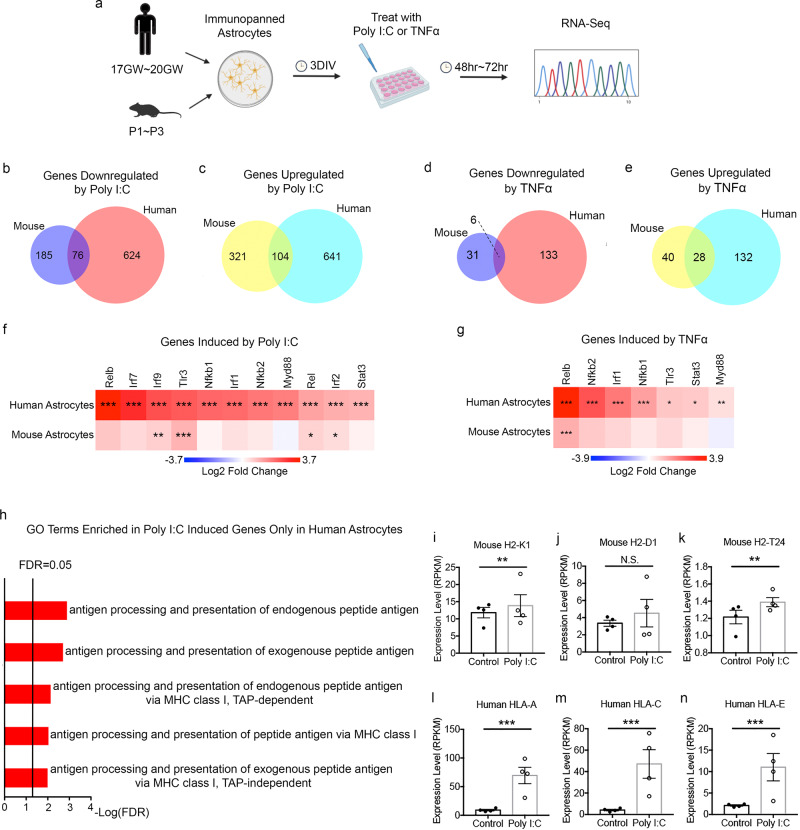
Molecular responses of human and mouse astrocytes to poly I:C and TNFα. **a** Experimental design. **b**–**e** The number of significantly up or downregulated genes in poly I:C- and TNFα-treated human and mouse astrocytes. Genes with FDR < 0.05, fold change > 1.5, and average RPKM of control or treated groups > 1 are shown. **f**, **g** Fold changes of *Tlr3, NFκB*, and interferon response pathway genes in poly I:C- and TNFα-treated human and mouse astrocytes. Asterisks represent significance determined by DESeq2. **h** Selected antigen presentation-related GO terms enriched in poly I:C-induced genes only in human astrocytes. **i**–**n** Expression of the top 3 highest-expressing MHC Class I antigen presentation genes in poly I:C-treated and control human and mouse astrocytes. *N* = 4 litters of mice and 4 human patients. Mouse: H2-K1, *p* = 0.0051; H2-T24, *p* = 0.0019. Human: HLA-A, *p* < 0.0001; HLA-C, *p* < 0.0001; HLA-E, *p* < 0.0001. Multiple comparison-adjusted *p* values were calculated by the DESeq2 package. N.S., not significant.

We exposed human and mouse astrocytes to the viral mimetic double-stranded RNA, poly I:C, or TNFα (Fig. 7a). Both human and mouse astrocytes exhibited similarly high levels of cell survival and had normal healthy morphology under treatment and control conditions. We then performed RNA-seq of all treated and control human and mouse astrocytes. In contrast to hypoxia, we found that both poly I:C and TNFα induced stronger molecular responses in human astrocytes relative to mouse astrocytes in terms of the number of differentially expressed genes (Fig. 7b-e and Supplementary Fig. 17a, b, d, e) and effect size (Supplementary Fig. 17c, f). We next examined the extent to which the poly I:C- and TNFα-induced gene changes were shared between human and mouse astrocytes. We found that a significant proportion (10.9%; 76 out of 700) of the downregulated genes in human astrocytes were also downregulated in mouse astrocytes and a significant proportion (14.0%; 104 out of 745) of upregulated genes in human astrocytes were also upregulated in mouse astrocytes under poly I:C treatment (Fig. 7b, c; upregulated: 1.7-fold higher than expected by chance; *p* = 2.79e−07; downregulated: 2.2-fold; *p* = 4.89e−12). A significant proportion (4.3%; 6 out of 139) of genes downregulated by TNFα in human astrocytes were also downregulated in mouse astrocytes and a significant proportion (17.5%; 28 out of 160) genes upregulated by TNFα in human astrocytes were also upregulated in mouse astrocytes, reflecting partial conservation between human and mouse (Fig. 7d, e; upregulated: 13.1-fold; *p* = 2.53e−25; downregulated: 5.1-fold; *p* = 9.85e−04). We also observed a significant correlation of fold change in mouse vs. human (poly I:C, 0.236. TNFα 0.192; Supplementary Fig. 14). GO term and KEGG pathway enrichment analysis of the conserved genes (Supplementary Data 2) revealed enrichment for genes involved in responses to cytokines and other organisms.

Furthermore, we examined genes induced by poly I:C or TNFα only in human astrocytes. These genes showed enriched GO terms associated with antigen processing and presentation of peptide antigen via major histocompatibility complex (MHC) class I (Fig. 7h and Supplementary Fig. 17h). The expression levels of the three highest expressing MHC Class I genes in human astrocytes (*HLA-A, HLA-C*, and *HLA-E*) and mouse astrocytes (*H2-K1, H2-D1*, and *H2-T24*) are shown in Fig. 7i–n, Supplementary Fig. 17h–m, and Supplementary Fig. 18. These genes showed modest or no increase in poly I:C- or TNFα-treated mouse astrocytes but a consistent and robust increase in human astrocytes. Genes encoding additional MHC Class I-interacting antigen processing and presenting proteins, such as *Tap1, Tap2*, and *ICAM1*, showed similarly robust increases in human astrocytes but no change in mouse astrocytes treated with poly I:C (Supplementary Data 10, 11 and Supplementary Figs. 19 and 20). Interestingly, human induced pluripotent stem cell-derived astrocytes also increased the expression of MHC Class I genes upon TNFα treatment^49^, demonstrating the value of stem cell-derived astrocytes in studying species-specific features of human astrocytes.

Among the genes downregulated by poly I:C treatment, the GO term virion assembly was enriched in both human and mouse, potentially revealing a conserved defensive response to viral infections. In addition, poly I:C treatment induced downregulation of genes associated with cell cycle and CNS development only in human astrocytes and genes associated with response to hydrogen peroxide only in mouse astrocytes. No GO term was enriched in genes downregulated by TNFα-treatment in either human or mouse, likely because of the small number of genes downregulated by TNFα in mouse astrocytes. Similar to poly I:C treatment, TNFα induced downregulation of genes associated with cell cycle and CNS development only in human astrocytes. Additionally, TNFα induced downregulation of genes associated with cell communication and glycerolipid metabolism only in mouse astrocytes (Supplementary Data 2 and Supplementary Fig. 16).

To identify coregulated gene networks changing under poly I:C or TNFα treatment, we performed WGCNA and identified a module (black) upregulated in human, but not mouse, astrocytes under both treatment conditions (Supplementary Data 9 and Supplementary Fig. 13). This module is involved in inflammatory responses to double-stranded RNA. The network analyses corroborated the finding that, at the specific dosage of poly I:C or TNFα we used, human astrocytes showed stronger inflammatory responses compared to mouse astrocytes.

Signaling pathways downstream of poly I:C treatment have been well characterized in multiple cell types of the immune system. Poly I:C binds the Toll-like receptor 3 (Tlr3), a pattern-recognition receptor located in endosomes that recognizes the danger signal. TLR3 signals through an adapter protein, Myd88, which activates the nuclear factor kappa B (NFκB) signaling pathway (e.g., Rel, Relb, Nfkb1, Nfkb2). NFκB activation and nuclear translocation, in turn, activates the interferon signaling pathway (interferon responsive genes include *Irf1, Irf2, Ifr7*, and *Irf9*). The NFκB signaling pathway cross-talks with Stat3, the phosphorylation of which is involved in astrocyte reactivity. We found that all of the above-mentioned molecules are strongly upregulated after poly I:C treatment in human astrocytes but showed modest or no upregulation in mouse astrocytes (Fig. 7f and Supplementary Fig. 21a). We found a similar pattern of stronger activation of these genes in TNFα-treated human astrocytes compared to mouse astrocytes (Fig. 7g and Supplementary Fig. 21b).

To assess whether cell death affected our transcriptome analyses, we examined cell survival in acutely purified and cultured human and mouse astrocytes. By trypan blue staining of dead cells, we found close to 100% survival of acutely purified astrocytes from both humans and mice. Astrocyte cultures always have a small proportion of dead cells, but we did not observe differences in cell survival between human and mouse astrocytes (Supplementary Fig. 22). To determine whether cell death may have affected our RNA-seq analyses, we examined the expression of cell death-associated genes^50^ in our RNA-seq data. We found low or no expression of these genes in all conditions tested (acutely purified, serum-selected culture, serum-free culture, xenograft, host, hypoxia-, poly I:C-, TNFα-treated, and untreated control astrocytes from both human and mouse; Supplementary Data 12). None of the cell death-associated genes were differentially expressed in any treatments we performed. Therefore, cell death is unlikely to compromise RNA-seq analyses under the conditions we tested.

We next examined whether astrocytes treated with various challenges secrete signals that differentially affect neuronal attributes. We treated cultured human and mouse astrocytes with hypoxia and TNFα, collected astrocyte conditioned medium (ACM), and applied the ACM to mouse cortical neurons. We did not observe differences in neuronal survival, process outgrowth, or NFκB activation between any groups of ACM-treated neurons. We further assessed whether neurons exhibited molecular changes in response to hypoxia or TNFα-treated human and mouse ACM by performing RNA-seq of the treated neurons. We found that neurons treated with hypoxia-mouse ACM showed downregulation of non-coding RNAs such as *Rn7sk* and *Gm24187*. Neurons treated with hypoxia-human ACM showed downregulation of non-coding RNAs such as *Rn7sk, Bc1*, and *Gm24187* (Supplementary Data 13). No protein-coding genes exhibited significant gene expression differences between ACM treatment groups. TNFα-treated human and mouse ACM did not induce significant gene expression changes in neurons. In these experiments, we did not test contact-dependent astrocyte-neuron interactions, which may be interesting to investigate in future studies.

### Poly I:C and TNFα induce common transcriptional responses

We next investigated whether different types of perturbations induce a shared core astrocyte reactivity program vs. distinct programs specific to each perturbation. We found that, in both species, very few genes were induced by all three stimuli (i.e., hypoxia, poly I:C, and TNFα; Supplementary Fig. 23). Poly I:C and TNFα induced many common gene changes, but these genes differed greatly from the hypoxia-induced genes, which was corroborated by WGCNA results (Supplementary Fig. 13).

### Comparison of treatment-induced changes with neurological diseases

**Fig. 8:**
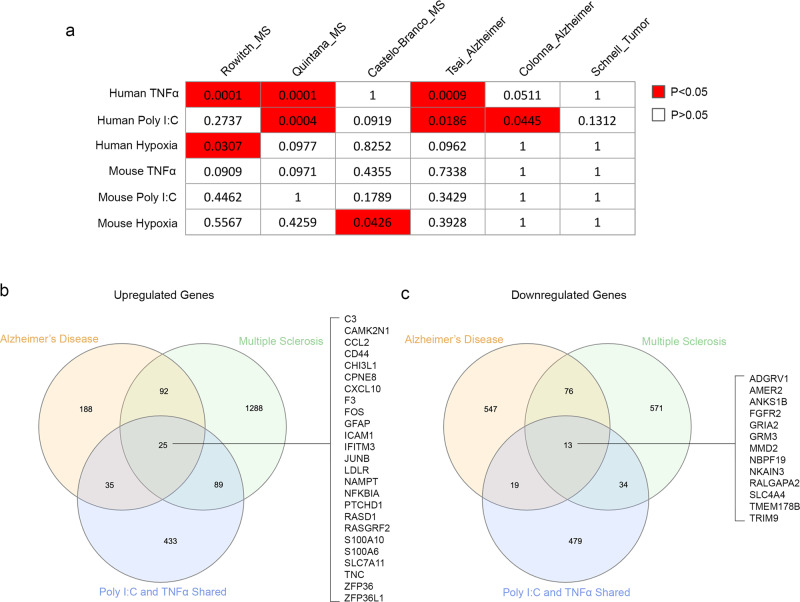
Core inflammatory astrocyte genes shared in Alzheimer’s disease, multiple sclerosis, and poly I:C and TNFα treatments in humans. We analyzed single-cell RNA-seq data from Alzheimer’s disease patients, multiple sclerosis patients, and healthy controls. We compared the differentially regulated genes in astrocytes in these diseases with the hypoxia-, poly I:C-, and TNFα-induced genes we identified in cultures of human and mouse astrocytes. **a** Significant concordant gene expression changes are present in disease conditions in vivo and in astrocyte treatments in vitro. To test whether treatment A and disease B exhibited concordant gene expression changes, we counted the number of genes in the following four categories: (1) upregulated in treatment A and upregulated in disease B; (2) upregulated in treatment A and downregulated in disease B; (3) downregulated in treatment A and downregulated in disease B; and (4) downregulated in treatment A and upregulated in disease B. We used the number of genes in each of the four categories in a contingency table and used two-sided Fisher’s exact test to detect significant concordance. MS, multiple sclerosis. **b**, **c** We found 38 core inflammatory astrocyte genes shared by Alzheimer’s disease, multiple sclerosis, and poly I:C and TNFα treatment in humans, 25 of which are upregulated (**b**) and 13 are downregulated (**c**) in diseases and treatments.

To compare hypoxia-, poly I:C-, and TNFα-induced changes of cultured human and mouse astrocytes with neurological disease-associated changes in human patients and mouse models in vivo, we analyzed single-cell RNA-seq datasets of Alzheimer’s disease^51,52^, multiple sclerosis^53–55^, and healthy control patients and bulk RNA-seq data of glioblastoma-associated astrocytes^56^ (see Methods for details). Interestingly, we found that poly I:C- and TNFα-treated human astrocytes exhibit shared gene expression changes with astrocytes from both Alzheimer’s disease and multiple sclerosis patients (Fig. 8). We found that 38 genes showed consistent changes in astrocytes in Alzheimer’s disease, multiple sclerosis, poly I:C treatment, and TNFα treatment (25 genes were upregulated and 13 genes were downregulated by diseases and treatments; Fig. 8b, c). The upregulated genes include those involved in interferon (*IFITM3*), NFκB (*NFKBIA*), and cytokine (*CCL2, CXCL10*) signaling, immediately early genes (*FOS, JUNB*), and calcium signaling modulators (*S100A6, S100A10*). The downregulated genes include one encoding a protein that interacts with amyloid-β precursor protein (*ANKS1B*) and two encoding glutamate receptors (*GRIA2, GRM3*), suggesting changes in astrocytic responses to synaptic glutamate release in multiple neurological disorders. Because both poly I:C and TNFα can induce inflammatory changes, and there are inflammatory changes in Alzheimer’s disease and multiple sclerosis, these 38 genes are likely a core group of signature inflammatory astrocyte genes in humans. Using these genes as markers may facilitate the identification of inflammatory-reactive astrocytes in multiple diseases in the future. In contrast to poly I:C- and TNFα-treated human astrocytes, we did not detect any significant correlation between gene expression changes of poly I:C- or TNFα-treated mouse astrocytes and Alzheimer’s disease or multiple sclerosis patients, highlighting species-dependent gene signatures in astrocyte reactivity (Fig. 8a).

We detected a weak correlation of gene expression changes in hypoxia-treated human astrocytes with a small subset of disease datasets. Therefore, hypoxia-induced changes are likely distinct from changes of astrocytes in Alzheimer’s disease or multiple sclerosis. We did not observe any correlation of glioblastoma-associated astrocyte gene expression changes with any treatment-induced changes in human or mouse astrocytes.

We next compared our identified treatment-induced gene expression changes with gene expression changes in the astrocytes of two mouse models of neurological disorders, bacterial endotoxin lipopolysaccharide-induced inflammation and ischemic stroke^27^, also referred to as A1 and A2 astrocytes in the literature^57,58^. We did not observe any significant correlation between any of the treatments and A1 or A2-specific gene expression changes (Supplementary Fig. 24).

### Astrocyte heterogeneity and hypoxia-, poly I:C-, and TNFα-induced changes

To examine astrocyte heterogeneity, we assessed NFκB activation at the single-cell level in poly I:C-treated human and mouse astrocytes. We performed immunostaining with an antibody against the NFκB component p65, which is localized at the nuclei when NFκB signaling is activated^59^. Upon poly I:C treatment, a larger subpopulation of human astrocytes compared to mouse astrocytes exhibited NFκB activation (Supplementary Fig. 25; mouse: 0.9±0.4% in control, 2.9±0.5% in poly I:C-treated; human: 1.0±0.2% in control, 13.9±1.7% in poly I:C-treated; mouse poly I:C-treated vs. human poly I:C-treated: *p* < 0.001), revealing species-dependent properties of astrocyte subpopulation dynamics.

To assess whether hypoxia, poly I:C, or TNFα may induce changes of previously characterized astrocyte subpopulations, we compared our treatment-induced gene expression changes with astrocyte subpopulation markers from single-cell RNA-seq studies^51,54,60,61^. We found significant concordance between poly I:C- and TNFα-treated human astrocytes with astrocyte cluster 3 reported by Tsai and colleagues^51^ and anti-correlated gene expression between poly I:C- and TNFα-treated human astrocytes with astrocyte cluster 1 reported by Schwartz and colleagues^61^ (Supplementary Fig. 26). Tsai et al. astrocyte cluster 3 expresses well-known reactive astrocyte markers, such as *glial fibrillary acidic protein (GFAP), IFITM3*, and *CD44*. These observations suggest that poly I:C and TNFα treatment increase the subpopulation of astrocytes with reactive characteristics, which could result from dynamic gene expression changes and/or selective proliferation/depletion of subpopulations. In contrast to poly I:C and TNFα treatment, we did not observe concordant gene expression changes between hypoxia treatment and any reported astrocyte subpopulations. We did not detect concordant gene expression between any of our treatments with any astrocyte subpopulations reported by Regev and colleagues^60^.

## Discussion

In this study, we evaluated the conservation and divergence of astrocytic responses to disease-relevant perturbations between human and mouse. We used methods for isolating, culturing, and stimulating resting/homeostatic astrocytes from developmentally matched human and mouse astrocytes and applied equivalent, controlled experimental paradigms for direct comparison. We identified several important differences between both resting and reactive human and mouse astrocytes: (1) The rates of mitochondrial resting state respiration differed between mouse and human astrocytes. (2) Human astrocytes were more susceptible to oxidative stress than mouse astrocytes, potentially contributing to the observed differences in neurodegeneration between mouse models and human patients. (3) Hypoxia induced a pro-growth molecular program in mouse but not human astrocytes, potentially underlying the greater functional recovery that occurs in mouse models of ischemic stroke compared to human stroke patients. (4) Poly I:C and TNFα induced antigen-presenting genes in human but not mouse astrocytes.

### Utilizing knowledge on the conservation and divergence of human and mouse astrocytes for translational research

#### Identifying conserved and divergent cellular processes

We found extensive conservation in gene expression levels between human and mouse astrocytes in some cellular processes and divergence in others. For example, genes with similar expression levels in human and mouse astrocytes include those involved in mRNA metabolic processes, intracellular transport, and glial cell differentiation, whereas mitochondrial metabolism and cytokine signaling genes are divergent across species. Therefore, findings on mRNA metabolic processes, intracellular transport, and glial cell differentiation using mouse models may be readily translatable to humans. By contrast, more caution must be taken before extrapolating mitochondrial and cytokine findings from mouse models to human patients.

#### “Humanizing” mouse models of diseases

Mouse models of neurodegeneration often have less severe defects compared to human patients^1–4^. Oxidative stress is a critical pathological process in neurodegeneration. Our finding of greater resilience of mouse astrocytes to oxidative stress compared to human astrocytes suggests that reducing detoxification activities in mouse models of neurodegeneration (for example, using Catalase heterozygous or knockdown mice) may improve the resemblance of these models to human patients.

#### Improving neural repair in humans by investigating repair mechanisms in mice

Mouse models of ischemic stroke typically exhibit spontaneous functional recovery^5,62^, whereas human patients often have limited functional recovery and permanent disabilities. We showed that hypoxia induced HIF1 pathway activation, increases in glycolysis, and stimulation of autophagy in both species. However, hypoxia induced a pro-growth molecular program specifically in mouse astrocytes; human astrocytes were able to sense an oxygen shortage and make adaptive changes but stopped short of activating the pro-growth program. Investigating how signal transduction occurs in mouse astrocytes that links hypoxia to neuronal growth genes may lead to therapies that activate the pro-neuronal growth program in human astrocytes.

### Species differences in energy metabolism in astrocytes and other cell types

A few genes are associated with the expansion of the cerebral cortex in human evolution^63–68^; one such gene encodes a protein targeted to mitochondria, implicating metabolic changes in human brain evolution^69^. It is unclear whether the species differences in metabolism that we identified are specific to astrocytes, but we found consistent species-dependent expression of genes associated with reactive oxygen species detoxification in multiple cell types in the brain. Other studies have reported transcriptome and developmental differences between human and mouse brains^29,70–72^. However, very few studies have compared the respiration rates of human and mouse cells. One study that compared the metabolism of human and mouse muscle cells reported mixed results^73^. At the organism level, our results are consistent with the observation that smaller mammals typically have higher metabolic rates per unit body weight than larger mammals^74^.

Mitochondrial and energy metabolism changes are important in the pathogenesis of many neurological disorders. For example, many genes associated with Parkinson’s disease risk are involved in mitochondrial function^75^, a large set of genes involved in metabolism are induced after traumatic brain injury^76^, and impairment of glycolysis-derived metabolites in astrocytes contributes to cognitive deficits in Alzheimer’s disease^77^. Previous studies have not directly compared the mitochondrial function and energy metabolism between human and mouse for any cell type of the central nervous system, to the best of our knowledge. Our discovery of mitochondrial and energy metabolism differences between human and mouse cells should be taken into consideration in translational research.

### Potential species-specific interactions between astrocytes and neurons

Astrocytes are important for the development and function of neurons. Previous studies have shown that transplantation of human astrocytes into mouse brains affects neuronal function and learning and memory^30^. In this study, we compared the impact of secreted signals from human and mouse astrocytes on neurons and did not observe significant species differences. It is likely that contact-dependent interactions between astrocytes and neurons exhibit species-specific attributes. It is also possible that the concentration of astrocyte-conditioned media we used is not high enough to induce detectable species-dependent effects.

### Potential limitations of the study

All in vitro experiments in this study were performed using developing human and mouse astrocytes. Therefore, we do not recommend extrapolation of our conclusions to adult and/or aging contexts without further investigation. Although it is important to directly compare adult human and mouse astrocytes, it is challenging to obtain large numbers of fresh healthy brain tissue donations from adults to characterize astrocytic responses to disease-relevant stimuli with sufficient statistical power. Nevertheless, we performed RNA-seq of astrocytes purified from healthy brain tissue donated from adults (Supplementary Data 8)^26^. Analysis of adult astrocyte RNA-seq data showed human-mouse divergent pathways consistent with our in vitro findings, suggesting that the potential species differences are similar between developing and adult stages (Fig. 5a, b).

## Methods

### Lead contact and materials availability

Further information and requests for resources and reagents should be directed to and will be fulfilled by the Lead Contact, Ye Zhang (yezhang@ucla.edu). This study did not generate new unique reagents.

### Experimental animals

All animal experimental procedures were approved by the Chancellor’s Animal Research Committee at the University of California, Los Angeles (UCLA) and were conducted in compliance with national and state laws and policies. The research protocol for the transplantation of human cells into host mouse brains to create chimera model was approved by the Chancellor’s Animal Research Committee at UCLA and were conducted in compliance with national and state laws and policies. We used C57BL6 mice group-housed in standard cages (2–3 per cage). Rooms were maintained on a 12-h light/dark cycle at 20–26 °C, 30–70% humidity. Euthanasia and preparation of primary cultures of astrocytes were performed during the light cycle. For each astrocyte culture batch, 8–10 mixed-sex pups at P1–3 from 1 to 2 L were combined.

### Human tissue samples

Fetal human brain tissue without identifiable personal information was obtained following elective pregnancy termination with exemption determination from the UCLA Office of the Human Research Protection Program. All donors have provided informed consent. The consent forms indicate that the donation is voluntary, refusal to donate tissue will not affect the donor’s medical care or their relationship with their physicians, the donated material will be used for purposes of education, research, or for the advancement of medical science, and that there will be no payment to the donor. The next of keen were not involved in providing informed consent. Samples from patients with genetic disorders such as Down’s syndrome were excluded from the study when known. Gestational week 17–20 brain tissue was immersed in 4 °C Dulbecco’s phosphate buffered saline (PBS, Gibco, 14040182) and transferred to the lab for tissue dissociation. In cases with largely intact brain tissue, we used whole cerebral cortex for astrocyte purification. In cases with fragmented tissue, we used fragments most likely to be cerebral cortex (typically large thin sheets). We included both female and male brain tissue. Sample sizes are noted in figure legends for each experiment. Results on astrocytes from children and adults was obtained by analyzing a previously published dataset^26^.

### Primary cell culture

Primary astrocyte cultures from humans and mice were generated by immunopanning and were maintained in a humidified 37°C incubator with 10% CO_2_ (see method details below). Cells from both females and males were used.

### Immunopanning purification of astrocytes

To examine responses of human and mouse astrocytes to stressors, we purified human and mouse astrocytes from developmentally equivalent stages, to the best of our knowledge. In humans, astrocytogenesis starts during the second trimester and continues through the third trimester^78–80^. Human astrocytes reach maturity roughly around one year of age as determined by gene expression^70–72^, although their physiological and functional maturation timeline is unclear. In mice, astrocytogenesis starts at the perinatal period (embryonic day 17.5 [E17.5] of a 19-day gestation) and peaks between P0 and P14^81^. Mouse astrocytes reach maturity roughly around one month of age as determined by morphology and gene expression^82^. A single-cell RNA-seq study of human and mouse brains found that molecular features of gestational week 16–20 human brains are similar to P0-P5 mouse brains^83^. Therefore, we purified astrocytes from gestational week 17–20 human brains and P1–3 mouse brains. Within those age ranges, we did not observe age-dependent differences in any of the assays we tested.

We started astrocyte purification experiments using human and mouse brain tissue within similar postmortem intervals. For human samples, we received tissue within 30 min to 1 h postmortem. We then performed a very simple dissection procedure that takes <3 min. For mouse samples, we combined one to two litters of 8–10 mice to get enough cells for each experiment. We combined both male and female mice for all experiments, although sex may not have been equally represented in each litter of mice. Cerebral cortex dissection from all the mice typically took ~45 min before we started the astrocyte purification experiments. We purified human and mouse astrocytes according to a previously published immunopanning protocol^26,28,41^. Briefly, we coated three 150 mm-diameter petri dishes first with species-specific secondary antibodies and then with an antibody against CD45 (BD550539, both human and mouse, 10 μl antibody in 12 ml buffer per panning plate), a hybridoma supernatant against the O4 antigen (mouse, 4 ml hybridoma supernatant in 8 ml buffer per panning plate) or an antibody against CD90 (BD550402, human, 20 μl antibody in 12 ml buffer per panning plate), and an antibody against HepaCAM (R&D Systems, MAB4108, 10 μl antibody in 12 ml buffer per panning plate), respectively. We dissected cerebral cortices from human and mouse in PBS and removed meninges. We then dissociated the tissue with 6 units/ml papain at 34.5°C for 45 min. We mechanically triturated the tissue with 5 ml serological pipets in the presence of a trypsin inhibitor solution. We then depleted microglia/macrophages, oligodendrocyte precursor cells, and neurons from the single-cell suspension by incubating the suspension sequentially on the CD45, O4 (for mouse), or CD90 (for human) antibody-coated petri dishes. We incubated the single-cell suspension on the HepaCAM antibody-coated petri dish. After washing away nonadherent cells with PBS, we lifted astrocytes bound to the HepaCAM antibody-coated petri dish using trypsin and plated them on poly-D-lysine-coated plastic coverslips in a serum-free medium containing Dulbecco’s modified Eagle’s medium (DMEM) (Life Technologies, 11960069), Neurobasal (Life Technologies, 21103049), sodium pyruvate (Life Technologies 11360070), glutamine (Life Technologies, 25030081), N-acetyl cysteine (Sigma, A8199), and heparin-binding EGF-like growth factor (Sigma, E4643). For most of the H_2_O_2_, TNFα, hypoxia, and poly I:C treatment experiments, with exceptions detailed below, astrocytes were plated on 24-well culture plates at 75–100k per well. Human and mouse astrocyte cultures had similar final densities for every type of experiment. For high-density cultures for H_2_O_2_ treatment, 30k astrocytes were plated in a 50 μl droplet in the middle of pre-dried poly-D-lysine-coated plastic coverslips on 24-well plates. After allowing the cultures to settle for 20 min at 37°C, additional media were added. For Seahorse Respiration Assays, astrocytes were plated at 100–250k/well in Agilent Seahorse 96-well cell culture microplates (cat#101085-004). For TMRE/MTG imaging, astrocytes were plated at 25–50k/well on dark-walled flat-bottom 96-well assay plates (Corning, cat#3603). For poly I:C treatment, astrocytes were plated directly on poly-D-lysine-coated 24-well culture plates (Fisher, cat#08-772-1) without coverslips because poly I:C addition often causes cell to float away from the coverslips. To purify xenografted human astrocytes and host mouse astrocytes from adult host mouse brains, we dissociated whole brains using 20 units/ml papain, depleted microglia/macrophages, oligodendrocytes, and oligodendrocyte precursor cells with anti-CD45 antibody-, GalC hybridoma supernatant-, and O4 hybridoma supernatant-coated plates, respectively. Three consecutive plates with the same antibody were used for depletion of each cell type. We then collected astrocytes with anti-HepaCAM antibody-coated plates. The general procedures we used for the purification of human and mouse astrocytes are based on a previously developed method for purifying rat astrocytes^41^, although we used different versions of antibodies for the isolation of cells from different species.

### Serum-selection purification of astrocytes

Human brain tissue was dissociated into single-cell suspensions as described above and plated on poly-D-lysine-coated 25 cm^2^ culture flasks (VWR, cat#10861-672) in DMEM (Gibco, cat#11960044) with 10% fetal bovine serum (Gibco, cat#16140071) and 2 mM glutamine. After 4-6 days, we vigorously shook off the cells in the top layer (neurons and other glia) and left the astrocytes on the bottom layer. We then harvested astrocytes for RNA-seq.

### RNA-seq

We purified total RNA using the miRNeasy Mini kit (Qiagen, cat#217004) and analyzed RNA concentration and integrity with TapeStation (Agilent) and Qubit. All samples showed RNA integrity numbers higher than 8.4. We then generated cDNA using the Nugen Ovation V2 kit (Nugen), fragmented the cDNA using a Covaris sonicator, and generated sequencing libraries using the Next Ultra RNA Library Prep kit (New England Biolabs) with 9–10 cycles of PCR amplification. We sequenced the libraries with Illumina HiSeq 4,000 and NovaSeq sequencers and obtained 16.3 ± 5.7 million (mean ± standard deviation) 50 bp and 100 bp single-endreads per sample.

### RNA-seq data analysis

We mapped sequencing reads to human genome hg38 and mouse genome MM10 using the STAR package and HTSEQ to obtain raw counts. We then used the EdgeR-Limma-Voom packages in R to obtain RPKM values. We calculated differential gene expression with the DESeq2 package. Statistical significance of the overlap between two groups of genes was determined using http://nemates.org/MA/progs/overlap_stats.cgi. Significance of the difference between two correlation coefficients was calculated using http://vassarstats.net/rdiff.html?.

### Comparison of transcriptomes of acutely purified astrocytes

We mapped RNA-seq data from our previously obtained acutely purified human and mouse astrocyte datasets^26,28^ as described above. The ages of the samples is described in Supplementary Data 8. We calculated percentile rankings of RPKM values of each gene in each human and mouse astrocyte sample. We excluded genes with maximal percentile rankings across all samples < 0.33, as these genes are not expressed or very lowly expressed in all samples. We then performed Welch’s T-test between human and mouse samples and multiple-comparison post-hoc adjustment using the FDR method. Genes with FDR values < 0.05 and human-mouse percentile ranking differences > 0.4 were used for GO and cellular component analyses using string-db.org. Test gene lists were compared to background gene lists including all genes expressed at RPKM > 0.05 in astrocytes.

### Comparison of human data to transcriptome data of 14 mouse strains

To test whether the human-mouse astrocytic gene expression differences are specific to the C57/BL6 strain we used, we compared our data to an RNA-seq study of mouse hippocampus from multiple strains (data are available from 15 strains)^84^. Notably, the Neuner study^84^ and our study differ in technical details. Therefore, to avoid the impact of technical batch effects in the comparison of our human data to mouse data from 15 different strains, we took advantage of the fact that both studies performed RNA-seq of the C57/BL6 mouse strain. We divided the expression of each gene from our human samples by the average expression in our C57/BL6 mouse samples to obtain normalized expression of each gene from each sample. Similarly, we divided the expression of each gene in each of the 14 strains (other than C57/BL6) from the Neuner study by the average expression in the C57/BL6 strain determined by the Neuner study to obtain normalized expression of each gene from each sample in the Neuner study. We then compared normalized expression in our study to normalized expression in the Neuner study. We avoided direct comparison of expression levels (e.g., RPKM/ fragments per kilobase per million mapped reads (FPKM)/transcripts per million (TPM)) across studies because it would be complicated by technical batch effects.

### Comparison of single-cell RNA-seq data of human and mouse astrocytes

To validate our observed human mouse astrocyte gene expression differences, we utilized single-cell expression data derived from human and mouse cortex^29^. For human and mouse, respectively, we utilized the available trimmed-mean and median expression TPM values for all genes in each of their identified cell-type clusters. To calculate the human-mouse expression fold-change difference, we calculated the mean expression of astrocyte clusters and compared the mean expression between species. This fold-change species difference was compared to the fold-change species difference calculated using acutely purified astrocytes from both human and mouse.

### WGCNA

Expression values from human and mouse were merged into a single expression matrix using only one-to-one human-mouse orthologues. Genes were retained if they had > 20% non-zero values and were subsequently log2 (+0.001) transformed. We combined all conditions in the analyses. We removed expression variation unrelated to the effect of treatment using the linear regression model “expr ~ (1 | replicate)”. This maintained differences within each replicate pair, capturing the effect of a treatment, but regressed out differences between replicate pairs such as basal species differences or technical differences such as sequencing batch. Network analysis was performed through WGCNA using biweight midcorrelation (bicor) to reduce sensitivity to outliers. A soft threshold power of 18 was chosen to achieve scale-free topology (*r*^2^ > 0.8). The topological overlap matrix was hierarchically clustered and modules were defined using a minimum module size of 50 and deepSplit cut of 2. Module-trait correlations were used to assess whether a module was significantly associated with a particular treatment in a particular species.

### H_2_O_2_ treatment

We treated human and mouse astrocytes cultured in 24-well plates with 100–500 μM H_2_O_2_ (Sigma, cat#95321-100 ML) and performed the cell survival assay, Seahorse respiration assay, and mitochondrial membrane potential assay described below.

### Cell survival assay

We incubated human and mouse astrocytes with the live cell dye calcein-AM and the dead cell dye ethidium homodimer using the LIVE/DEAD^TM^ Viability Kit (Invitrogen, cat#L3224) for 10 min at room temperature protected from light and imaged the cells with an Evos FL Auto 2 inverted fluorescence microscope (Invitrogen) with a 10× lens.

### Seahorse respiration assay

We cultured human and mouse astrocytes with the media detailed above and changed it to Seahorse assay medium with 10 mM glucose, 2 mM glutamine, 1 mM pyruvate, and 5 mM HEPES on the day of the Seahorse respiration assay. We used an Agilent Seahorse XFe96 Analyzer to measure oxygen concentration and extracellular pH changes. We first measured basal oxygen consumption rates in unperturbed conditions. We then added oligomycin to inhibit ATP-synthase (mitochondrial complex IV). The differences between the basal and oligomycin conditions reflect the amount of oxygen consumption used for ATP production. We next added carbonyl cyanide-4 (trifluoromethoxy) phenylhydrazone (FCCP), an uncoupling agent that collapses the proton gradient and disrupts the mitochondrial membrane potential. As a result, electron flow through the electron transport chain is uninhibited, and oxygen consumption by complex IV reaches the maximum amount. Lastly, we added antimycin A to block complex III and shut down mitochondrial respiration. In the presence of antimycin A, the measured respiration rate represents non-mitochondrial respiration, with major contributions from peroxisomes. We took measurements every 5 min for 3–4 data points per condition. We sequentially added 2 μM oligomycin, 0.5 μM and 0.9 μM FCCP, and 2 μM antimycin A. After taking measurements, we stained cells with DAPI and counted the number of cells in each sample. Results were then normalized by cell number. We used the Agilent Seahorse Wave software to analyze Seahorse assay data.

### Mitochondrial membrane potential assay

We loaded cultured human and mouse astrocytes with 14 nM TMRE, 200 nM MTG, and 1 μg/ml Hoechst for 45 min, treated the cells with 100 μM H_2_O_2_, and then measured fluorescence at 1 and 3 h after H_2_O_2_ treatment. After staining, the cells were washed three times with culture medium containing 14 nM TMRE to remove extra MTG and Hoechst dyes. TMRE and MTG fluorescence were imaged with an Operetta High-Content Imaging System (PerkinElmer). Fluorescence intensity after H_2_O_2_ treatment was normalized to untreated control.

### Hypoxia treatment

We first cultured immunopanned human and mouse astrocytes at atmospheric oxygen concentrations for three days. We then cultured them at 1% oxygen for three days. Control cells were cultured at atmospheric oxygen concentrations for 6 days. We then harvested RNA for RNA-seq.

### Poly I:C treatment

We cultured immunopanned human and mouse astrocytes for three days. We then added 200 μg/ml poly I:C (Sigma, cat#P1530-25MG) to the culture medium and cultured the cells for an additional three days. We then harvested RNA for RNA-seq.

### TNFα treatment

We treated human and mouse astrocytes cultured for 3 days with 30 ng/ml TNFα for 48 h and harvested the cells for RNA-seq. We treated mouse astrocytes with TNFα from human (Cell Signaling Technology, 8902SF) and mouse (Cell Signaling Technology, 5178SF) sources and sequenced them in separate experiments. A similar number of genes were induced in mouse astrocytes by TNFα from human and mouse sources. We used cells treated with human TNFα for subsequent analyses.

### Transplantation of human astrocytes into host mouse brains

We transplanted human astrocytes into host mouse brains according to published protocols^30–32^. Briefly, we purified human astrocytes as described above under the serum-selection purification of astrocytes section. We then injected 100,000 cells per μl, 1 μl per injection, and 4 injections per mouse at age P2–11. We used Rag2 immunodeficient mice to avoid graft rejection. The mice were maintained in autoclaved cages with autoclaved food and water in a pathogen-free facility.

### Mapping xenograft reads to combined human-mouse reference genome

RNA-seq data were assessed for quality parameters using FastQC (http://www.bioinformatics.babraham.ac.uk/projects/fastqc) and then trimmed with Trim_galore (https://www.bioinformatics.babraham.ac.uk/projects/trim_galore/). The RNA-seq reads were then mapped to an in silico combined human-mouse reference genome. Briefly, reference genome and gene annotation files of human (hg38) and mouse (mm10) were downloaded from GENCODE. Human chromosomes were tagged as “chr” and mouse chromosomes were renamed as “m.chr”. The two fasta files for human and mouse were then concatenated and indexed using STAR aligner, allowing only one top-scored locus to be mapped if multiple mappings occur. Benchmarking results showed low false-alignment rates in both pure human (0.74%) and pure mouse RNA-seq (2.74%). After alignment, bam files were separated based on the “chr” (human) and “m.chr” (mouse) labels, followed by read counting using Rsubread to obtain the corresponding count matrix.

### Non-supervised hierarchical clustering

We performed clustering in R using the hclust() function.

### Comparison of host vs. naïve mouse astrocytes

We compared the transcriptomes of host mouse astrocytes (average age 8 months) to naïve mouse astrocytes of a similar age (7 or 9 months) from our earlier study^26^. The percentile ranking of each gene was calculated based on RPKM and differences in percentile were tested by the t-test with post-hoc Bonferroni correction for multiple comparisons.

### Comparison with human patients and mouse models of disease

To analyze Alzheimer’s disease- and multiple sclerosis-associated changes, we used single-cell RNA-seq datasets^51,52,54,55^. For glioblastoma-associated changes, we used a bulk RNA-seq dataset from purified astrocytes^56^. For single-cell RNA-seq datasets, we only used differentially expressed genes in the astrocyte clusters. The comparison of our treatment-induced signature from bulk RNA-seq data and published disease signature from single-cell RNA-seq data must be conducted carefully to avoid technical bias. There are major differences in the sample sources, sample collection methods, and sequencing parameters between datasets. Notably, single-cell and bulk RNA-seq data differ substantially in dynamic range. Therefore, direct comparison of counts or RPKM/FPKM/TPM between single-cell and bulk RNA-seq datasets may be problematic. To perform comparisons with minimal complications from technical variants, we compared the overlap of differentially expressed gene lists from our treatment study and published disease studies. If treatment A and disease B induce similar gene signature changes, we expect to find significantly more genes changed in concordant directions in A and B compared to genes changed in opposite directions in A and B. If a treatment and a disease do not induce similar gene signature changes, we expect to find similar numbers of genes changing in concordant vs. opposite directions in these two conditions as predicted by chance. To test whether treatment A and disease B exhibited concordant gene expression changes, we counted the number of genes in the following four categories: (1) upregulated in treatment A and upregulated in disease B; (2) upregulated in treatment A and downregulated in disease B; (3) downregulated in treatment A and downregulated in disease B; and (4) downregulated in treatment A and upregulated in disease B. We used the number of genes in each of the four categories in a contingency table and used Fisher’s exact test to detect significant concordance. We used the lists of genes differentially expressed by astrocyte clusters between disease and control patients from the published disease studies, which used the statistical tests and parameters detailed in these publications^51,52,54,55^. Specifically, the following gene lists were used for this analysis: Mathys et al.^51^, Supplementary Data 2, astrocyte cluster, no pathology vs. pathology; Zhou et al.^52^, Supplementary Data 4, DEG tab, astrocyte cluster, Alzheimer’s disease vs. control; Schirmer et al.^53^, Supplementary Data 6, astrocyte cluster, multiple sclerosis vs. control; Wheeler et al.^54^, Supplementary Data 10 and 12, astrocyte cluster, multiple sclerosis vs. control; and Heiland et al.^56^, Fig. 1b, tumor vs. control.

### Comparison with astrocyte subpopulation markers

We analyzed four previously published single-cell RNA-seq datasets from humans with subclusters of astrocytes^51^, using a similar methodology as described above for comparison with disease datasets. When the number of genes was <1000, we used Fisher’s exact test; when the number of genes was equal to or more than 1000, we used Chi-square test. We next performed Bonferroni correction for multiple comparisons to identify concordant gene expression between each treatment and each astrocyte subcluster. The following astrocyte subcluster differentially expressed gene lists were used in this analysis: Mathys et al.^51^, Supplementary Data 6; Wheeler et al.^54^, Supplementary Data 8; Habib et al.^60^, Supplementary Data 8; and Habib et al.^61^, Supplementary Data 2.

### ACM treatment of neurons

We plated primary human and mouse astrocytes purified by immunopanning as described above in high-density cultures on 6-well plates. To obtain TNFα-treated ACM, we added 30 ng/ml TNFα to the astrocytes at 3 div and harvested ACM at 6 div. To obtain hypoxia-treated ACM, we cultured the astrocytes in atmospheric (21%) oxygen for 3 days and moved the cultures to an incubator with 1% oxygen for 3 days and collected ACM at the end of the treatment. We also collected untreated control ACM. We concentrated ACM with centrifuge tubes with 3 kilodalton filters (Thermo Scientific, 88525) and spun them at 6000–8000 g for 3–4 h at 4 °C and stored the ACM in single-use aliquots at −80°C. We generated primary cortical neuron cultures from E17 mice, added ACM (150 μg total protein/ml) at 0 div and then added AraC (5 μM) at 1 div to eliminate contaminating astrocytes. We harvested the neurons at 6 div, collected RNA, generated sequencing libraries, and performed sequencing and data analyses as described above.

### Principal component analysis

The R package ggplot2 was used for principal component analysis with logRPKM as the input using all default settings.

### Immunohistochemistry and immunocytochemistry

Mice were anesthetized with isoflurane and transcardially perfused with PBS followed by 4% paraformaldehyde (PFA). Brains were removed and further fixed in 4% PFA at 4°C overnight. The brains were washed with PBS and cryoprotected in 30% sucrose at 4°C for two days before being immersed in optimal cutting temperature compound (Fisher, cat#23-730-571) and stored at −80°C. Brains were sectioned on a cryostat (Leica) and 30 μm floating sections were blocked and permeablized in 10% donkey serum with 0.2% Triton X-100 in PBS and then stained with primary antibodies against human nucleus protein (Chemicon, cat#MAB1281, dilution 1:500) and human GFAP (Sternberger, cat#SMI21, dilution 1:500) at 4°C overnight. Sections were washed three times with PBS and incubated for 2 h at room temperature with secondary antibodies followed by three additional PBS washes. The sections were then mounted on Superfrost Plus microscope slides (Fisher, cat#12-550-15) and covered with mounting medium (Fisher, cat#H1400NB) and glass coverslips.

For immunocytochemistry of cultured cells, we fixed and permeablized astrocytes with 4% PFA and 0.2% Triton-X100 in PBS. After blocking in 10% donkey serum, we stained astrocytes with primary antibody against NFκB p65 (1:200; Cell Signaling Technology, Cat#8242) and fluorescent secondary antibodies (Invitrogen). After three washes in PBS, we stained the cells with DAPI (Thermo Scientific, Cat#62248) and imaged them using an Evos FL Auto 2 inverted fluorescence microscope (Invitrogen) with 10x and 20x lenses. We used Photoshop CS5 and FIJI to process images.

### Statistical analysis and reproducibility

The numbers of patients, animals, and replicates are described in figures and figure legends. Experiments shown in the figures were repeated independently for the times listed below with similar results: Fig. 1a, 60 times. 1b, 10 times. 1c, twice. 2b-e, 12 times. 3b, 6 times with mouse samples and 3 times with human samples. Supplementary Fig. 10b, 7 times with mouse samples and 4 times with human samples. 25a, twice with mouse samples and 3 times with human samples. RNA-seq data were analyzed as described in the RNA-seq section above. For all non-RNA-seq data and RNA-seq data comparisons between species, analyses were conducted using RStudio (Version 1.3.1093) and Prism 8 software (Graphpad). Normality of data was tested by the Shapiro-Wilke test. For data with a normal distribution, Welch’s two-sided t test was used for two-group comparisons and a one-way ANOVA was used for multi-group comparisons. For data that deviate from the normal distribution, the Mann-Whitney test was used. Data from technical replicates from the same patient or the same litter of mice were averaged and used as a single biological replicate in statistical analyses. An estimate of the variation in each group is indicated by the standard error of the mean (SEM) or standard deviation (SD). *p < 0.05, **p < 0.01, ***p < 0.001.

### Reporting summary

Further information on research design is available in the Nature Research Reporting Summary linked to this article.

## Supplementary information

Supplementary Information

Description of Additional Supplementary Files

Supplementary Data 1

Supplementary Data 2

Supplementary Data 3

Supplementary Data 4

Supplementary Data 5

Supplementary Data 6

Supplementary Data 7

Supplementary Data 8

Supplementary Data 9

Supplementary Data 10

Supplementary Data 11

Supplementary Data 12

Supplementary Data 13

Reporting Summary

## Data Availability

All data supporting the findings of this study are provided within the paper and its supplementary information. A source data file is provided with this paper. We deposited all RNA-seq data to the Gene Expression Omnibus repository under accession number GSE147870. All additional information will be made available upon reasonable request to the authors. Source data are provided with this paper.

## Source data

Source data

## Code availability

All codes used in this study have been previously published^85,86^ and are available (STAR, HTseq, DESeq2, Limma, EdgeR). No custum code was generated in this study.

## References

[1] Sasaguri H (2017). APP mouse models for Alzheimer’s disease preclinical studies. EMBO J..

[2] Bezard E, Yue Z, Kirik D, Spillantini MG (2013). Animal models of Parkinson’s disease: Limits and relevance to neuroprotection studies. Mov. Disord..

[3] Masliah E (2000). Dopaminergic loss and inclusion body formation in alpha-synuclein mice: implications for neurodegenerative disorders. Science.

[4] Arnold ES (2013). ALS-linked TDP-43 mutations produce aberrant RNA splicing and adult-onset motor neuron disease without aggregation or loss of nuclear TDP-43. Proc. Natl Acad. Sci. U. S. A..

[5] Manwani B (2011). Functional recovery in aging mice after experimental stroke. Brain. Behav. Immun..

[6] Hay M, Thomas DW, Craighead JL, Economides C, Rosenthal J (2014). Clinical development success rates for investigational drugs. Nat. Biotechnol..

[7] Pfrieger FW, Barres BA (1997). Synaptic efficacy enhanced by glial cells in vitro. Science.

[8] Ullian EM, Sapperstein SK, Christopherson KS, Barres BA (2001). Control of synapse number by Glia. Sci. (80-).

[9] Blanco-Suarez E, Liu T-F, Kopelevich A, Allen NJ (2018). Astrocyte-secreted chordin-like 1 drives synapse maturation and limits plasticity by increasing synaptic GluA2 AMPA receptors. Neuron.

[10] Ma Z, Stork T, Bergles DE, Freeman MR (2016). Neuromodulators signal through astrocytes to alter neural circuit activity and behaviour. Nature.

[11] Huang YH, Sinha SR, Tanaka K, Rothstein JD, Bergles DE (2004). Astrocyte glutamate transporters regulate metabotropic glutamate receptor-mediated excitation of hippocampal interneurons. J. Neurosci..

[12] Parpura V (1994). Glutamate-mediated astrocyte–neuron signalling. Nature.

[13] Pascual O (2005). Astrocytic purinergic signaling coordinates synaptic networks. Science.

[14] Nedergaard M (1994). Direct signaling from astrocytes to neurons in cultures of mammalian brain cells. Science.

[15] Kelley KW (2018). Kir4.1-dependent astrocyte-fast motor neuron interactions are required for peak strength. Neuron.

[16] Chung W-S (2013). Astrocytes mediate synapse elimination through MEGF10 and MERTK pathways. Nature.

[17] Stogsdill JA (2017). Astrocytic neuroligins control astrocyte morphogenesis and synaptogenesis. Nature.

[18] Anderson MA (2016). Astrocyte scar formation aids central nervous system axon regeneration. Nature.

[19] Yu X (2018). Reducing astrocyte calcium signaling in vivo alters striatal microcircuits and causes repetitive behavior. Neuron.

[20] Molofsky AV (2014). Astrocyte-encoded positional cues maintain sensorimotor circuit integrity. Nature.

[21] Eroglu Ç (2009). Gabapentin receptor α2δ-1 is a neuronal thrombospondin receptor responsible for excitatory CNS synaptogenesis. Cell.

[22] Allen NJ (2012). Astrocyte glypicans 4 and 6 promote formation of excitatory synapses via GluA1 AMPA receptors. Nature.

[23] Farhy-Tselnicker I (2017). Astrocyte-secreted glypican 4 regulates release of neuronal pentraxin 1 from axons to induce functional synapse formation. Neuron.

[24] Oberheim NA (2009). Uniquely hominid features of adult human astrocytes. J. Neurosci..

[25] Oberheim NA, Wang X, Goldman S, Nedergaard M (2006). Astrocytic complexity distinguishes the human brain. Trends Neurosci..

[26] Zhang Y (2016). Purification and characterization of progenitor and mature human astrocytes reveals transcriptional and functional differences with mouse. Neuron.

[27] Zamanian JL (2012). Genomic analysis of reactive astrogliosis. J. Neurosci..

[28] Zhang Y (2014). An RNA-sequencing transcriptome and splicing database of glia, neurons, and vascular cells of the cerebral cortex. J. Neurosci..

[29] Hodge RD (2019). Conserved cell types with divergent features in human versus mouse cortex. Nature.

[30] Han X (2013). Forebrain engraftment by human glial progenitor cells enhances synaptic plasticity and learning in adult mice. Cell Stem Cell.

[31] Windrem MS (2014). A competitive advantage by neonatally engrafted human glial progenitors yields mice whose brains are chimeric for human glia. J. Neurosci..

[32] Windrem MS (2008). Neonatal chimerization with human glial progenitor cells can both remyelinate and rescue the otherwise lethally hypomyelinated shiverer mouse. Cell Stem Cell.

[33] Krencik R, Weick JP, Liu Y, Zhang Z-J, Zhang S-C (2011). Specification of transplantable astroglial subtypes from human pluripotent stem cells. Nat. Biotechnol..

[34] Tchieu J (2019). NFIA is a gliogenic switch enabling rapid derivation of functional human astrocytes from pluripotent stem cells. Nat. Biotechnol..

[35] Sloan SA (2017). Human astrocyte maturation captured in 3D cerebral cortical spheroids derived from pluripotent stem cells. Neuron.

[36] Chai H (2017). Neural circuit-specialized astrocytes: transcriptomic, proteomic, morphological, and functional evidence. Neuron.

[37] Crowley, L. C., Christensen, M. E. & Waterhouse, N. J. Measuring mitochondrial transmembrane potential by TMRE staining. *Cold Spring Harb. Protoc.*10.1101/pdb.prot087361 (2016). 10.1101/pdb.prot087361 27934682

[38] Dringen R, Pawlowski PG, Hirrlinger J (2005). Peroxide detoxification by brain cells. J. Neurosci. Res..

[39] Ma X (2011). Mitochondrial electron transport chain complex III is required for antimycin A to inhibit autophagy. Chem. Biol..

[40] Nordgren, M. & Fransen, M. Peroxisomal metabolism and oxidative stress. *Biochimie* **98**, 56–62 (2014). 10.1016/j.biochi.2013.07.026 23933092

[41] Foo LC (2011). Development of a method for the purification and culture of rodent astrocytes. Neuron.

[42] Petriv, O. I. & Rachubinski, R. A. Lack of peroxisomal catalase causes a progeric phenotype in caenorhabditis elegans. *J. Biol. Chem.* **279**, P19996–20001 (2004). 10.1074/jbc.M400207200 14996832

[43] Xu, Y. et al. Glucose-6-phosphate dehydrogenase-deficient mice have increased renal oxidative stress and increased albuminuria. *FASEB J.* **24**, 609–616 (2010). 10.1096/fj.09-135731 PMC2812032 19805580

[44] Ho, H. Y., Cheng, M. L. & Chiu, D. T. Y. Glucose-6-phosphate dehydrogenase - From oxidative stress to cellular functions and degenerative diseases. *Redox Report* **12**, 109–118 (2007). 10.1179/135100007X200209 17623517

[45] Bakken, T. E. et al. Single-cell RNA-seq uncovers shared and distinct axes of variation in dorsal LGN neurons in mice, non-human primates and humans. bioRxiv 2020.11.05.367482 (2020). 10.1101/2020.11.05.367482 10.7554/eLife.64875 PMC8412930 34473054

[46] Minnerup J, Sutherland BA, Buchan AM, Kleinschnitz C (2012). Neuroprotection for stroke: current status and future perspectives. Int. J. Mol. Sci..

[47] Michalicová, A., Bhide, K., Bhide, M. & Kováč, A. How viruses infiltrate the central nervous system *Acta Virol.* **61**, 393–400 (2017). 10.4149/av_2017_401 29186956

[48] Pellegrini, L. et al. SARS-CoV-2 infects the brain choroid plexus and disrupts the blood-CSF barrier in human brain organoids. *Cell Stem Cell*10.1016/j.stem.2020.10.001 (2020). 10.1016/j.stem.2020.10.001 PMC7553118 33113348

[49] Perriot S (2018). Human induced pluripotent stem cell-derived astrocytes are differentially activated by multiple sclerosis-associated cytokines. Stem Cell Reports.

[50] Sharma, D., Kim, M. S. & D’Mello, S. R. Transcriptome profiling of expression changes during neuronal death by RNA-Seq. *Exp. Biol. Med.* **240**, 242–251 (2015). 10.1177/1535370214551688 PMC4640449 25258427

[51] Mathys, H. et al. Single-cell transcriptomic analysis of Alzheimer’s disease. *Nature* **571**, 332–337 (2019). 10.1038/s41586-019-1195-2 PMC6865822 31042697

[52] Zhou, Y. et al. Human and mouse single-nucleus transcriptomics reveal TREM2-dependent and TREM2-independent cellular responses in Alzheimer’s disease. *Nat. Med.*10.1038/s41591-019-0695-9 (2020). 10.1038/s41591-019-0695-9 PMC6980793 31932797

[53] Schirmer, L. et al. Neuronal vulnerability and multilineage diversity in multiple sclerosis. *Nature* **573**, 75–82 (2019). 10.1038/s41586-019-1404-z PMC6731122 31316211

[54] Wheeler, M. A. et al. MAFG-driven astrocytes promote CNS inflammation. *Nature* **578**, 593–599 (2020). 10.1038/s41586-020-1999-0 PMC8049843 32051591

[55] Jäkel, S. et al. Altered human oligodendrocyte heterogeneity in multiple sclerosis. *Nature* **566**, 543–547 (2019). 10.1038/s41586-019-0903-2 PMC6544546 30747918

[56] Henrik Heiland, D. et al. Tumor-associated reactive astrocytes aid the evolution of immunosuppressive environment in glioblastoma. *Nat. Commun.* **10**, 2541 (2019). 10.1038/s41467-019-10493-6 PMC6559986 31186414

[57] Liddelow SA (2017). Neurotoxic reactive astrocytes are induced by activated microglia. Nature.

[58] Guttenplan, K. A. et al. Neurotoxic reactive astrocytes drive neuronal death after retinal injury. *Cell Rep.* **31**, 107776 (2020). 10.1016/j.celrep.2020.107776 PMC8091906 32579912

[59] Chang, C. C., Zhang, J., Lombardi, L., Neri, A. & Dalla-Favera, R. Mechanism of expression and role in transcriptional control of the proto-oncogene NFKB-2/LYT-10. *Oncogene* **9**, 923–933 (1994). 8108136

[60] Habib, N. et al. Massively parallel single-nucleus RNA-seq with DroNc-seq. *Nat. Methods* **14**, 955–958 (2017). 10.1038/nmeth.4407 PMC5623139 28846088

[61] Habib, N. et al. Disease-associated astrocytes in Alzheimer’s disease and aging. *Nat. Neurosci.* **23**, 701–706 (2020). 10.1038/s41593-020-0624-8 PMC9262034 32341542

[62] Ito M (2018). RNA-sequencing analysis revealed a distinct motor cortex transcriptome in spontaneously recovered mice after stroke. Stroke.

[63] Mekel-Bobrov N (2005). Ongoing adaptive evolution of ASPM, a brain size determinant in Homo sapiens. Science.

[64] Florio M (2015). Human-specific gene ARHGAP11B promotes basal progenitor amplification and neocortex expansion. Science.

[65] Long KR (2018). Extracellular matrix components HAPLN1, lumican, and collagen I cause hyaluronic acid-dependent folding of the developing human neocortex. Neuron.

[66] Kalebic N (2019). Neocortical expansion due to increased proliferation of basal progenitors is linked to changes in their morphology. Cell Stem Cell.

[67] Wang X, Tsai J-W, LaMonica B, Kriegstein AR (2011). A new subtype of progenitor cell in the mouse embryonic neocortex. Nat. Neurosci..

[68] Hansen DV, Lui JH, Parker PRL, Kriegstein AR (2010). Neurogenic radial glia in the outer subventricular zone of human neocortex. Nature.

[69] Namba T (2020). Human-specific ARHGAP11B acts in mitochondria to expand neocortical progenitors by glutaminolysis. Neuron.

[70] Johnson MB (2009). Functional and evolutionary insights into human brain development through global transcriptome analysis. Neuron.

[71] Kang HJ (2011). Spatio-temporal transcriptome of the human brain. Nature.

[72] Miller JA (2014). Transcriptional landscape of the prenatal human brain. Nature.

[73] Jacobs RA, Díaz V, Meinild A, Gassmann M, Lundby C (2013). The C57Bl/6 mouse serves as a suitable model of human skeletal muscle mitochondrial function. Exp. Physiol..

[74] Perlman RL (2016). Mouse models of human disease: an evolutionary perspective. Evol. Med. public Heal..

[75] Billingsley KJ (2019). Mitochondria function associated genes contribute to Parkinson’s Disease risk and later age at onset. npj Park. Dis.

[76] Arneson D (2018). Single cell molecular alterations reveal target cells and pathways of concussive brain injury. Nat. Commun..

[77] Le Douce J (2020). Impairment of glycolysis-derived l-serine production in astrocytes contributes to cognitive deficits in Alzheimer’s disease. Cell Metab..

[78] Choi BH, Lapham LW (1978). Radial glia in the human fetal cerebrum: A combined golgi, immunofluorescent and electron microscopic study. Brain Res.

[79] Roessmann U, Gambetti P (1986). Astrocytes in the developing human brain. Acta Neuropathol..

[80] Elder GA, Major EO (1988). Early appearance of type II astrocytes in developing human fetal brain. Dev. Brain Res..

[81] Molofsky AV, Deneen B (2015). Astrocyte development: a guide for the perplexed. Glia.

[82] Bushong EA, Martone ME, Ellisman MH (2004). Maturation of astrocyte morphology and the establishment of astrocyte domains during postnatal hippocampal development. Int. J. Dev. Neurosci..

[83] Zhong S (2020). Decoding the development of the human hippocampus. Nature.

[84] Neuner, S. M., Heuer, S. E., Huentelman, M. J., O’Connell, K. M. S. & Kaczorowski, C. C. Harnessing genetic complexity to enhance translatability of Alzheimer’s disease mouse models: a path toward precision medicine. *Neuron* **101**, 399–411 (2019). 10.1016/j.neuron.2018.11.040 PMC6886697 30595332

[85] Liao Y, Smyth GK, Shi W (2019). The R package Rsubread is easier, faster, cheaper and better for alignment and quantification of RNA sequencing reads. Nucleic Acids Res.

[86] Love, M. I., Huber, W. & Anders, S. Moderated estimation of fold change and dispersion for RNA-seq data with DESeq2. *Genome Biol.* **15**, 550 (2014). 10.1186/s13059-014-0550-8 PMC4302049 25516281

