# Supplementary material for: Conservation and divergence of vulnerability and responses to stressors between human and mouse astrocytes
Source: Nat Commun. 2021 Jun 25;12:3958. doi: 10.1038/s41467-021-24232-3 (PMC8233314; doi:10.1038/s41467-021-24232-3)
Supplement: Supplementary file 1 — Supplementary Information [file 41467_2021_24232_MOESM1_ESM.pdf]

Supplementary Information

Conservation and divergence of vulnerability and responses to stressors between human and mouse astrocytes

Supplementary Figures

Supplementary Figure 1

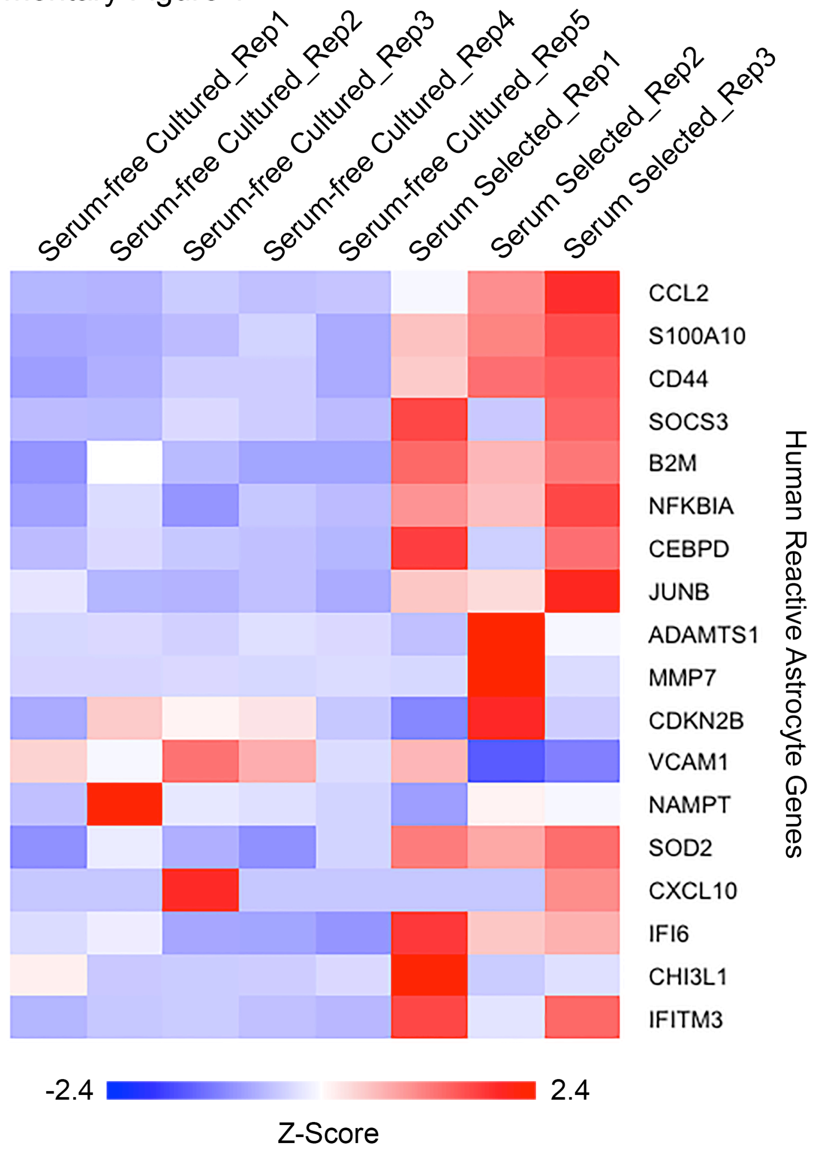

Supplementary Figure 1. Expression of reactive astrocyte genes by serum-free and serum-selected cultures of human astrocytes

Eighteen protein-coding genes induced by both poly I:C and TNFα (FDR < 0.05; Maximum RPKM > 5; Fold change > 4) are shown.

Supplementary Figure 2

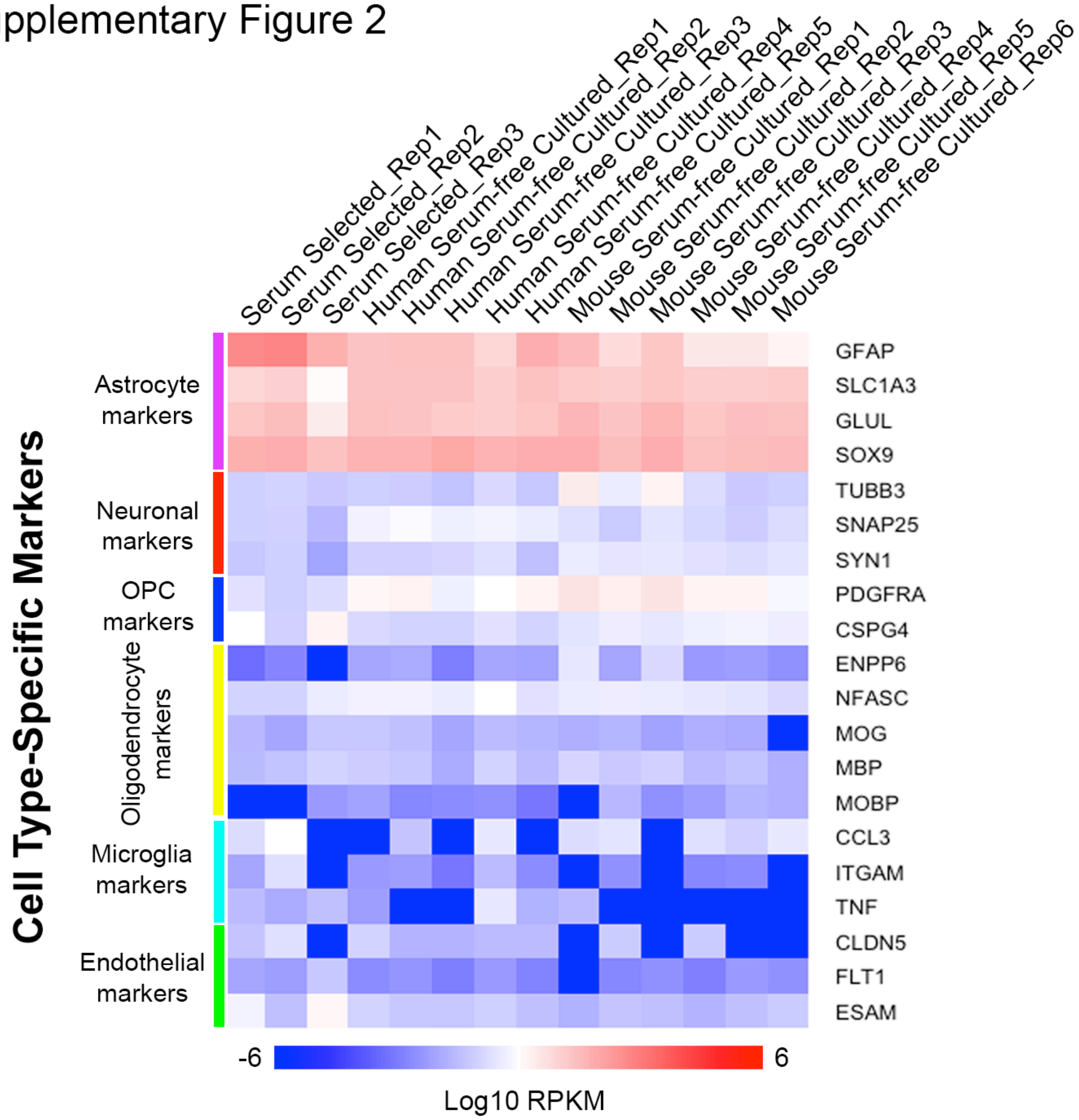

Supplementary Figure 2. Purity of serum-selected and serum-free cultures of human and mouse astrocytes

The expression of cell-type-specific markers (logRPKM) is shown for serum-selected human astrocytes and serum-free cultures of human and mouse astrocytes.

Supplementary Figure 3

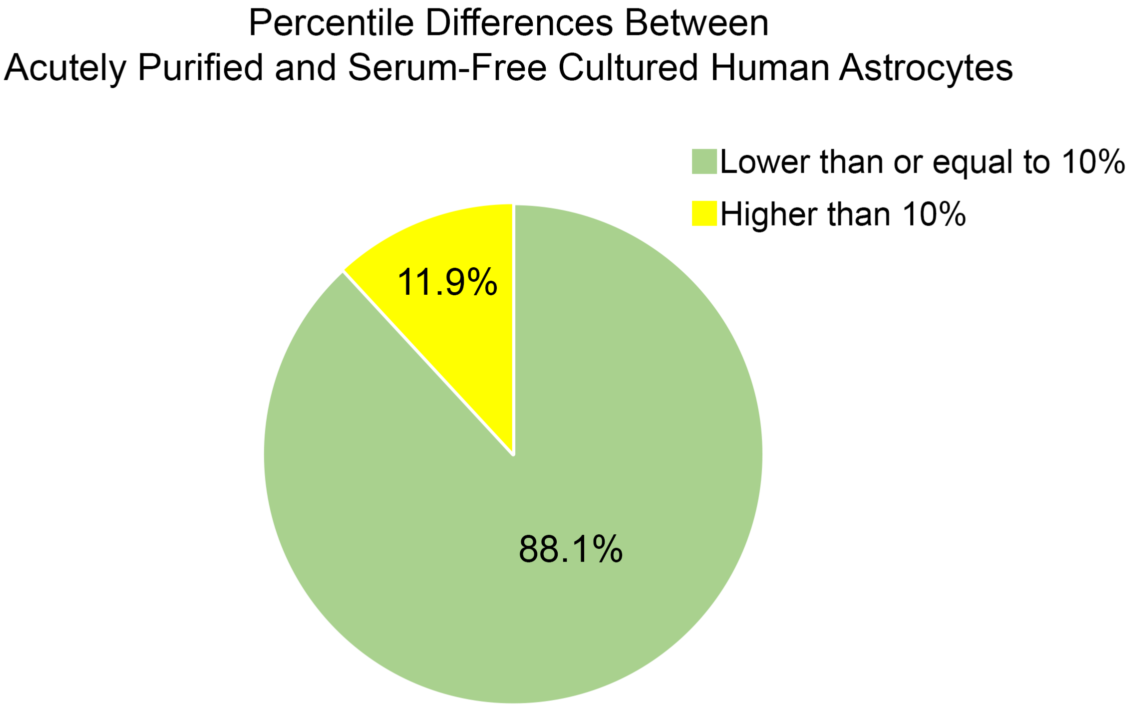

Supplementary Figure 3. Differences in percentile ranking of gene expression between serum-free cultured and acutely purified human astrocytes
The numbers of genes that changed decile (> 10% difference in percentile ranking) or stayed in the same decile (< 10% difference) between the two conditions are shown. The majority (88.1%) of the genes are in the same decile in cultured and acutely purified astrocytes.

Supplementary Figure 4

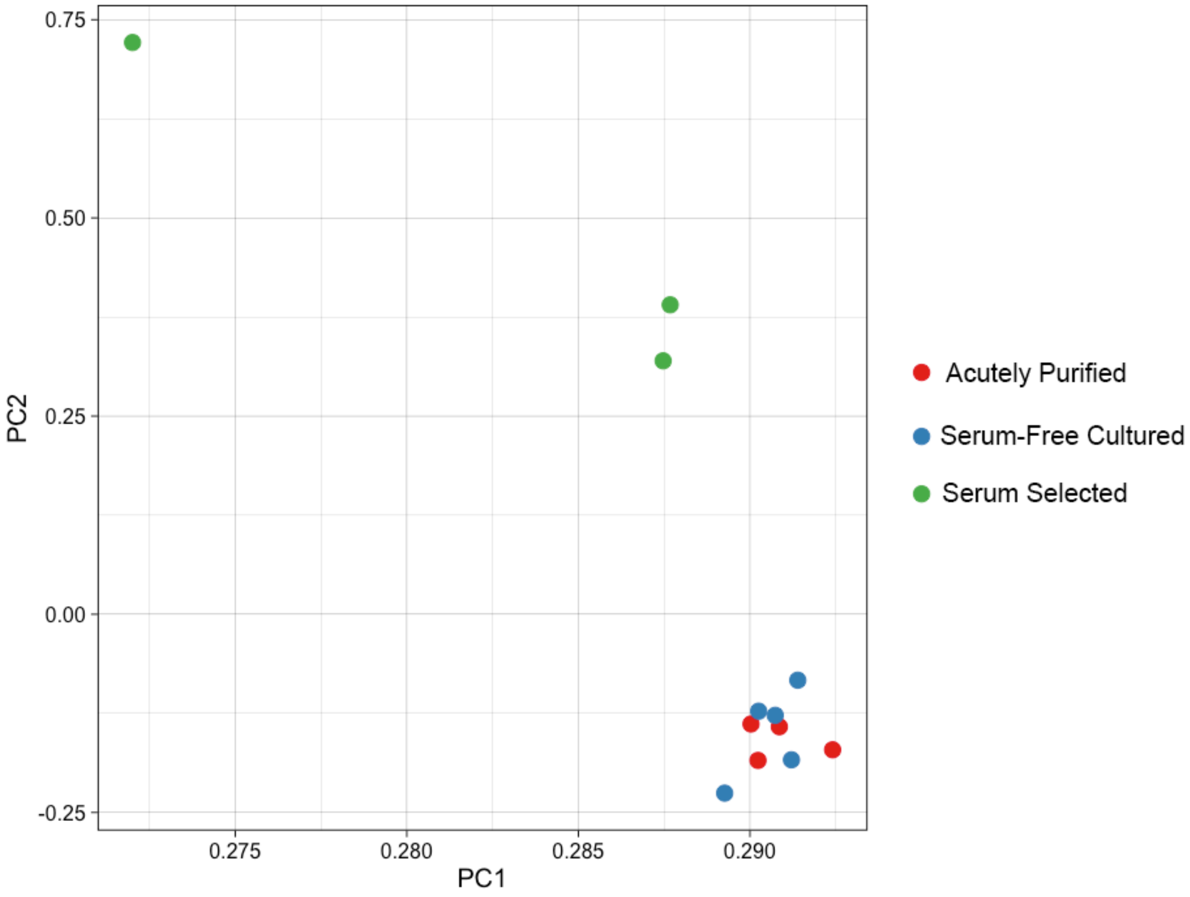

Supplementary Figure 4. Principal component analysis of acutely purified, serum-free culture, and serum-selected culture of human astrocytes
LogRPKM values of all protein-coding genes are shown. Principal component (PC)1 and PC2 explained 89.0 and 2.5% of the variance, respectively.

Supplementary Figure 5

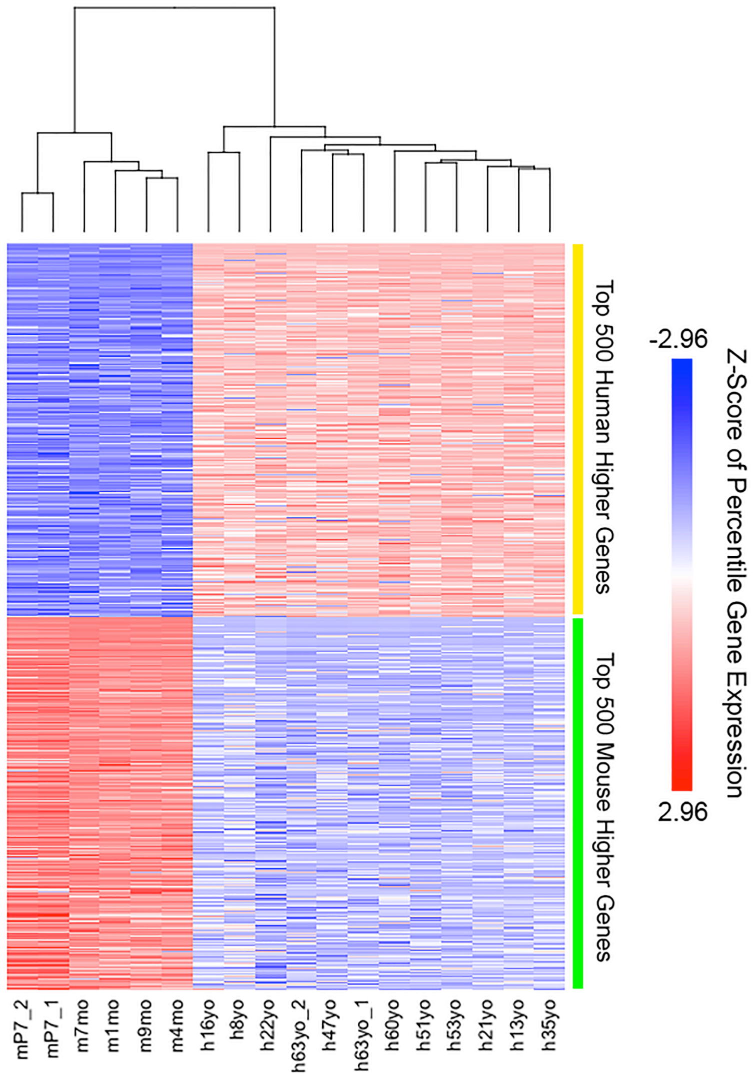

Supplementary Figure 5. Heatmap of the 1,000 genes with the largest expression differences between acutely purified human and mouse astrocytes
m and h indicate the species of the samples. P7, postnatal day 7. Mo, months old. Yo, years old.

Supplementary Figure 6

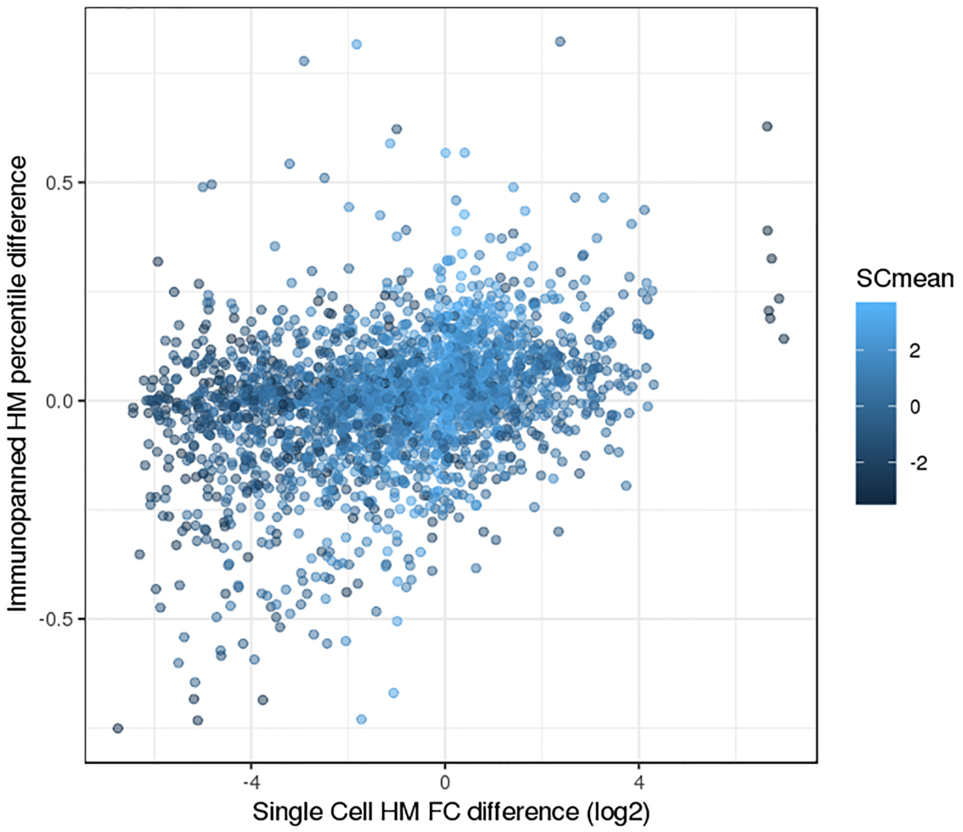

Supplementary Figure 6. Human-mouse differences in astrocytic gene expression determined by bulk RNA-seq of immunopanned astrocytes and single-cell RNA-seq

For each gene, the expression difference between human and mouse determined by bulk RNA-seq of immunopanned astrocytes is plotted on the Y-axis and that determined by single-cell RNA-seq (astrocyte cluster only) is plotted on the X-axis. We detected a significant correlation between the two methods ($r^2 = 0.35$).

Supplementary Figure 7

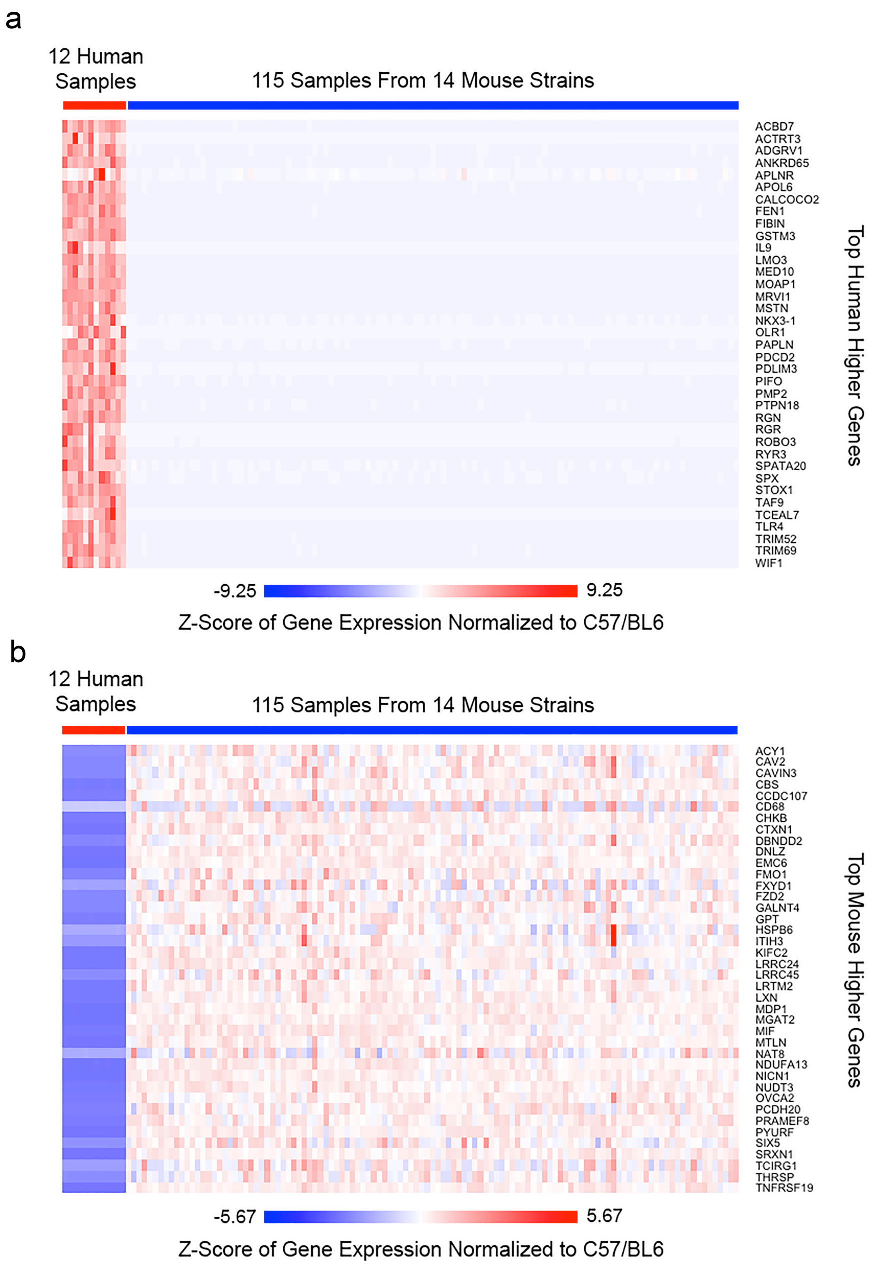

Supplementary Figure 7. Gene expression across multiple mouse strains
We first compared astrocytic gene expression from humans and C57/BL6 mice and identified the top 50 genes with expression higher in humans than in mice and those with expression higher in mice than in humans. To assess the expression of these genes across multiple mouse strains, we normalized the expression of these genes in humans to their levels in C57/BL6 mice determined by our study and similarly normalized the expression of these genes in 14 different mouse strains to their levels in C57/BL6 mice from a previous report[86]. The Z-scores of normalized expression of these genes are shown.

Supplementary Figure 8

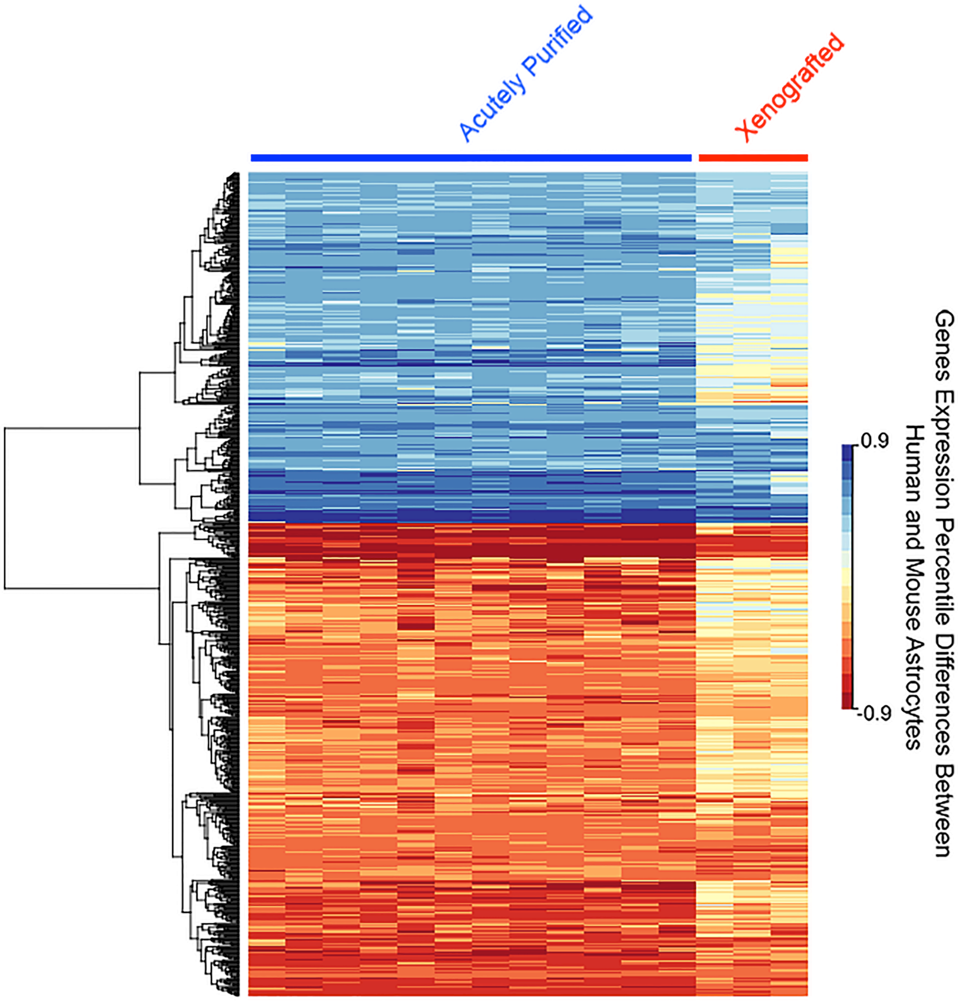

Supplementary Figure 8. Human vs. mouse gene expression differences in acutely purified and xenografted astrocytes.

Species differences in gene expression (shown as percentile ranking in human minus percentile ranking in mouse) in xenografted and acutely purified astrocytes highly correlate. Genes with percentile rankings > 0.33, species-difference FDR < 0.05, and species differences in percentile rankings > 0.4 are shown.

Supplementary Figure 9

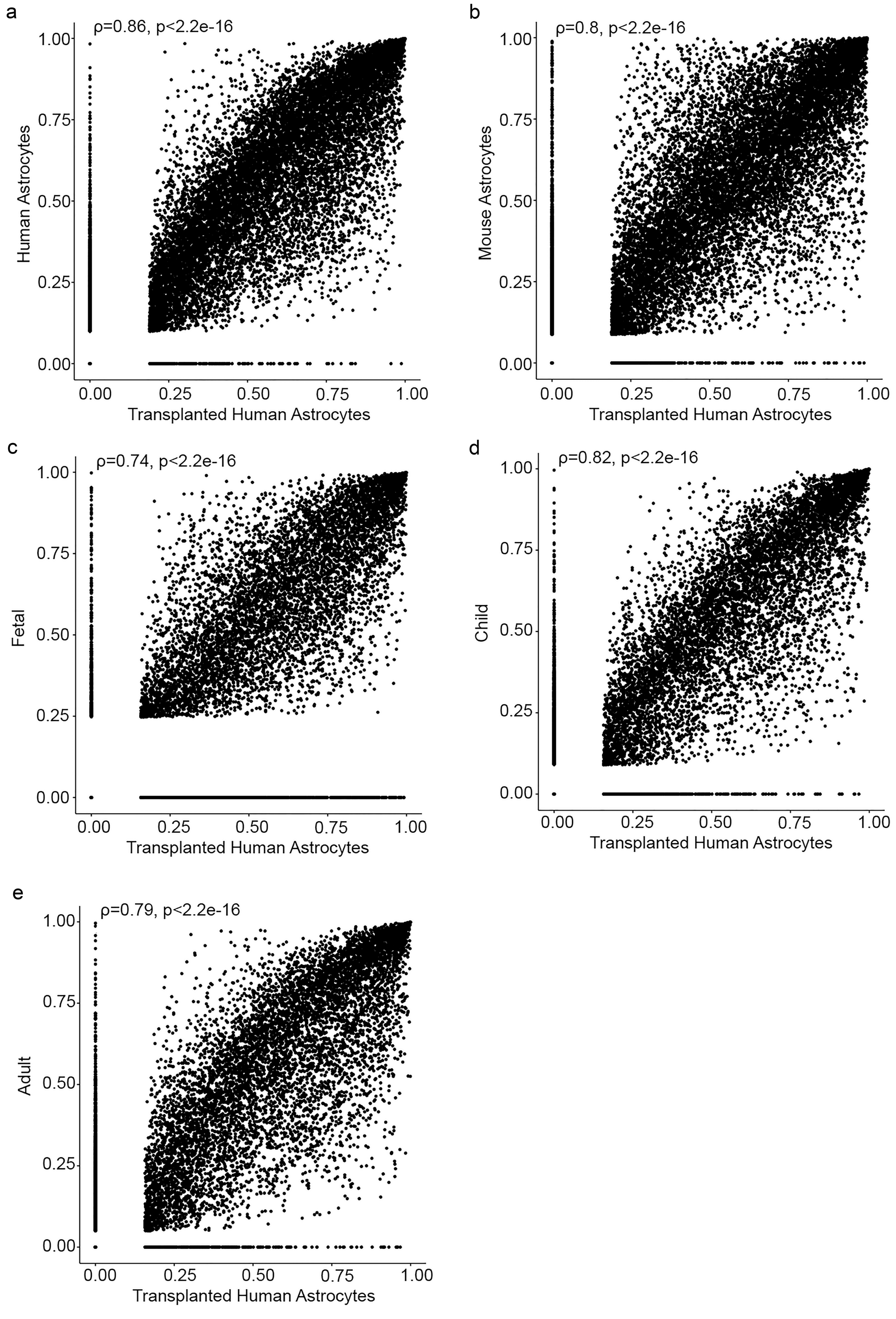

Supplementary Figure 9. Correlation between gene expression of xenografted and acutely purified astrocytes.

Spearman's correlation coefficient ρ and p-values are shown in all panels. Two-tailed t-test.

(a) Acutely purified human astrocytes (all developing and adult ages) vs. transplanted human astrocytes. Only protein-coding genes were included.

(b) Acutely purified mouse astrocytes (all developing and adult ages) vs. transplanted human astrocytes. Only protein-coding genes were included.

(c) Acutely purified human fetal astrocytes vs. transplanted human astrocytes. Protein-coding genes with average RPKM > 0 were included.

(d) Acutely purified human children's astrocytes vs. transplanted human astrocytes. Protein-coding genes with average RPKM > 0 were included.

(e) Acutely purified human adult astrocytes vs. transplanted human astrocytes. Protein-coding genes with average RPKM > 0 were included.

Supplementary Figure 10

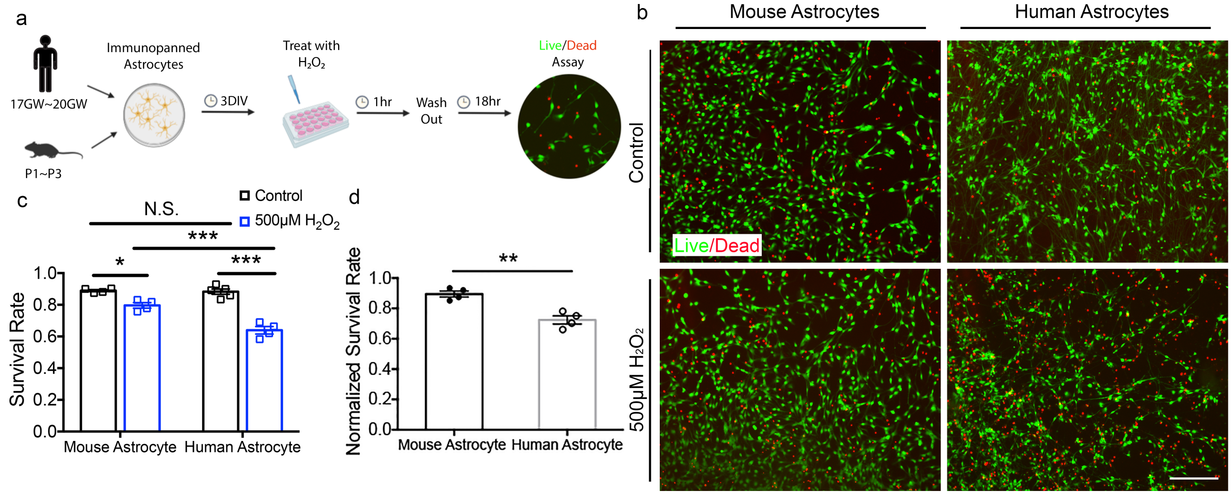

Supplementary Figure 10. Survival of human and mouse astrocytes under oxidative stress.

(a) Experimental design.

(b) Human and mouse astrocytes treated with H_2O_2 or medium control stained with the live cell dye calcein-AM (green) and the dead cell dye ethidium homodimer (red). Scale bar: 200 μm. This experiment was performed once as shown here and repeated independently six times in mouse astrocytes and three times in human astrocytes as shown in Figure 3B.

(c) Survival rate. Mouse: N = 4 images from one culture per group (H_2O_2 treated group, control group) generated from one litter of mice. Human: N = 5 images from one control culture and 4 images from one H_2O_2-treated culture generated from one patient. Data are presented as mean ± SEM. Mouse astrocyte: control *vs.* H_2O_2, $p = 0.012$. Mouse astrocytes-H_2O_2-treated *vs.* human astrocyte-H_2O_2-treated, $p = 0.0002$. Human astrocyte: control *vs.* H_2O_2, $p < 0.0001$. *, $p < 0.05$. **, $p < 0.01$. ***, $p < 0.001$, N.S., not significant. Two-way analysis of variance (ANOVA) with Tukey's test for multiple comparisons. The p-values were calculated using each image as an independent observation.

(d) Survival rate of astrocytes treated with H_2O_2 normalized to the survival rate of medium control-treated cells. Mouse astrocytes: N = 4 images from one culture per group generated from one litter of mice. Human astrocytes: N = 5 images from one control culture and 4 images from one H_2O_2-treated culture generated from one patient. Data are presented as mean ± SEM. $p=0.0027$. *, $p < 0.05$. **, $p < 0.01$. ***, $p < 0.001$. Two-tailed unpaired Welch's t-test.

Supplementary Figure 11

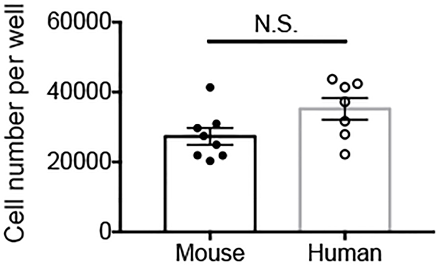

Supplementary Figure 11. Densities of human and mouse astrocytes in Seahorse respirometry experiments did not differ

We plated human and mouse astrocytes at a series of initial densities and only used wells with similar final densities. The numbers of cells per well on the day of the Seahorse experiments are shown. Mouse: N = 8 cultures generated from 4 litters of mice. Human: N = 7 cultures generated from 3 patients. Data are presented as mean ± SEM. $p = 0.0677$. N.S., not significant. Two-tailed unpaired Welch's t-test.

Supplementary Figure 12

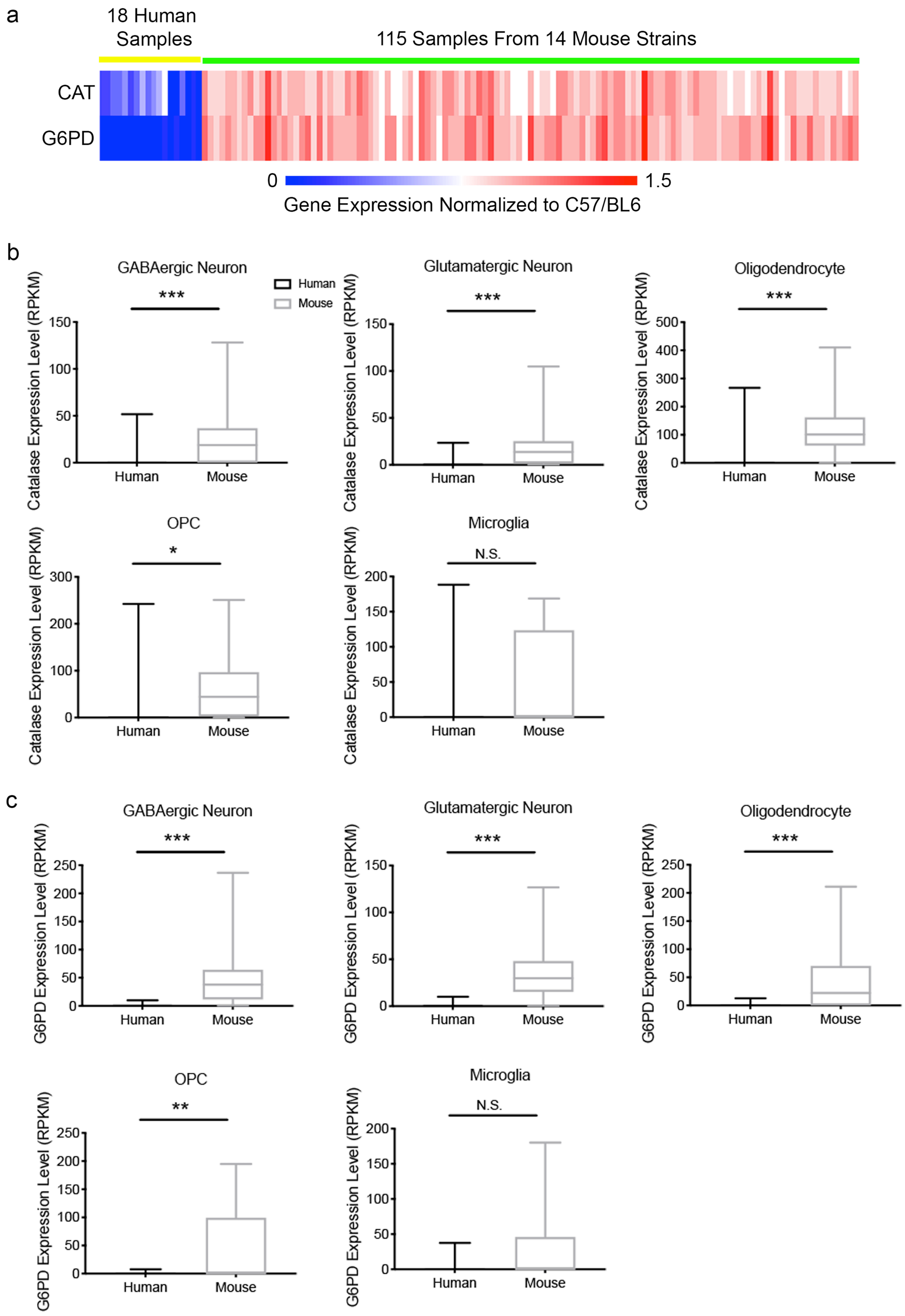

Supplementary Figure 12. The expression of Cat and G6pd genes in different mouse strains and in different cell types

(a) As in Supplementary Figure 7, the expression of Cat and G6pd (homolog G6pdx in mouse) in human samples and mouse samples from 14 different strains were normalized to their expression in C57/BL6 mice. The Z-scores of normalized gene expression are shown.

(b-c) The expression of Cat and G6pd by major brain cell types in humans and mice is shown based on single-cell RNA-seq[39,100]. N = 231 human GABAergic neurons, 688 mouse GABAergic neurons (Catalase expression, $p < 0.0001$; G6PD expression, $p < 0.0001$). N = 1027 human glutamatergic neurons, 1090 mouse glutamatergic neurons (Catalase expression, $p < 0.0001$; G6PD expression, $p < 0.0001$). N = 188 human oligodendrocytes, 59 mouse oligodendrocytes (Catalase expression, $p < 0.0001$; G6PD expression, $p < 0.0001$). N = 26 human OPCs, 27 mouse OPCs (Catalase expression, $p = 0.0166$; G6PD expression, $p = 0.0062$). N = 26 human microglia, 6 mouse microglia. Data are presented as boxes-and-whiskers with the wishers span from minima to maxima of each dataset, the boxes extend from the 25th to 75th percentiles and the centre line indicates the median. *, $p < 0.05$. **, $p < 0.01$. ***, $p < 0.001$. Two-tailed Welch's unpaired t-test with Bonferroni correction for multiple comparisons. N.S., not significant.

Supplementary Figure 13

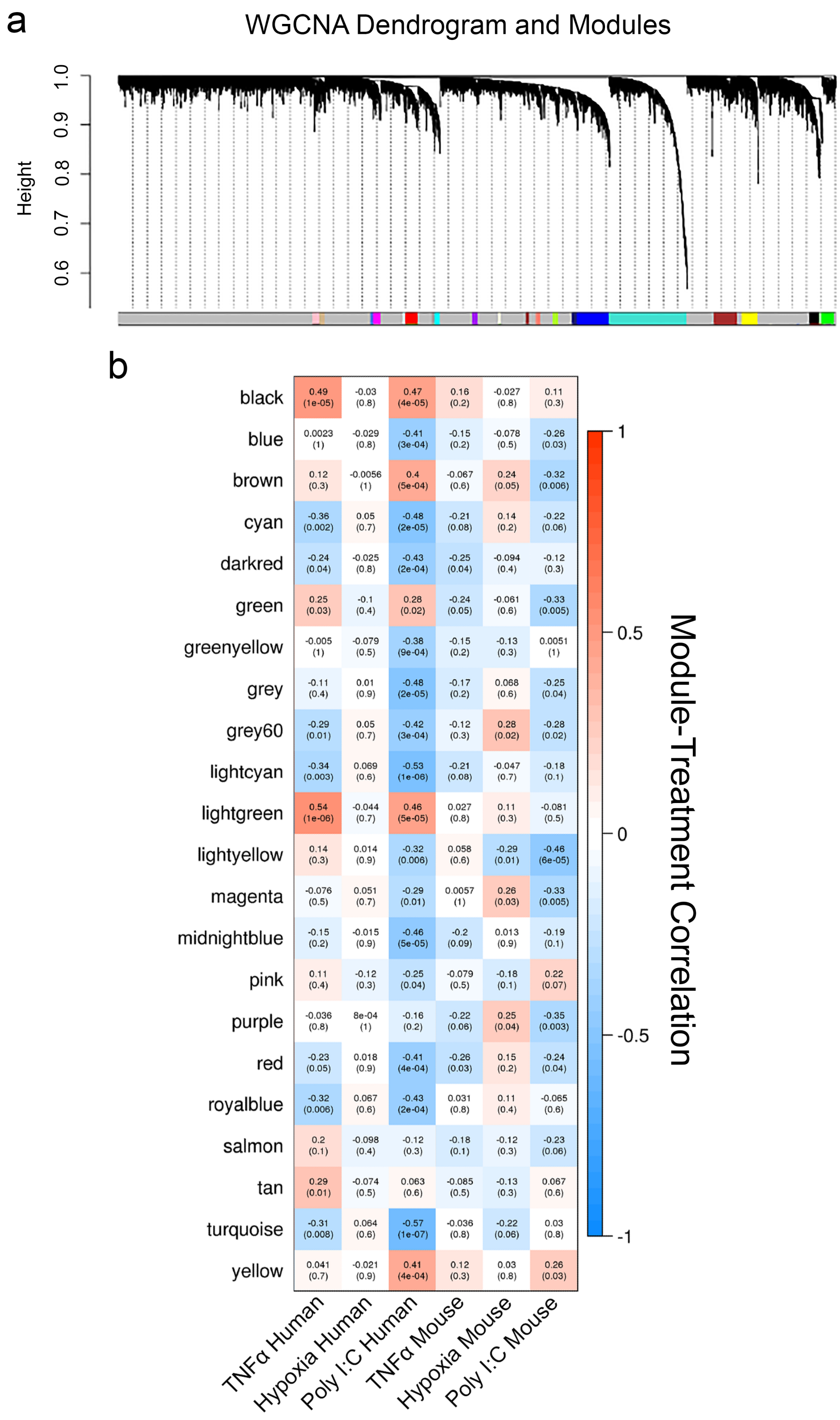

Supplementary Figure 13. WGCNA identified gene coexpression modules

(a) WGCNA dendrogram. Data for all conditions were combined for the analyses.

(b) Modules and their association with treatment conditions. The numbers on top in each rectangle represent correlation and the numbers on the bottom in parentheses are p-values. The p-values are the Student's asymptotic p-values for correlation from the WGCNA package. Two-tailed Student's t-test.

Supplementary Figure 14

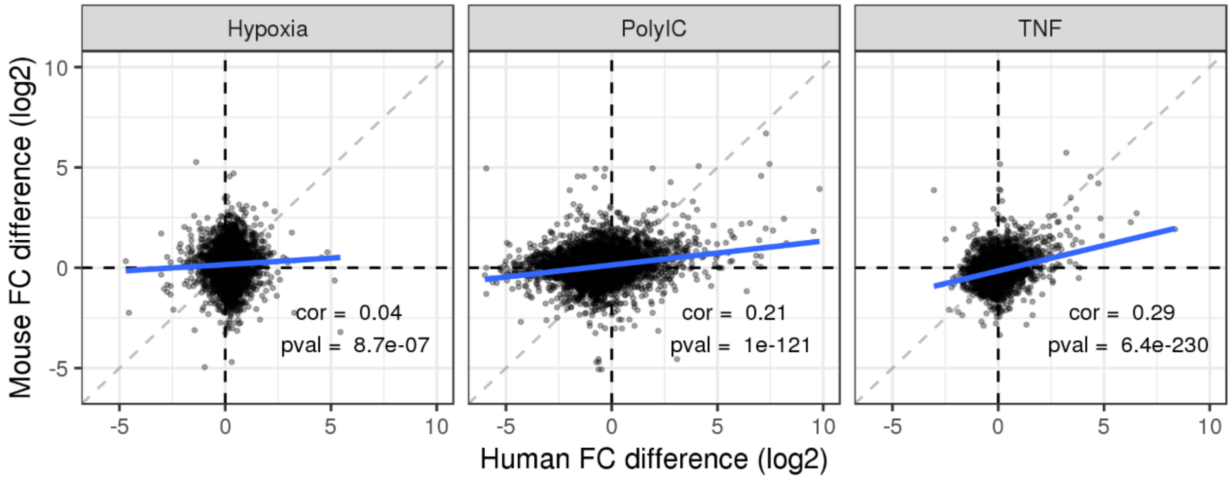

Supplementary Figure 14. Scatter plots and correlations of hypoxia, poly I:C, and TNFα treatment-induced gene expression changes in human and mouse astrocytes

Genes with average count < 1 were filtered out. Two-tailed t-test.

Supplementary Figure 15

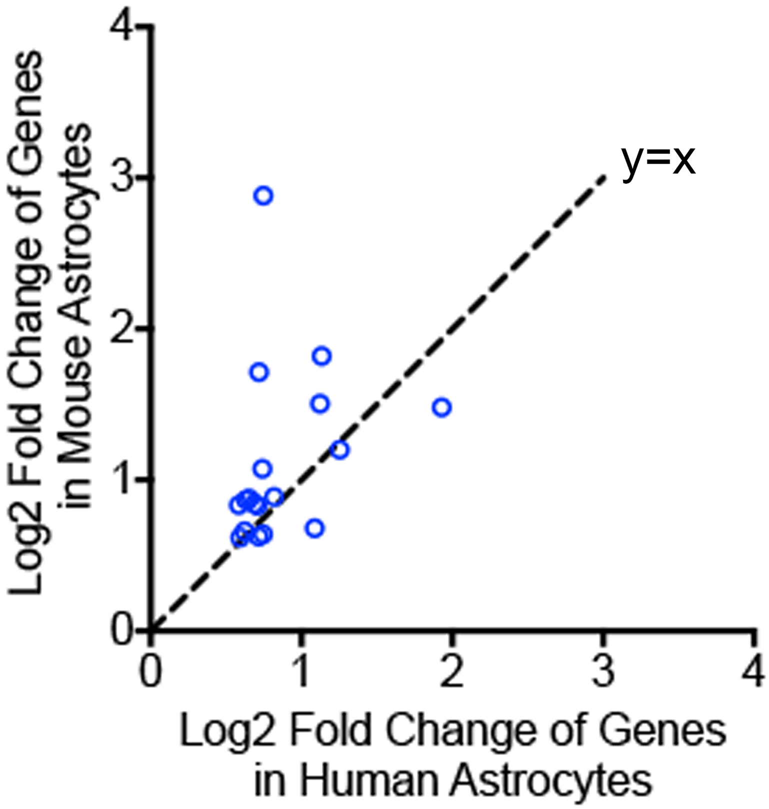

Supplementary Figure 15. Comparison of fold change of hypoxia-induced genes in human and mouse astrocytes.
Genes with FDR < 0.05 in both species, FC > 1.5, and average RPKM values of control/treated > 1 are included. Only genes that showed significant change in both species are included. Black line: predicted trendline of y = x if there were no species differences.

Supplementary Figure 16

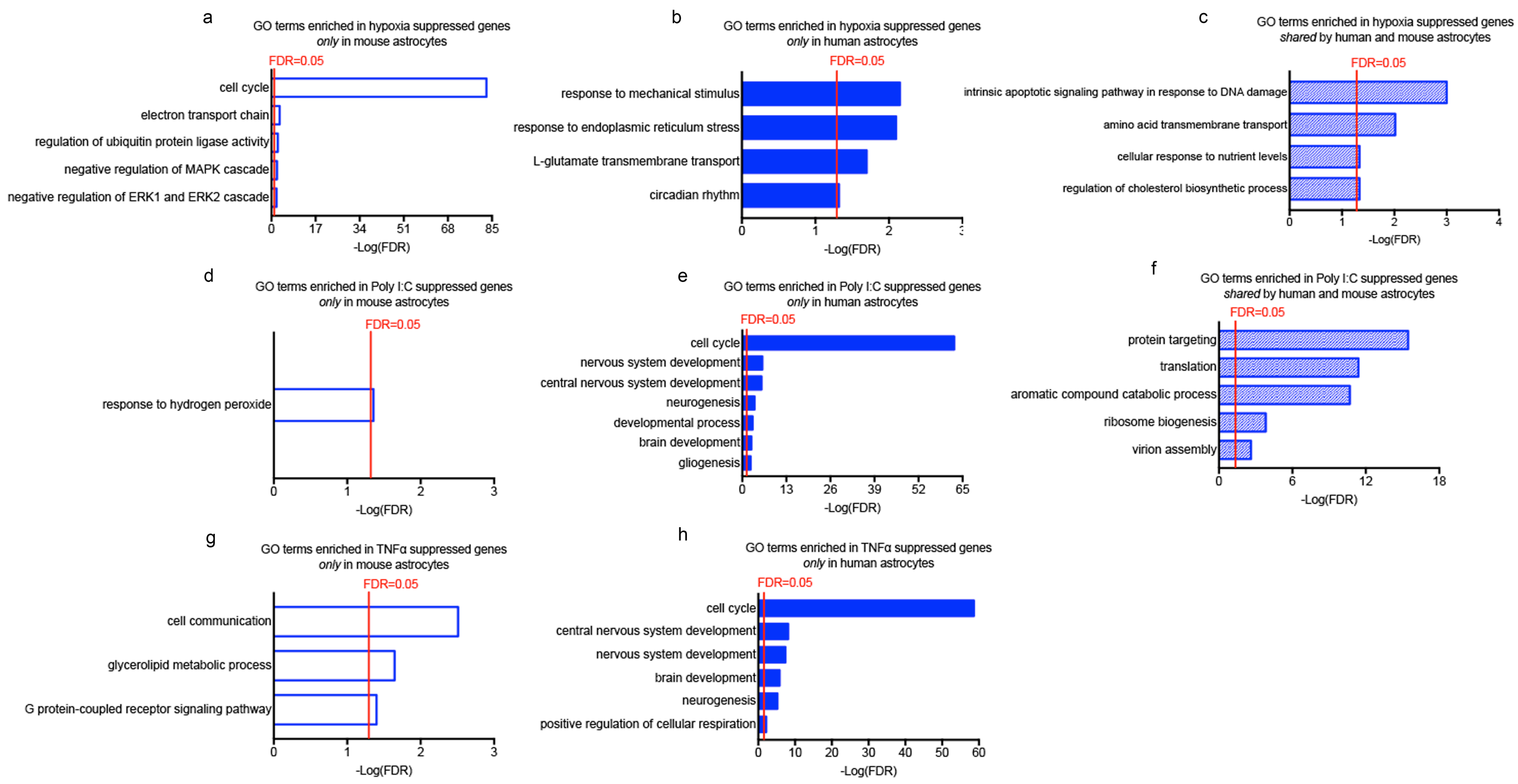

Supplementary Figure 16. GO terms associated with downregulated genes in hypoxia, poly I:C, and TNFα treatments

The red lines indicate FDR = 0.05.

Supplementary Figure 17

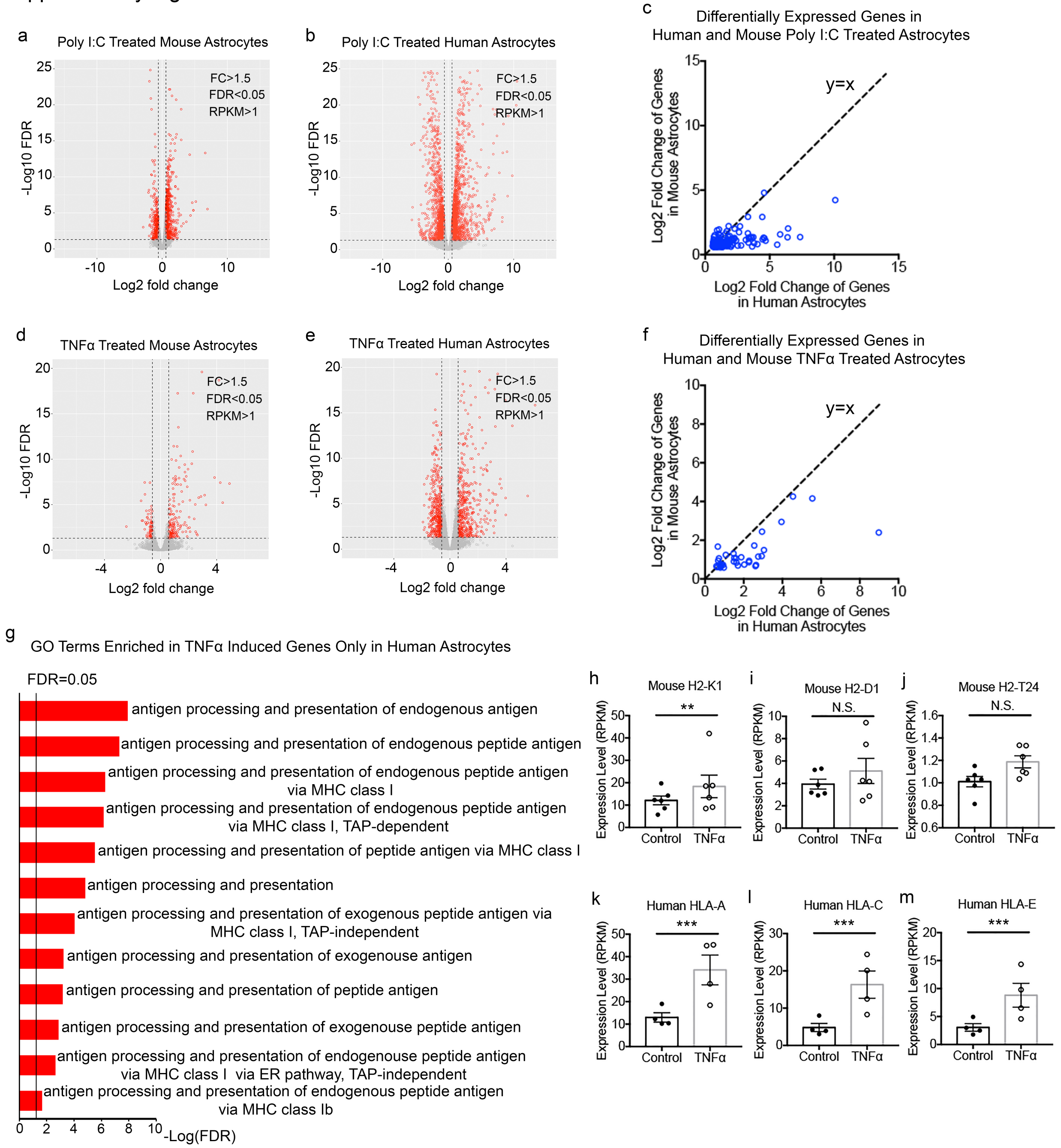

Supplementary Figure 17. Molecular responses of human and mouse astrocytes to poly I:C or TNFα

(a,b,d,e) Volcano plots of genes significantly different between poly I:C- or TNFα-treated and control conditions. Each red dot represents a significantly different gene.

(c, f) Comparison of fold change of poly I:C- or TNFα-induced genes in human and mouse astrocytes. Genes with FDR < 0.05 in both species, FC > 1.5, and average RPKM values of control/treated >1 are included. Black line: predicted trendline of y = x if there were no species differences.

(g) Antigen presentation-related GO terms enriched in TNFα-induced genes *only* in human astrocytes.
(h-m) Expression of top 3 highest expressing MHC Class I antigen presentation genes in TNFα-treated and control human and mouse astrocytes. N = 6 litters of mice and 4 human patients. Data are presented as mean ± SEM. Mouse H2-K1, $p = 0.0089$. Human HLA-A, $p < 0.0001$; human HLA-C, $p < 0.0001$; human HLA-E, $p < 0.0001$. *, $p < 0.05$. **, $p < 0.01$. ***, $p < 0.001$ by the DEseq2 package[25]. N.S., not significant.

Supplementary Figure 18

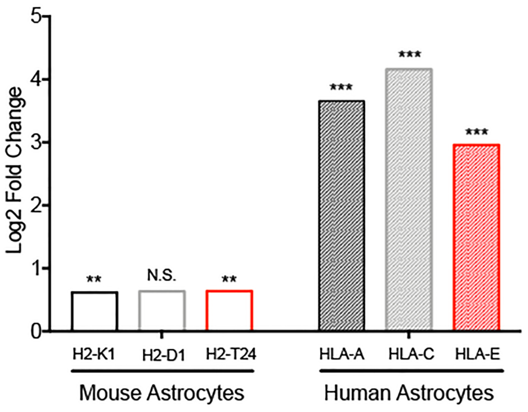

Supplementary Figure 18. Fold change of top 3 highest-expressing MHC Class I antigen presentation genes in poly I:C-treated and control human and mouse astrocytes. Mouse H2-K1, $p = 0.0051$; mouse H2-T24, $p = 0.0019$. Human HLA-A, $p < 0.0001$; Human HLA-C, $p < 0.0001$; Human HLA-E, $p < 0.0001$. *, $p < 0.05$. **, $p < 0.01$. ***, $p < 0.001$ by the DESeq2 package[25]. N.S., not significant.

Supplementary Figure 19

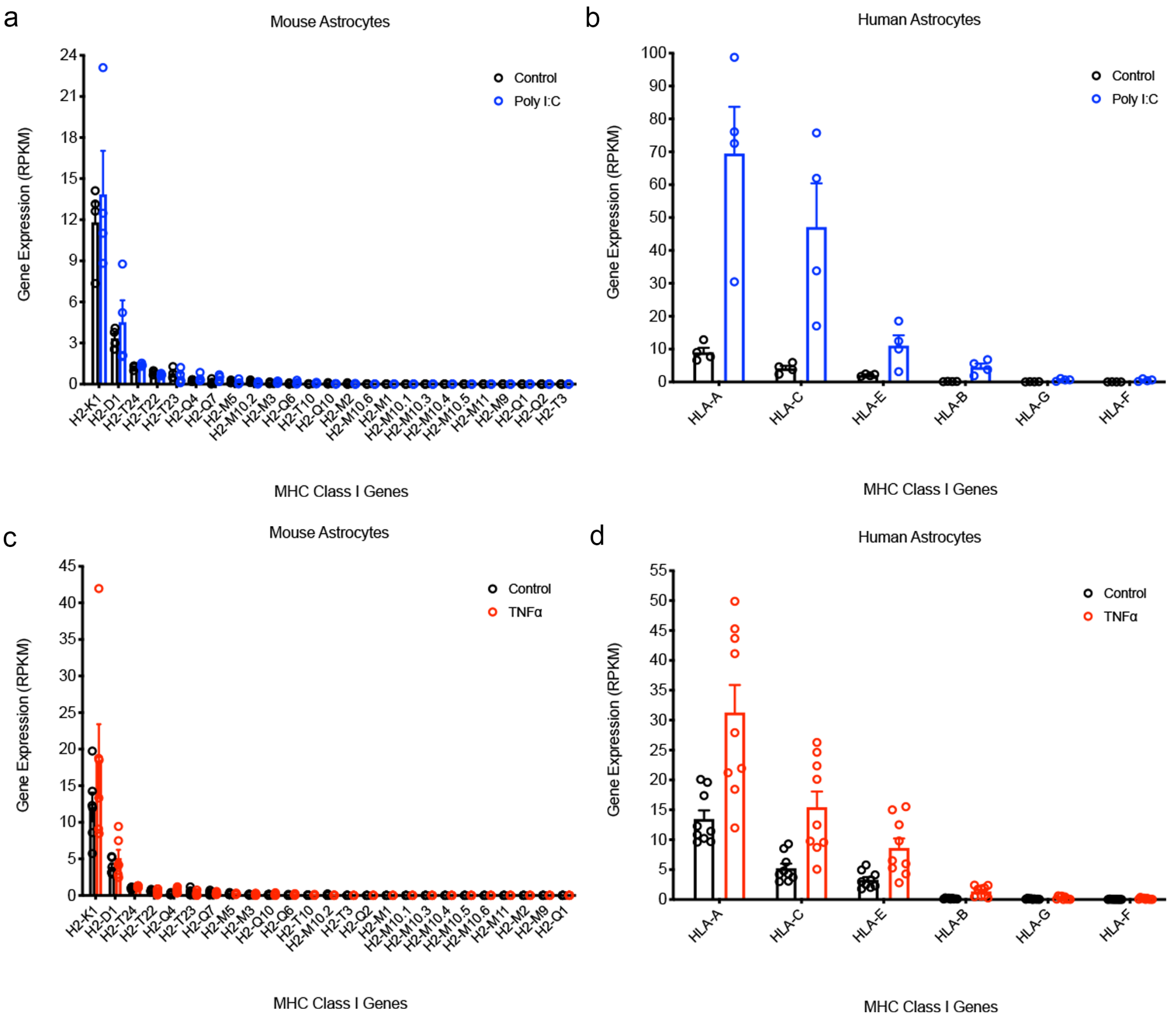

Supplementary Figure 19. Expression of MHC Class I genes in poly I:C and TNFα-treated human and mouse astrocytes

RPKM values of all MHC Class I genes are shown in the bar charts. Poly I:C treatment: N = 4 cultures generated from 4 litter of mice or 4 patients. TNFα treatment: N = 6 cultures generated from 6 litter of mice, 9 cultures generated from 4 patients. Data are presented as mean ± SEM.

Supplementary Figure 20

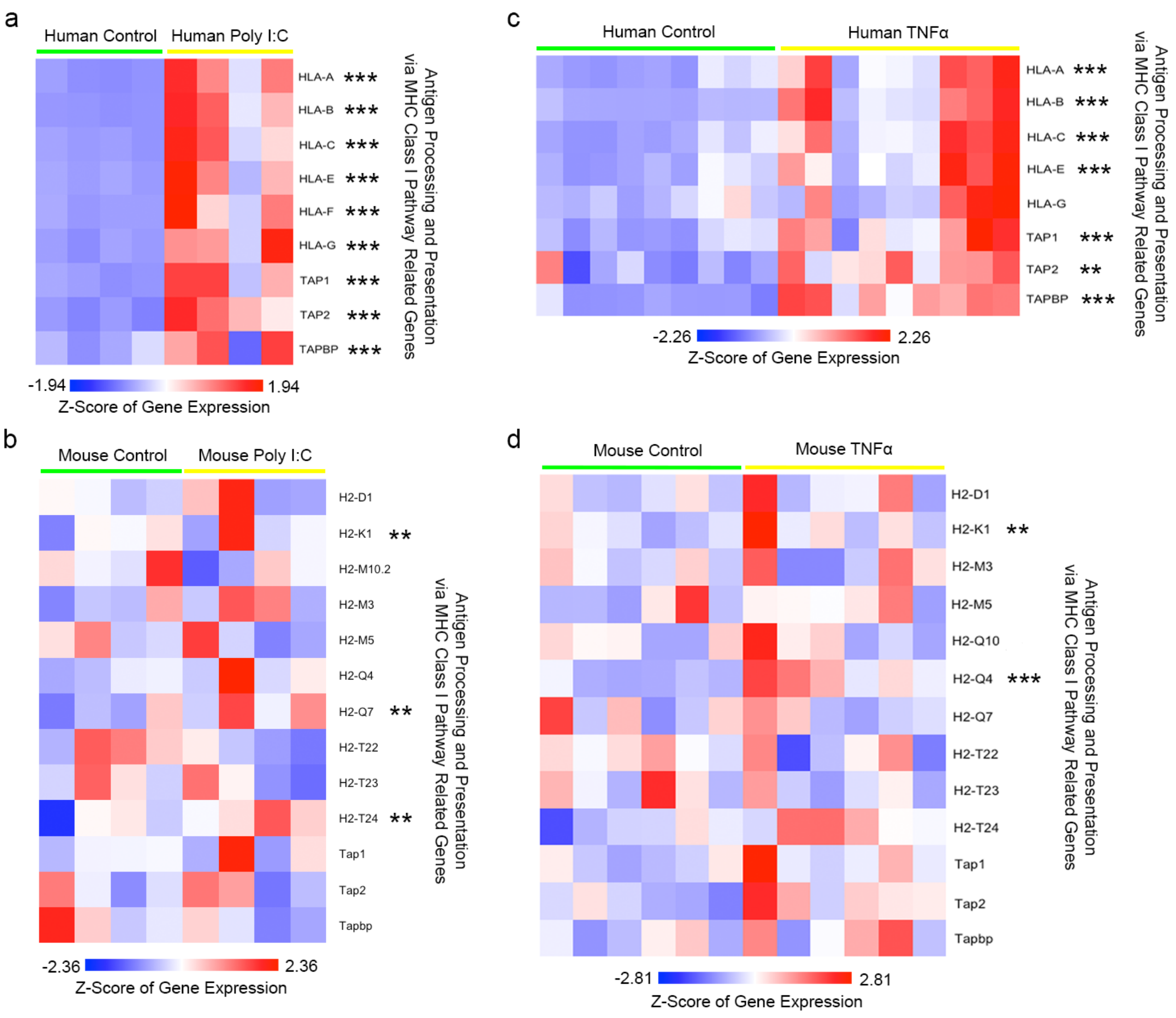

Supplementary Figure 20. Heatmap of the expression of antigen processing and presentation via MHC Class I pathway-associated genes

All genes with average RPKM > 0.1 across all samples are shown. Asterisks on the right of the heatmaps indicate significant differences between poly I:C and control or TNFα and control determined using DESeq2[25]. (a) Differences of gene expression between poly I:C-treated and control human astrocytes: HLA-A, $p < 0.0001$; HLA-B, $p < 0.0001$; HLA-C, $p < 0.0001$; HLA-E, $p < 0.0001$; HLA-F, $p < 0.0001$; HLA-G, $p < 0.0001$; TAP1, $p < 0.0001$; TAP2, $p < 0.0001$; TAPBP, $p < 0.0001$. (b) Differences of gene expression between poly I:C-treated and control mouse astrocytes: H2-K1, $p = 0.0051$; H2-Q7, $p = 0.006$; H2-T24, $p = 0.0019$. (c) Differences of gene expression between TNFα-treated and control human astrocytes: HLA-A, $p < 0.0001$; HLA-B, $p < 0.0001$; HLA-C, $p < 0.0001$; HLA-E, $p < 0.0001$; TAP1, $p < 0.0001$; TAP2, $p = 0.0048$; TAPBP, $p < 0.0001$. (d) Differences of gene expression between TNFα-treated and control mouse astrocytes: H2-K1, $p = 0.0089$; H2-Q4, $p = 0.0002$. *, $p < 0.05$. **, $p < 0.01$. ***, $p < 0.001$.

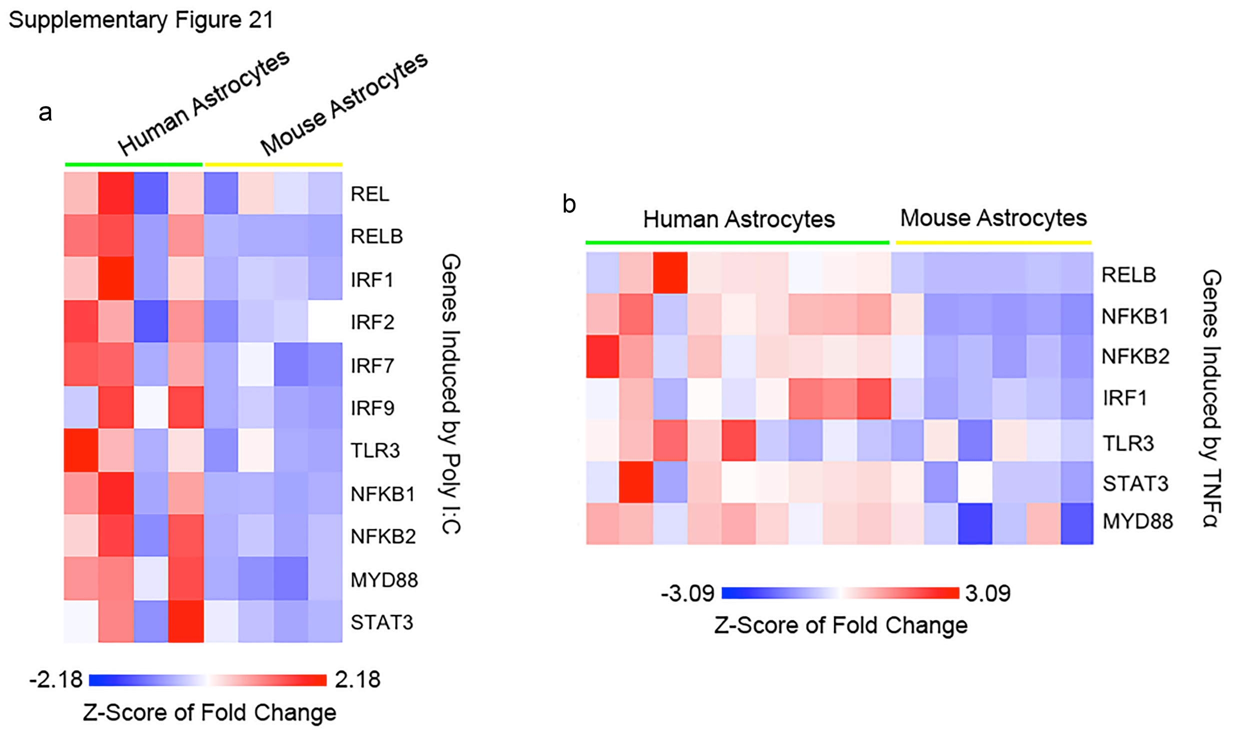

Supplementary Figure 21. Heatmap of examples of poly I:C- and TNFα-induced gene expression changes in human and mouse astrocytes

Gene expression fold change between treatment and control is shown for each individual sample.

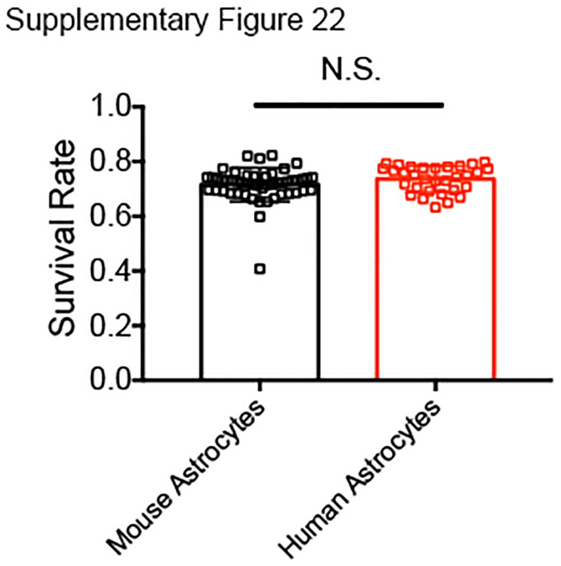

Supplementary Figure 22. Survival of human and mouse astrocytes in culture
The survival rates of cultured human and mouse astrocytes were determined by staining with live cell dye (Calcein-AM) and dead cell dye (ethidium homo dimer). There was no significant difference between the survival of cultured human and mouse astrocytes. N = 50 images from 12 cultures generated from 6 litters of mice and 31 images from 7 cultures generated from 3 human patients. Data are presented as mean ± SD. Two-tailed Mann-Whitney test. N.S., not significant.

Supplementary Figure 23

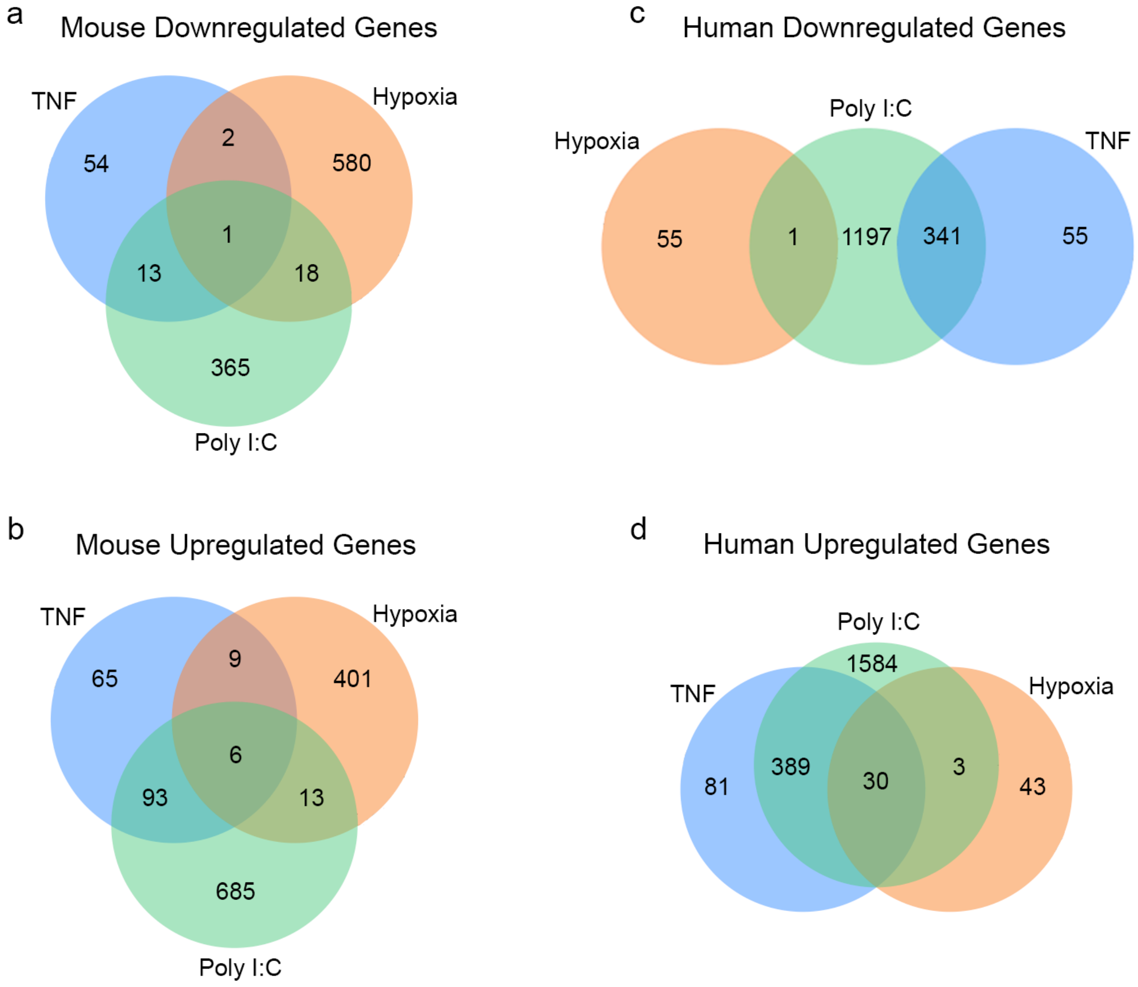

Supplementary Figure 23. Comparison of genes induced by hypoxia, poly I:C, and TNFα treatment

Protein-coding genes with FDR < 0.05 and fold change > 1.5 are included.

Supplementary Figure 24

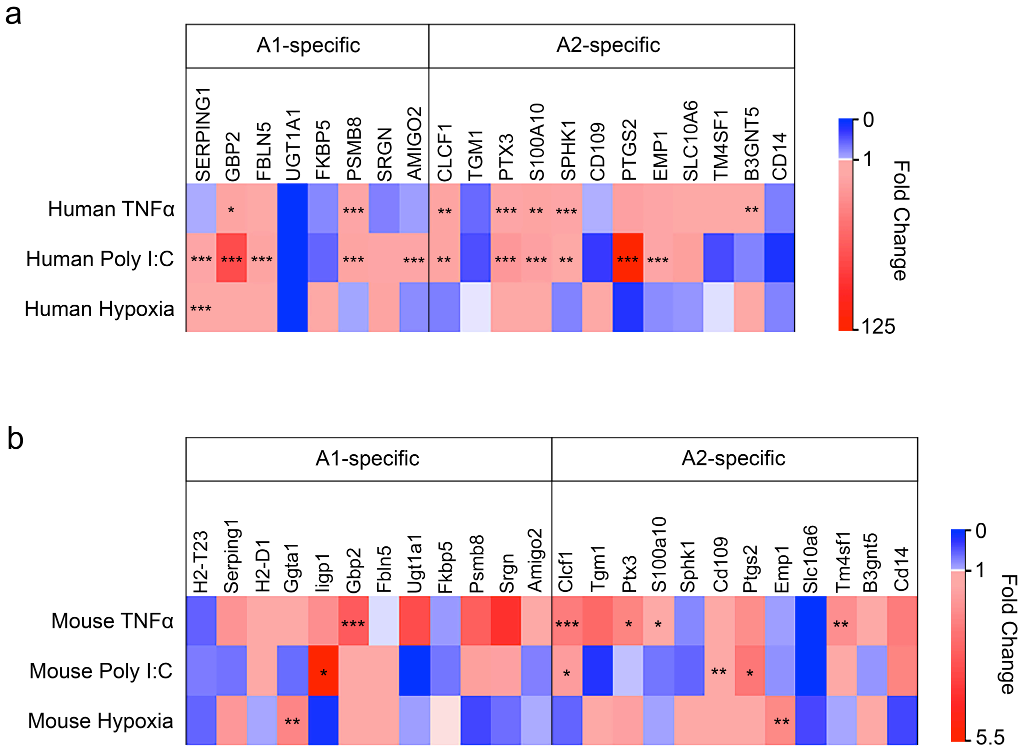

Supplementary Figure 24. Comparison of hypoxia, poly I:C, and TNFα treatment-induced gene expression changes with A1/A2-astrocyte marker genes

The list of A1-specific and A2-specific genes is based on previous publications[94,97,106,107]. Note that some of these genes are not present in the human genome. Treatment-induced fold change in gene expression is shown. * indicates significant changes based on DESeq2 analysis[25]. *, $p < 0.05$. **, $p < 0.01$. ***, $p < 0.001$.

Supplementary Figure 25

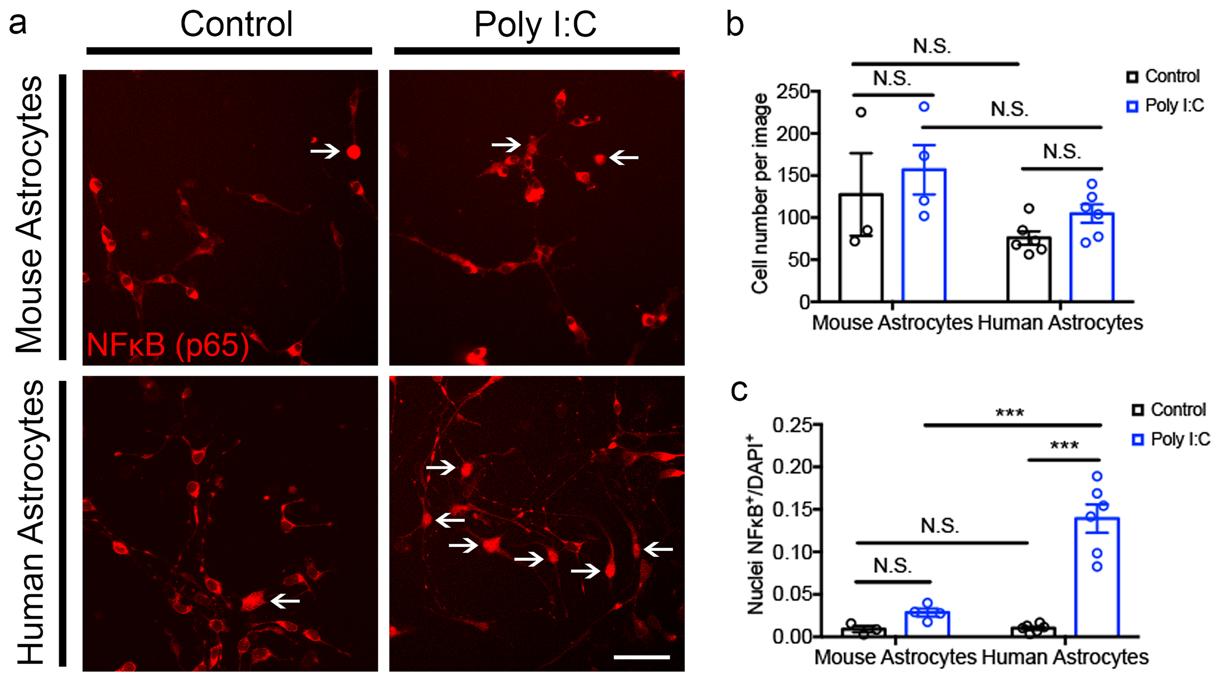

Supplementary Figure 25. Activation of NFκB in a subpopulation of poly I:C-treated human and mouse astrocytes

(a) Immunofluorescence of NFκB (p65). Nuclear localization of p65 indicates activation of NFκB signaling. Arrows point to p65 localized in the nuclei. Scale bar: 50 μm. The experiment was repeated independently three times with human samples and twice with mouse samples.

(b) Cell numbers per image. No difference between any conditions. Data are presented as mean ± SEM. Two-way ANOVA with Tukey's multiple comparison test. N.S., not significant. Human: N = 6 cultures per condition from 3 patients. Mouse: N = 3 control cultures and 4 poly I:C-treated cultures from 2 litters of mice.

(c) Quantification of the percentage of cells with nuclear-localized p65. Data are presented as mean ± SEM. Two-way ANOVA with Tukey's multiple comparison test. Human: N = 6 cultures per condition from 3 patients. Mouse: N = 3 control cultures and 4 poly I:C-treated cultures from 2 litters of mice. Human astrocytes, control *vs.* poly I:C, $p < 0.0001$. Mouse astrocytes poly I:C *vs.* human astrocytes poly I:C, $p < 0.0001$. *, $p < 0.05$. **, $p < 0.01$. ***, $p < 0.001$. N.S., not significant.

Supplementary Figure 26

a

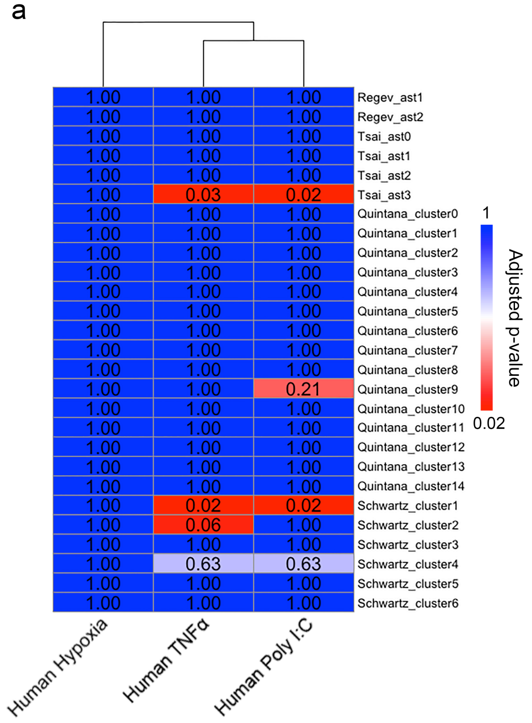

b

		Tsai_ast3	Schwartz_cluster1
Human TNFα	Upregulated	SOD2, CHI3L1, IFITM3, B2M, CD44, OSMR, GFAP, ELL2, IGFBP7, ITGB4, MGST1, NRCAM, BCL6, MFAP3L, GLIS3, PLSCR1, ARHGEF3, ZFP36, SLC39A14, TNFRSF1A, ETV6, STAT3, ITGA6, NAV2, CAST, CPNE8, FGF2, RTN4, NRP1, TPST1, SMAD1, F3, TMBIM1, SAMD4A, LIMS1, MFHAS1, RGL1, ST8SIA1, CNN3, DAP3, DST, FLOT1, SORBS1, CLIP1	IRAK2, NHSL1
Human TNFα	Downregulated	TUBB2B, REV3L, MAPRE2, PLCE1, INPP5F, TMTC2, LRIG1, PSD3, RALGAPA2, NAV1, C3orf70, DNER, MAGI1, TRIM9	AUTS2, LSAMP, GRIA2, CTNND2, RORA, ADGRB3, NCAM2, PCDH7, FRY, MERTK, KCND2, NRXN1, CDH10, ST6GALNAC5, FGFR2, NNAT, MAP2K6, GRM3, MDGA2, GRID2, CSGALNACT1, CADM2, MGAT4C, KIRREL3, LUZP2
Human Poly I:C	Upregulated	CHI3L1, SOD2, IFITM3, B2M, IGFBP7, PLSCR1, ITGB4, OSMR, CD44, GLIS3, ELL2, FGF2, CPNE8, MFAP3L, MGST1, SLC39A14, BCL6, GFAP, ZFYVE28, NRCAM, ZFP36, ETV6, ITGAV, ARHGEF3, SMAD1, F3, PRRX1, WWC1, CAST, GALNT18, RTN4, SLC44A3, LIMS1, NAV2, STAT3, KCND3, SLC20A2, DTNA, PPFIA1, CLIP1, CNN3, CLIC4, RANBP9, TGFB2, HIPK3, BMPR1B, AVL9, TNFRSF1A, CADPS, MAOB, FLOT1, PALLD, SLC1A3, MFHAS1, KCNQ3, TOP1, FOXJ3, ADCY9, PNPLA8, BMPR2, EFR3A, SAMD4A, RGL1, ANKRD12, PKD2, DAP3, BRD4, TAF15, ATXN7L1, ITGA6, SMURF2, AFF4, TMTC1, PTPN9, MAP2K4, NRP1, HOPX, KLF7, PIP4K2A, DENND4A, PFKFB3, WASF2	IRAK2, SLC1A3, NHSL1
Human Poly I:C	Downregulated	MAPRE2, CYB5B, RPS6KA5, PRRC2B, PSMG4, PSD3, FAM189A2, SF1, DNER, ITPR2, RGS20, PLCE1, TUBB2B, LRRC3B, ENTPD1, RALGAPA2, CDHR3, HILPDA, DOCK4, MAGI1, BAALC, FAT3, C3orf70, SLC7A2, TRIM9	FGFR2, CADM2, FRMD4A, NCAM2, CDH10, CTNND2, ADGRB3, MAP2K6, FRY, NRXN1, GRIA2, FAT3, GRM3, MDGA2, ETV4, GRID2, BRINP3, NNAT, LUZP2

Supplementary Figure 26. Comparison of treatment-induced changes with astrocyte subpopulation marker genes

(a) Determination of concordant expression between treatment-induced genes and astrocyte subpopulation-specific markers. P-value was determined by two-sided Fisher's exact test (when the number of genes was < 1,000) and two-sided Chi-square test (when the number of genes was ≥ 1,000) using genes up or downregulated in each treatment and each astrocyte cluster from four single-cell RNA-seq studies[90]. We used Bonferroni correction for multiple comparisons.

(b) Poly I:C and TNFα treatment of human astrocytes induced gene expression changes concordant with markers for the Tsai ast3 cluster and the Schwartz cluster1. Shared genes are listed, including the well-known reactive astrocyte markers CD44 and GFAP in the upregulated group.
