# Supplementary material for: Conservation and divergence of vulnerability and responses to stressors between human and mouse astrocytes
Source: Nat Commun. 2021 Jun 25;12:3958. doi: 10.1038/s41467-021-24232-3 (PMC8233314; doi:10.1038/s41467-021-24232-3)
Supplement: Supplementary file 2 — Description of Additional Supplementary Files [file 41467_2021_24232_MOESM2_ESM.pdf]

Description of Additional Supplementary Files

File Name: Supplementary Data 1
Description: Gene expression (RPKM) of acutely purified, serum-free culture, and serum-selected culture of human astrocytes.

File Name: Supplementary Data 2
Description: GO terms associated with genes differentially expressed by serum-free cultured and acutely purified human astrocytes and hypoxia, poly I:C, and TNFα treatment-induced genes.

File Name: Supplementary Data 3
Description: GO terms associated with genes differentially expressed by human and mouse astrocytes.

File Name: Supplementary Data 4
Description: Top 20 hub genes in the protein-protein interaction networks of genes with higher expression in human or mouse astrocytes. Genes are ranked by the number of connections in the protein-protein interaction networks. Those with the highest number of connections are considered hub genes.

File Name: Supplementary Data 5
Description: Gene expression (RPKM) of grafted human astrocytes and host mouse astrocytes.

File Name: Supplementary Data 6
Description: Differentially expressed genes between xenografted host mouse and naïve mouse astrocytes. Genes with FDR < 0.05 and average RPKM > 0.1 are shown. Unpaired t-test followed by post hoc multiple comparison adjustment using the Benjamini and Hochberg FDR method.

File Name: Supplementary Data 7
Description: GO terms enriched in genes differentially expressed by xenografted host mouse and naïve mouse astrocytes.

File Name: Supplementary Data 8
Description: Age of patients and mice used in RNA-seq comparison of astrocyte gene expression across species.

File Name: Supplementary Data 9.
Description: WGCNA coexpression modules, member genes, and kMEs.

File Name: Supplementary Data 10
Description: Gene expression (RPKM) of hypoxia-, poly I:C-, and TNFα-treated astrocytes.

File Name: Supplementary Data 11
Description: Differentially expressed genes induced by hypoxia, poly I:C, and TNFα treatment. The DESeq2 package was used to calculate differential gene expression and statistical significance.

File Name: Supplementary Data 12
Description: Expression of cell death-associated genes by human and mouse astrocytes. The top 10 cell death-associated genes are based on Sharma et al. The RPKM of cell death-associated genes in all our experimental conditions (acutely purified, serum-selected culture, serum-free culture, xenograft, host, hypoxia, poly I:C, TNFα-treated, and untreated control astrocytes from both human and mouse) are listed.

File Name: Supplementary Data 13
Description: Differentially expressed genes in neurons treated with control and hypoxia-treated ACM. Genes with FDR < 0.05 are shown. The DESeq2 package was used to calculate differential gene expression and statistical significance.
