# Supplementary material for: Conservation and divergence of vulnerability and responses to stressors between human and mouse astrocytes
Source: Nat Commun. 2021 Jun 25;12:3958. doi: 10.1038/s41467-021-24232-3 (PMC8233314; doi:10.1038/s41467-021-24232-3)
Supplement: Supplementary file 5 — Supplementary Data 3 [file 41467_2021_24232_MOESM5_ESM.docx]

**Supplementary Table 3. Gene ontology (GO) terms enriched in genes differentially expressed by human and mouse astrocytes^a^**

| GO term | FDR |
| --- | --- |
| Higher in human |  |
| Defense response | 0.0042 |
|  |  |
| Higher in mouse |  |
| Cellular process | 0.0002 |
| Metabolic process  *Regulation of ph* | 0.0003 |
| Regulation of phosphorylation  R | 0.0023 |
| Regulation of phosphate metabolic process | 0.0035 |
| Regulation of catalytic activity | 0.0035 |
| Regulation of kinase activity | 0.0072 |
| Regulation of protein phosphorylation | 0.0088 |
| Negative regulation of cellular process | 0.0088 |
| Negative regulation of cellular metabolic process | 0.0094 |
| Regulation of protein modification process | 0.0094 |
| Cellular metabolic process | 0.0094 |
| Regulation of transferase activity | 0.0094 |
| Organic substance metabolic process | 0.0094 |
| Catabolic process | 0.0098 |
| Regulation of cellular protein metabolic process | 0,0110 |
| Nitrogen compound metabolic process | 0.0142 |
| Negative regulation of biological process | 0.0145 |
| Regulation of protein kinase activity | 0.0165 |
| Negative regulation of metabolic process | 0.0240 |
| Regulation of triglyceride biosynthetic process | 0.0240 |

^a^We used genes with FDR<0.05 and percentile ranking difference between species >0.4 for GO term analyses. Top 20 terms ranked by FDR (or all terms if there are fewer than 20 enriched terms) are shown.
