# Supplementary material for: Conservation and divergence of vulnerability and responses to stressors between human and mouse astrocytes
Source: Nat Commun. 2021 Jun 25;12:3958. doi: 10.1038/s41467-021-24232-3 (PMC8233314; doi:10.1038/s41467-021-24232-3)
Supplement: Supplementary file 6 — Supplementary Data 4 [file 41467_2021_24232_MOESM6_ESM.docx]

**Supplemental Table 4. Top hub genes in the protein-protein interaction networks of genes with higher expression in human or mouse astrocytes**

| \| **Higher in human astrocytes** \| \| \| --- \| --- \| \| **Gene** \| **Number of connections** \| \| IL6 \| 38 \| \| TLR4 \| 27 \| \| UBA52 \| 25 \| \| IL1B \| 20 \| \| C3 \| 19 \| \| CCR1 \| 16 \| \| CD40 \| 14 \| \| CXCL5 \| 14 \| \| SPP1 \| 14 \| \| CX3CR1 \| 13 \| \| UBE2L6 \| 12 \| \| CXCL16 \| 11 \| \| IFIT1 \| 11 \| \| TIMP1 \| 11 \| \| CXCL3 \| 10 \| \| CYBB \| 10 \| \| GNAS \| 10 \| \| HIST1H2BK \| 10 \| \| NT5E \| 10 \| \| CD4 \| 9 \| | \| **Higher in mouse astrocytes** \| \| \| --- \| --- \| \| **Gene** \| **Number of connections** \| \| Akt1 \| 38 \| \| Mki67 \| 17 \| \| Aurkb \| 15 \| \| Polr2i \| 15 \| \| Hist1h4j \| 13 \| \| Hist3h2a \| 13 \| \| Cenpa \| 12 \| \| Naa38 \| 12 \| \| Ndufb7 \| 12 \| \| Cd68 \| 11 \| \| Isg15 \| 11 \| \| Mrpl12 \| 11 \| \| Stub1 \| 11 \| \| Trmt1 \| 11 \| \| Fbxw5 \| 10 \| \| Ndufa7 \| 10 \| \| Nedd4 \| 10 \| \| Noc4l \| 10 \| \| Ppan \| 10 \| \| Vcam1 \| 10 \| |
| --- | --- | --- | --- | --- | --- | --- | --- | --- | --- | --- | --- | --- | --- | --- | --- | --- | --- | --- | --- | --- | --- | --- | --- | --- | --- | --- | --- | --- | --- | --- | --- | --- | --- | --- | --- | --- | --- | --- | --- | --- | --- | --- | --- | --- | --- | --- | --- | --- | --- | --- | --- | --- | --- | --- | --- | --- | --- | --- | --- | --- | --- | --- | --- | --- | --- | --- | --- | --- | --- | --- | --- | --- | --- | --- | --- | --- | --- | --- | --- | --- | --- | --- | --- | --- | --- | --- | --- | --- | --- |
