# Supplementary material for: Conservation and divergence of vulnerability and responses to stressors between human and mouse astrocytes
Source: Nat Commun. 2021 Jun 25;12:3958. doi: 10.1038/s41467-021-24232-3 (PMC8233314; doi:10.1038/s41467-021-24232-3)
Supplement: Supplementary file 9 — Supplementary Data 7 [file 41467_2021_24232_MOESM9_ESM.docx]

**Supplementary Table 7. Gene ontology (GO) terms enriched in genes differentially expressed by xenografted host mouse and naïve mouse astrocytes^a^**

| GO term | FDR |
| --- | --- |
| Higher in xenografted host mice |  |
| Translation | 0.0118 |
| Peptide metabolic process | 0.0118 |
| Cellular amide metabolic process | 0.0118 |
| Amide biosynthetic process | 0.0118 |
|  |  |
| Higher in naïve mice |  |
| Cellular process | 0.0152 |
| Generation of neurons | 0.0174 |
| Neuron differentiation | 0.0336 |

^a^We used genes with FDR<0.05 and average RPKM>0.1 for GO term analyses. All GO terms with FDR<0.05 are shown.
