# Supplementary material for: Conservation and divergence of vulnerability and responses to stressors between human and mouse astrocytes
Source: Nat Commun. 2021 Jun 25;12:3958. doi: 10.1038/s41467-021-24232-3 (PMC8233314; doi:10.1038/s41467-021-24232-3)
Supplement: Supplementary file 10 — Supplementary Data 8 [file 41467_2021_24232_MOESM10_ESM.docx]

**Supplementary Table 8. Age of patients and mice used in RNA-seq comparison of astrocyte gene expression across species***

| Patients/mice |  | Age | Sex |
| --- | --- | --- | --- |
| Human patients |  |  |  |
| 17 patients |  | 17-20 GW | 7F10M |
| 3 patients |  | 8-18 years | 2F1M |
| 9 patients |  | 19-63 years | 4F5M |
|  |  |  |  |
| Mice |  |  |  |
| Litter 1 |  | 7 days | Both |
| Litter 2 |  | 7 days | Both |
| Litter 3 |  | 1 month | Both |
| Litter 4 |  | 4 months | Both |
| Litter 5 |  | 7 months | Both |
| Litter 6 |  | 9 months | Both |

* GW, gestational week. F, female. M, male. Patients 8-63 years were used in Figure 1G, H, I, J. Patients 8-63 years and gestational week 17-20 were used in Figure 2H. Gestation week 17-20 fetal samples were used in Figure 1D, E, F, 2F, G, 3-8.
