# Supplementary material for: Conservation and divergence of vulnerability and responses to stressors between human and mouse astrocytes
Source: Nat Commun. 2021 Jun 25;12:3958. doi: 10.1038/s41467-021-24232-3 (PMC8233314; doi:10.1038/s41467-021-24232-3)
Supplement: Supplementary file 15 — Supplementary Data 13 [file 41467_2021_24232_MOESM15_ESM.docx]

**Supplementary Table 13. Differentially expressed genes in neurons treated with control and hypoxia-treated ACM.**

| Gene | Log2 Fold Change | Padj |
| --- | --- | --- |
| Human Hypoxia ACM |  |  |
| Rn7sk | -1.4427182 | 8.15E-32 |
| Bc1 | -2.1271272 | 4.43E-31 |
| Gm24187 | -1.0162562 | 2.63E-14 |
| Gm25911 | -0.9354146 | 0.00190508 |
|  |  |  |
| Mouse Hypoxia ACM |  |  |
| Rn7sk | -1.302798 | 2.84E-11 |
| Gm24187 | -0.8218714 | 2.64E-05 |
| Rps2-ps6 | -1.2585731 | 0.00330946 |
