# Supplementary material for: Conservation and divergence of vulnerability and responses to stressors between human and mouse astrocytes
Source: Nat Commun. 2021 Jun 25;12:3958. doi: 10.1038/s41467-021-24232-3 (PMC8233314; doi:10.1038/s41467-021-24232-3)
Supplement: Supplementary file 16 — Reporting Summary [file 41467_2021_24232_MOESM16_ESM.pdf]

nature research

Corresponding author(s): Ye Zhang
Last updated by author(s): May 13, 2021

Reporting Summary

Nature Research wishes to improve the reproducibility of the work that we publish. This form provides structure for consistency and transparency in reporting. For further information on Nature Research policies, see our Editorial Policies and the Editorial Policy Checklist.

Statistics

For all statistical analyses, confirm that the following items are present in the figure legend, table legend, main text, or Methods section.

n/a	Confirmed	
☐	☒	The exact sample size (*n*) for each experimental group/condition, given as a discrete number and unit of measurement
☐	☒	A statement on whether measurements were taken from distinct samples or whether the same sample was measured repeatedly
☐	☒	The statistical test(s) used AND whether they are one- or two-sided<br>*Only common tests should be described solely by name; describe more complex techniques in the Methods section.*
☐	☒	A description of all covariates tested
☐	☒	A description of any assumptions or corrections, such as tests of normality and adjustment for multiple comparisons
☐	☒	A full description of the statistical parameters including central tendency (e.g. means) or other basic estimates (e.g. regression coefficient) AND variation (e.g. standard deviation) or associated estimates of uncertainty (e.g. confidence intervals)
☐	☒	For null hypothesis testing, the test statistic (e.g. *F*, *t*, *r*) with confidence intervals, effect sizes, degrees of freedom and *P* value noted<br>*Give P values as exact values whenever suitable.*
☒	☐	For Bayesian analysis, information on the choice of priors and Markov chain Monte Carlo settings
☒	☐	For hierarchical and complex designs, identification of the appropriate level for tests and full reporting of outcomes
☐	☒	Estimates of effect sizes (e.g. Cohen's *d*, Pearson's *r*), indicating how they were calculated

Our web collection on statistics for biologists contains articles on many of the points above.

Software and code

Policy information about availability of computer code

Data collection	Images were taken using Zeiss Zen (Zen 2.3 pro).
Data analysis	Images were analyzed using FIJI (Version 2.0.0-rc-54/1.51h) and Photoshop CS5.<br>RNA-Seq analysis was performed in RStudio (Version 1.3.1093) using the packages of EdgeR (3.32.0), Limma (3.46.0) and DESeq2 (1.30.0).<br>GO analysis was performed using string-db.org (https://string-db.org).<br>All graphical plots were made by GraphPad Prism 8.<br>Seahorse respiration assay analysis were performed using Agilent Seahorse Wave software.<br>All diagrams were made using Biorender (https://biorender.com).

For manuscripts utilizing custom algorithms or software that are central to the research but not yet described in published literature, software must be made available to editors and reviewers. We strongly encourage code deposition in a community repository (e.g. GitHub). See the Nature Research guidelines for submitting code & software for further information.

Data

Policy information about availability of data

All manuscripts must include a data availability statement. This statement should provide the following information, where applicable:

- Accession codes, unique identifiers, or web links for publicly available datasets
- A list of figures that have associated raw data
- A description of any restrictions on data availability

We deposited all RNA-seq data to the Gene Expression Omnibus under accession number GSE147870. All relevant data are available from the authors without restrictions. The code used to generate RPKM and DEG list has been deposited to Github (https://github.com/Jiwen-Li/RPKM_DESeq2_human_mouse).

Field-specific reporting

Please select the one below that is the best fit for your research. If you are not sure, read the appropriate sections before making your selection.

- [x] Life sciences
- [ ] Behavioural & social sciences
- [ ] Ecological, evolutionary & environmental sciences

For a reference copy of the document with all sections, see nature.com/documents/nr-reporting-summary-flat.pdf

Life sciences study design

All studies must disclose on these points even when the disclosure is negative.

Sample size	We performed power analysis with the R package pwr2 and considered sample size of similar experiments we performed in previous studies to predetermine sample size.
Data exclusions	No data were excluded from analyses
Replication	Data comes from a minimum of three independent experiments unless otherwise stated. All attempts at replication were successful
Randomization	Samples were randomly assigned to treatment and control groups
Blinding	Images were analyzed when the experimenter was blinded to the treatment condition and species (for human-mouse comparisons) when possible with the following exceptions: in the ROS survive experiments and the NFκB p65 staining experiment, cell morphologies from humans and mice are distinct and therefore blinding of the species of the cells was not possible. The experimenter was not blinded during image collection, because images of control and treated cells were always taken at the same time using the same microscope with identical settings such as exposure time and gain.

Reporting for specific materials, systems and methods

We require information from authors about some types of materials, experimental systems and methods used in many studies. Here, indicate whether each material, system or method listed is relevant to your study. If you are not sure if a list item applies to your research, read the appropriate section before selecting a response.

Materials & experimental systems

n/a	Involved in the study	
☐	☒	Antibodies
☒	☐	Eukaryotic cell lines
☒	☐	Palaeontology and archaeology
☐	☒	Animals and other organisms
☐	☒	Human research participants
☒	☐	Clinical data
☒	☐	Dual use research of concern

Methods

n/a	Involved in the study	
☒	☐	ChIP-seq
☒	☐	Flow cytometry
☒	☐	MRI-based neuroimaging

Antibodies

Antibodies used	Antibodies against human nuclei protein (Chemicon, MAB1281, 1:500), human GFAP (Sternberger, SMI21, 1:500), NFκB p65 (Cell Signaling Technology; 8242, 1:200), CD45 (BD550539, 1:600), CD90 (BD550402, 1:600), HepaCAM (R&D Systems, MAB4108, 1:600)
Validation	The antibody against human nuclei protein has been validated by the manufacturer (https://www.emdmillipore.com/US/en/product/Anti-Nuclei-Antibody-clone-235-1,MM_NF-MAB1281) and a previous study (https://www.cell.com/cell-stem-cell/fulltext/S1934-5909(12)00707-2?_returnURL=https%3A%2F%2Flinkinghub.elsevier.com%2Fretrieve%2Fpii%2FS1934590912007072%3Fshowall%3Dtrue).<br><br>The antibody against human GFAP has been validated by the manufacturer (https://www.biolegend.com/en-us/search-results/anti-gfap-antibody-11057) and a previous study (https://www.cell.com/cell-stem-cell/fulltext/S1934-5909(12)00707-2?_returnURL=https%3A%2F%2Flinkinghub.elsevier.com%2Fretrieve%2Fpii%2FS1934590912007072%3Fshowall%3Dtrue).<br><br>Antibodies against CD45, CD90, and HepaCAM have been validated in our previous study (supplementary information, ref 24).

Animals and other organisms

Policy information about studies involving animals; ARRIVE guidelines recommended for reporting animal research

Laboratory animals	We used male and female C57BL6 mice at postnatal day 1-3, and male and female Rag2-knockout mice at neonatal and adult stages

Wild animals	This study did not involve wild animals
Field-collected samples	This study did not involve the samples collected from field.
Ethics oversight	All animal experimental procedures were approved by the Chancellor's Animal Research Committee at the University of California, Los Angeles and conducted in compliance with national and state laws and policies.

Note that full information on the approval of the study protocol must also be provided in the manuscript.

Human research participants

Policy information about studies involving human research participants

Population characteristics	Gestation week 17-20. Both females and males. Samples from patients with genetic disorders such as Down's Syndrome were excluded from the study when known
Recruitment	Women undergoing elective pregnancy termination during gestation week 17-20 were recruited in the clinic with informed consent after the decision for elective pregnancy termination was made. No self selection bias that would affect the results of the study was noted.
Ethics oversight	UCLA Office of the Human Research Protection Program

Note that full information on the approval of the study protocol must also be provided in the manuscript.
